# Atomistic, macromolecular model of the *Populus* secondary cell wall informed by solid-state NMR

**DOI:** 10.1126/sciadv.adi7965

**Published:** 2024-01-03

**Authors:** Bennett Addison, Lintao Bu, Vivek Bharadwaj, Meagan F. Crowley, Anne E. Harman-Ware, Michael F. Crowley, Yannick J. Bomble, Peter N. Ciesielski

**Affiliations:** ^1^ Renewable Resources and Enabling Sciences Center, National Renewable Energy Laboratory, Golden, CO, USA.; ^2^ Chemistry Department, Colorado School of Mines, Golden, CO, USA.; ^3^ Biosciences Center, National Renewable Energy Laboratory, Golden, CO, USA.

## Abstract

Plant secondary cell walls (SCWs) are composed of a heterogeneous interplay of three major biopolymers: cellulose, hemicelluloses, and lignin. Details regarding specific intermolecular interactions and higher-order architecture of the SCW superstructure remain ambiguous. Here, we use solid-state nuclear magnetic resonance (ssNMR) measurements to infer refined details about the structural configuration, intermolecular interactions, and relative proximity of all three major biopolymers within air-dried *Populus* wood. To enhance the utility of these findings and enable evaluation of hypotheses in a physics-based environment in silico, the NMR observables are articulated into an atomistic, macromolecular model for biopolymer assemblies within the plant SCW. Through molecular dynamics simulation, we quantitatively evaluate several variations of atomistic models to determine structural details that are corroborated by ssNMR measurements.

## Teaser

ssNMR observables inform construction and evaluation of atomistic macromolecular models of the plant secondary cell wall.

## INTRODUCTION

Lignocellulosic biomass is one of the largest CO_2_ sinks and source of renewable carbon on earth and has attracted major attention as a feedstock for renewable fuels, chemicals, and materials (*1*, *2*). The lignified plant secondary cell wall (SCW), the primary source of mass in lignocellulosic biomass, is composed of a complex and heterogeneous framework of three major biopolymers: cellulose, hemicelluloses, and polyaromatic lignin (*3*, *4*). The structure of these polymers and emergent properties that arise from their complex interaction compose a lignocellulosic material with mechanical integrity and robust structural features that impart impressive recalcitrance to enzymatic, chemical, and mechanical deconstruction methods (*5*, *6*).

**Fig. 1.**
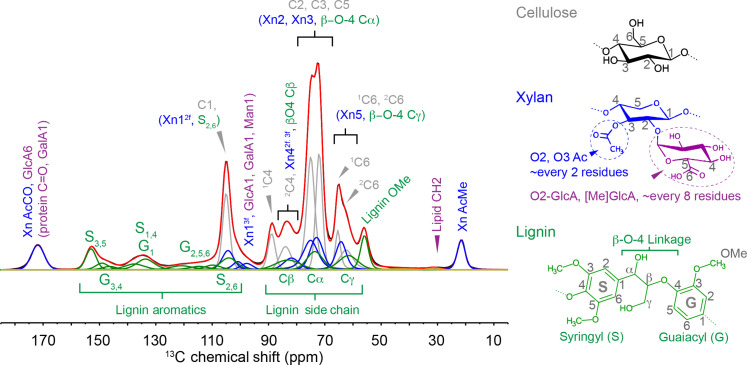
1D ^1^H-^13^C MultiCP-MAS ssNMR provides a broad overview of lignified *Populus* SCWs. General assignment regions are indicated for the predominant constituent polymers cellulose (gray), xylan (blue), and lignin (green), along with representative structures and their assignments indicated with color-coding. Chemical shift information and peak profiles extracted from a combined set of 1D and 2D ssNMR data were used to inform spectral deconvolution efforts used throughout this work. The raw data are shown in black, and the resultant fit is shown in red. C, cellulose; Xn, xylan; Ac, acetyl; GalA, galacturonic acid (pectin); GlcA, α-d-glucuronic acid; [4OMe]GlcA, (4-O-methyl)-α-d-glucuronic acid.

Solving the macromolecular puzzle of lignocellulose structure/property relationships holds the key to efficiently disentangle and deconstruct biomass for conversion to fuels and chemicals and to accelerate development of advanced lignocellulose-derived materials (*7*). Molecular dynamics (MD) simulation is a powerful tool to quantitatively assess important attributes of a system, including thermodynamic properties and mechanical behavior, as a function of the composition, arrangement, and connectivity of the atoms in a system (*8*, *9*). In principle, MD simulations enable the in silico exploration of atomistic design strategies and their impact on emergent properties. However, large-scale prototype molecular models of the lignified plant SCW containing all three major biopolymers are extremely sparse. A major impediment to realistic MD simulations of SCW biopolymer assemblies is the lack of experimentally validated, quantitative details describing the relative atomic positions and conformations of molecules, which are necessary to confidently construct realistic molecular models. For example, several insightful studies used a three-component model of the softwood SCW to investigate nanoscale wood-water relationships; however, the molecular models were based on largely qualitative observables derived from the literature rather than a rigorous, quantitative comparison of experimental and modeled molecular proximities (*10*, *11*). While structural data (e.g., x-ray crystallography) are readily available for some biomolecular systems (e.g., proteins and DNA), this is not the case for heterogeneous biopolymer composites that constitute plant cell walls. Solid-state nuclear magnetic resonance (ssNMR) methods are capable of extracting structural information of heterogeneous materials in situ in their native and unaltered states (*12*–*14*). Incremental progress has been made toward understanding hardwood (*15*–*19*), softwood (*19*–*21*), and grassy (*22*–*24*) SCW superstructure in the past decade using ssNMR methods on ^13^C-enriched biomass. A major breakthrough demonstrated that most xylan within the SCW of plants is decorated with acetyl or glucuronoyl groups on every other xylose unit (Fig. 1), thereby potentially enabling xylan to form a distinct hydrophilic surface for cellulose binding and a hydrophobic surface for lignin interaction, assuming that xylan adopts an extended twofold (two xylose units per 360° turn) orientation (*25*–*27*). It was then suggested through a multitude of two-dimensional (2D) and 3D ssNMR techniques applied to model and engineered hardwoods that xylan likely adopts an extended conformation down the length of the polymer when bound to cellulose microfibrils; however, xylan can adopt a range of disordered threefold helical screw–like structures when unbound (*15*–*17*). MD simulations have also been key in supporting this hypothesis (*25*, *28*, *29*). A separate research group applied magic angle spinning ssNMR methods to probe lignin-saccharide interactions within the SCWs of model lignocellulosic feedstocks. Detailed results informed a revised conceptual model of lignin-polysaccharide packing within grassy SCWs in which lignin self-aggregates into nanoscale domains and retains extensive surface-area contact with xylan, but limited direct contact with cellulose microfibrils was suggested (*22*). In a follow-up study on woody tissue, it was shown that polymer mixing is more homogeneous on the nanoscale compared to grasses, and xylan/lignin interactions were most dominant (*19*). Supporting some of these conclusions for hardwoods, we used a selective 1D ^13^C-^13^C spin-diffusion ssNMR method to better understand spatial arrangements and polymer-polymer interactions within biomolecules, including hardwood plant SCWs, in which tight lignin-xylan surface interaction was confirmed for *Populus* woody stems (*18*).

To build upon these findings and translate the current SCW architectural understanding into an atomistic representation, the hardwood SCW architecture must be quantitatively characterized to provide experimental points of validation or rejection to enable construction of realistic molecular models. Here, we present quantitative insights into the relative proximities of biopolymers in air-dried *Populus* wood derived from ssNMR to experimentally guide the construction of representative in silico plant SCW models. By combining quantitative ^13^C excitement with selective 1D ^13^C-^13^C spin-diffusion measurements, we estimate the extent of polymer-polymer interaction within the hardwood SCW whereby we determine the relative percentage of each ^13^C environment that has gained ^13^C magnetization during a variable spin-diffusion period. We also estimate interpolymer distances within the hardwood SCW. These findings, along with other structural features elucidated by previous studies, are articulated into molecular models that are compared against experimentally derived constraints.

## RESULTS

### ssNMR quantitatively reveals spatial arrangement of biopolymers in air-dried *Populus* wood

A 1D ^1^H-^13^C cross-polarization magic angle spinning (CP-MAS) ^13^C ssNMR spectrum (Fig. 1) provides an overview of the *Populus* SCW and its constituent polymers. ^13^C resonances arising from cellulose, acetylated xylan, and lignin are easily identified in high abundance, while protein, pectin, and lipid signals are sparse. Neutral carbohydrate backbone signals generally reside between 60 and 110 parts per million (ppm). In our case, this region is dominated by cellulose (~45%), but acetylated xylan (~20%) and other polysaccharides including non-xylose hemicelluloses and pectin units also contribute to the signal intensity (~5%). The ^13^C ssNMR spectra of cellulose within plant cell walls give rise to two unique glucose environments. Specifically, the cellulose C4 site shows two distinct environments at 89 ppm (sharp) and 84 ppm (broad), and the C6 site also has two components centered at 65 ppm (sharp) and 62 ppm (broad). To be consistent with Dupree *et al.* (*15*), we refer to these environments as domain 1 (^1^C4, ^1^C6) and domain 2 (^2^C4, ^2^C6) because, as our data confirm, both cellulose environments appear at the cellulose fibril surface in the dense hardwood secondary wall environment [see fig. S35 and (*15*)]. Elsewhere, domain 1/domain 2 signals are assigned as crystalline/amorphous or interior/surface, or based on their C6 hydroxymethyl orientation (*30*). Evidence of abundant acetylated xylan is obvious from the acetyl Ac^Me^ and Ac^CO^ at 22 and 170 ppm, respectively. Resolved lignin signals (~25%) can be seen in the aromatic region and from the lignin OMe at 56 ppm. Lignin inter-unit linkages, dominated by β-O-4 Cα, Cβ, and Cγ resonances, are expected in the 60- to 90-ppm range but are not easily visible by 1D ssNMR methods due to their overlap with polysaccharide signals. 2D through-bond and through-space ^13^C-^13^C ssNMR techniques (figs. S4 to S6) helped resolve overlapping signals and extract accurate ^13^C chemical shifts and peak profiles of the constituent carbon types along with representative monomeric structures in Fig. 1. The combined information from 1D and 2D ssNMR methods were subsequently used to inform spectral deconvolution efforts to quantify polymer-polymer through-space interactions.

**Fig. 2.**
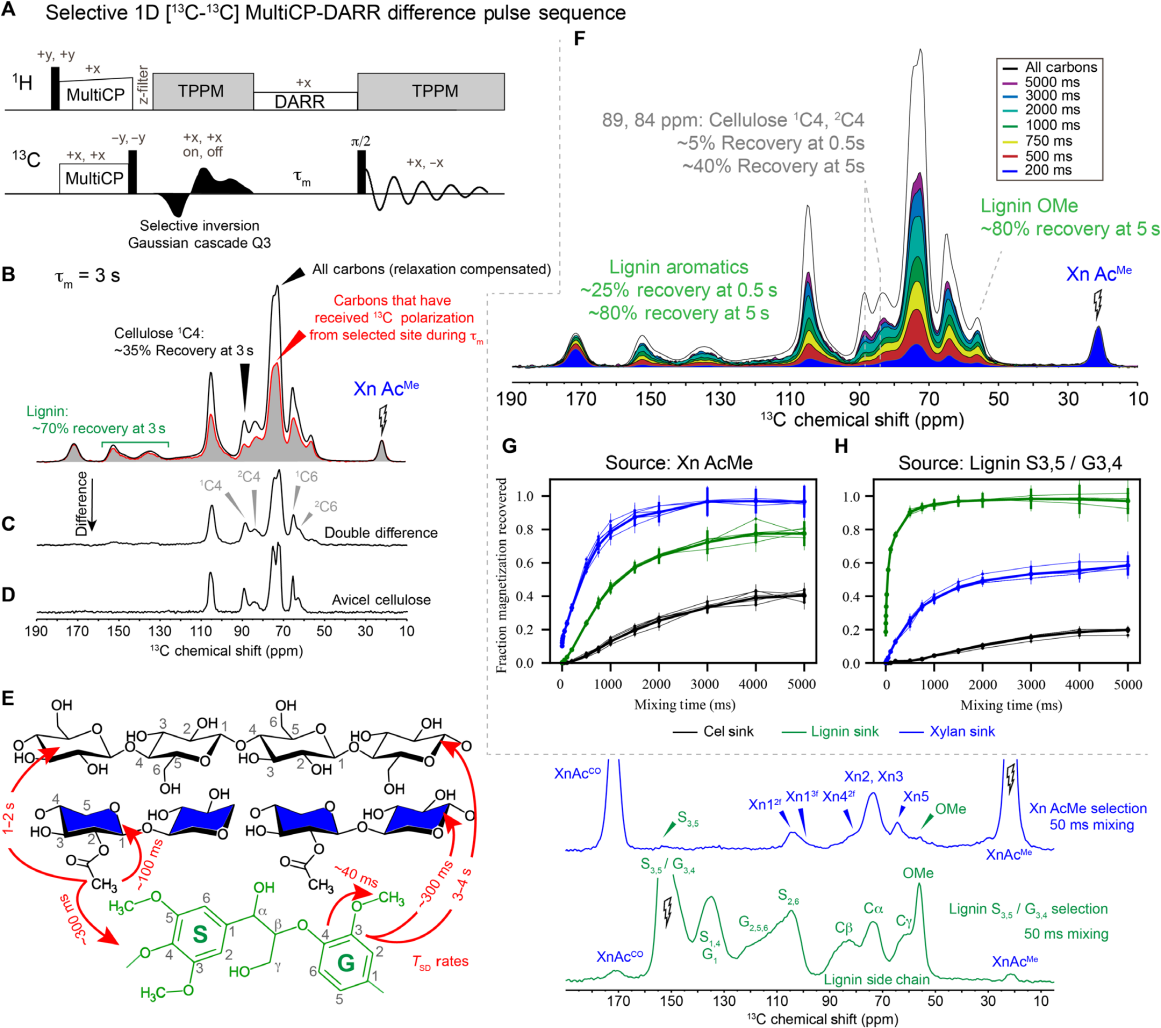
Selective 1D MultiCP-DARR difference data quantify polymer-polymer contacts in the ~1nm length scales. (**A**) Selective MultiCP 1D DARR difference pulse sequence. The experiment results in a selective 1D ^13^C spectrum for which only ^13^C signals (sinks) that have exchanged magnetization with the selected resonance (source) during the variable spin-diffusion period τ_m_ are observed, while all other carbons are not observed because of deconstructive cancelation. (**B**) Example xylan-selected (Ac^Me^ site at 22 ppm) 1D MultiCP-DARR difference spectrum (τ_m_ = 3 s, red trace) is shown next to a nonselective 1D MultiCP-DARR experiment with the same mixing time (relaxation compensated, black trace), which shows all carbons. Comparing the selective and nonselective 1D spectra reveals the relative abundance of ^13^C sites that have or have not received polarization from the selected resonance during the mixing period. (**C**) Double-difference spectrum at 3 s reveals the ^13^C sites that reside outside of ^13^C-^13^C spin-diffusion range from the selected site during τ_m_ = 3 s, resembling that of Avicel cellulose (**D**). (**E**) Cellulose, xylan, and lignin representative structures with carbon positions, along with their ^13^C spectral assignments indicated on sub-spectra obtained from selective 1D MultiCP-DARR difference data. ^13^C-^13^C spin-diffusion rate constants that describe how quickly magnetization moves from source to sink carbons are indicated with red arrows, imparting distance information. (**F**) Stacked plot of xylan-selected 1D MultiCP-DARR difference spectra with τ_m_ ranging from 200 to 5000 ms. Plots are scaled by the intensity of the Xn Ac^Me^ site. (**G** and **H**) Magnetization recovery plots representing calculated xylan-sourced (G) and lignin-sourced (H) through-space contacts at each mixing time out to 5000 ms. Traces from five samples from two biological replicates are shown with thin lines, and their averages are shown with thick lines. Error bars are propagated from the signal to noise.

To gain a more complete understanding of how the constituent polymers are arranged into high-level superstructures within the lignified poplar stem, we collected a series of 2D (Supplementary Materials) and selective 1D (Fig. 2) proton-driven ^13^C-^13^C spin-diffusion experiments ranging from short to long mixing times. Since the ^13^C-^13^C spin-diffusion process is driven by the strength of the dipolar coupling interaction between the exchanging nuclei, which inherently contains distance information, inter-nuclear contacts that are present within approximate distance constraints can be probed by varying the spin-diffusion mixing period τ_m_. Shorter (1 to 100 ms) mixing times typically highlight tighter intramolecular contacts in the upper range of roughly 0.3 nm, while longer (>500 ms) mixing times can be used to identify contacts in the ~0.5- to 1-nm length scales (*31*). As anticipated from prior works (*15*, *18*, *19*, *22*), our data qualitatively confirm subnanometer, through-space contacts between all three polymers (fig. S5). However, there are many possible macromolecular configurations that satisfy constraints specified by polymer composition and observation of subnanometer contacts. For example, this information alone cannot describe the extent of interactions: How much of the lignin is within ~1 nm of acetylated xylan? How much of the cellulose resides within ~1 nm of xylan and lignin?

Here, we aim to reduce these degrees of freedom through quantification of the abundance and length scales of these polymer-polymer spatial interactions. We collected a series of xylan-selective (Xn Ac^Me^, 22 ppm) and lignin-selective (S_3/5_/G_3/4_, ~150 ppm) 1D ^13^C-^13^C spin-diffusion experiments (*18*) to understand the time-dependent equilibration of ^13^C magnetization as it moves from the selected moiety (source) to other carbon environments (sink) that reside within spin-diffusion range (upper limit of ~1 nm) (*31*–*33*). Through these data, it is possible to (i) extract the spin-diffusion time constants (*T*_SD_) that describe how ^13^C polarization exchanges from the source to the sink, which imparts inter-nuclear distance information, and (ii) estimate the percentage of each carbon type that has gained ^13^C polarization from the selected signal during the variable spin-diffusion period. The method was shown to be quantitative by using the MultiCP (*34*) pulse sequence block for initial ^13^C polarization, as validated using ^13^C-enriched standards (see the Supplementary Materials for details). Data are visualized as fraction magnetization recovered (Fig. 2, G and H) for both xylan and lignin magnetization sources.

Xylan-sourced and lignin-sourced selective 1D ^13^C-^13^C spin-diffusion results from five samples from two biological replicates are shown in Fig. 2. The ^13^C-^13^C spin-diffusion time constants *T*_SD_ describing how magnetization moves from the xylan acetate methyl (source) to the xylan backbone, lignin, and cellulose (sinks) were estimated as ~100 ms, ~300 ms, and ~1 to 2 s, respectively (Fig. 2E and figs. S27 to S29). The fast transfer to lignin sites but slow transfer to cellulose carbons reveals that xylan acetyl carbons are closer in space to lignin (~3 to 5 Å) than they are to cellulose (~5 to 10 Å), which is consistent with xylan adopting an extended arrangement with acetate decorations orientated toward lignin when adsorbed to the cellulose surface. Even at short (50 to 100 ms) mixing times, minor lignin signals are clearly visible (Fig. 2 and fig. S6), suggesting that at least a subpopulation of lignin carbons are within 0.3 nm of Xn Ac^Me^ sites.

At moderate mixing times, ^13^C magnetization that originated on Ac^Me^ carbons is actively equilibrating into lignin but does not substantially penetrate into cellulose; at τ_m_ = 500 ms, roughly 25% of all lignin groups but only 5% of all present cellulose carbons have received ^13^C magnetization. For lignin aromatic and methoxy groups, this increases to roughly 35% at 750 ms and 45% at 1 s, and an asymptotic saturation of ~80% is reached in the 3- to 5-s range (Fig. 2G). Meanwhile, the xylan-cellulose contact increases to ~10 to 15% at 1-s, ~25% at 2-s, ~35% at 3-s, and ~40% at 4- to 5-s ^13^C-^13^C spin-diffusion times (Fig. 2G), meaning that roughly 60% of all cellulose resides further than ~1 nm from xylan acetyl carbons (Fig. 2C, double difference).

The above estimates for cellulose magnetization recovery represent the cumulative sum of all cellulose signals derived from spectral deconvolution and are unbiased toward any individual signal. Similarly, lignin recovery represents the sum of resolved lignin signals. Careful analysis of the cellulose buildup profile for each cellulose signal suggests that both domain 1 (crystalline-like) and domain 2 (amorphous-like) cellulose units are represented at the cellulose bundle surface, consistent with an interpretation by Dupree *et al.* (*15*), for Arabidopsis stems, although domain 1 signals may be slightly more prevalent in the fibril interior (fig. S35).

Lignin-selected (S_3/5_/G_3/4_, ~150 ppm) 1D MultiCP-DARR (dipolar-assisted rotational resonance (DARR) difference data reveal lignin/cellulose subnanometer contacts, but the magnetization recovery profile lags behind xylan/cellulose interactions. At 500 and 750 ms spin-diffusion times, ^13^C magnetization has not penetrated substantially (<5%) into the cellulose microfibril, suggesting that direct lignin/cellulose surface interaction is minimal within the poplar secondary wall. At longer mixing times, lignin/cellulose contact is observed more clearly; the percentage of glucan sites that receive magnetization from lignin increases to ~5% at 1-s, ~10% at 2-s, ~15% at 3-s, and ~20% at 4- to 5-s spin diffusion (Fig. 2H).

Further, the spin-diffusion rate constants *T*_SD_ for ^13^C magnetization from the lignin ring carbons into lignin methoxy, xylan acetyl, and cellulose sinks were ~40 ms, ~300 ms, and 3 to 4 s, respectively (Fig. 2E and figs. S30 to S33). These data correspond to interpolymer distances of 3 to 5 Å for lignin/xylan and 5 to >10 Å for lignin to cellulose. Lignin is likely farther away from cellulose compared to the Xn AcMe/cellulose distance based on longer T_SD_ rates. This observation is rationalized if the xylan acetyl decorations point generally away from the cellulose surface toward the lignin layer. Our MD simulations described in subsequent sections further corroborate these experimental findings.

### Magnetization recovery is asymmetric between xylan and lignin sources

Magnetization recovery from lignin (source) to xylan (sink) is asymmetric when comparing to recovery from xylan source to lignin sink; percent of xylan that is recovered at the longest mixing times (~1 nm, 4 to 5 s) only reaches ~60%, not 80% as in the reverse case. The observation of a magnetization recovery asymmetry is curious and was not expected. As will be argued, we believe that the asymmetry is likely a result of multiple factors, including a true architectural asymmetry in which there may be more xylan that is spatially isolated from lignin than there is lignin spatially isolated from xylan.

One possible explanation would be if the magnetization transfer kinetics were asymmetric either due to differing initial ^13^C excitation of lignin and xylan or possibly due to the differing proton densities surrounding xylan and lignin. The former is ruled out because we used MultiCP for initial ^13^C excitation, resulting in uniformly and quantitatively polarized carbons before spin diffusion. We argue that the latter is also an unsatisfying explanation because the ^13^C-^13^C spin-diffusion rate constants for lignin-to-xylan and xylan-to-lignin were symmetric. In support, we confirmed that the MultiCP-DARR-difference method is inherently quantitative and symmetric by applying our methodology on control samples [U-^13^C]-alanine (Cambridge Isotopes) and on microcrystalline peptide formyl-Met-Leu-Phe (CortecNet). Results for both control samples show that ~100% of all sink carbons are recovered for both selected signals (no observed asymmetry), and the spin-diffusion rate constants are nearly identical for each source/sink pairs regardless of which carbon is selected (figs. S1 and S3). Another plausible explanation of the asymmetry is that the selected ^13^C regions near 22 and 170 ppm do not exclusively belong to xylan acetate carbons. Gel-state heteronuclear single-quantum coherence NMR results on ball-milled biomass (fig. S34) and also 2D CP-INADEQUATE ssNMR data (fig. S4) together suggest that roughly 10% of the carbons in the 22- or 170-ppm region belong not to xylan acetate carbons but instead to lipid, pectin, and/or protein moieties. Thus, some of the signal in the 22- and 170-ppm regions that is not recovered after lignin selection and spin diffusion could simply represent other biopolymers that are not spatially close to lignin. This may account for some but not all of the asymmetry; thus, the data suggest that ~80% of lignin is within 1 nm of xylan, while more than 60% but less than ~80% of xylan is within ~1 nm of lignin.

Therefore, our working hypothesis is that (i) some of this observed asymmetry is an artifact of ~10% of the signal in the 22- and 170-ppm regions corresponding not to xylan but to other components (pectin, protein, and lipid), and (ii) the remainder of the asymmetry is truly architectural and due to the existence of a subfraction of acetylated hemicelluloses that are trapped between cellulose bundles and therefore spatially isolated from lignin. This contention is partially supported through subsequently described molecular models and is consistent with other works that also suggest trapped hemicelluloses (*35*–*37*), but the discrepancy serves as a reminder that the reported ssNMR data reflect an ensemble average and may miss architectural heterogeneity or differences among tissue and cell types. Regardless, the observation that upward of 80% of all ssNMR-observable lignin is in direct contact with acetylated xylan strongly suggests that lignin within hardwood SCWs is most likely linearized with small subnanometer domain sizes, as opposed to larger multi-nanometer isolated globules. Well-averaged ^13^C T_1_ values caused by efficient ^13^C-^13^C spin diffusion further corroborate our overall conclusions of extensive polymer mixing on the nanometer length scale (fig. S36). Nanoheterogeneous polymer mixing implied by our results is consistent with Kirui *et al.* (*19*) for hardwoods. Since the four-fiber molecular models failed to replicate this asymmetry, larger models were built with some entrapped hemicellulose.

### ssNMR observables provide crucial metrics for construction and evaluation of SCW atomistic models

Our objective was to construct a periodic molecular structure that is consistent with the following ssNMR conclusions: (i) ^13^C-^13^C spin-diffusion rate constants and approximate interpolymer distances confirm that xylan is predominantly “in the middle” of lignin and cellulose; (ii) xylan in direct contact with cellulose adopts a conformation such that its decorations point generally away from the cellulose surface; (iii) ~80% of all lignin and ~40% of cellulose chains are within ~1 nm of xylan acetyl carbons; (iv) ~60% of all xylan and ~20% of all cellulose carbons are within ~1 nm of lignin ring carbons; and (v) lignin and xylan are most likely of extended morphology, not globular, and lignin/xylan homogeneous domain sizes are generally not larger than ~1 nm in size.

In addition to ssNMR observations, compositional analyses provide further constraints to guide construction of a representative molecular model. These measurements, described in Materials and Methods, include cumulative biopolymer ratios, water content, lignin monomer composition and linkage abundances, and characterization of xylan decoration pattern.

**Fig. 3.**
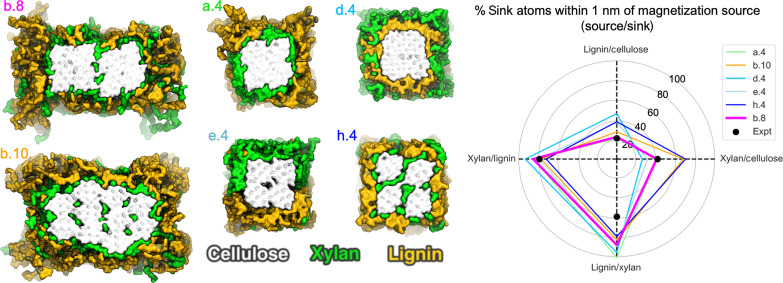
Atomistic SCW periodic model variants and comparison of their proximity analyses with ssNMR spin-diffusion experiments. (**Left**) Snapshots of several molecular model variants for the poplar SCW. The naming convention is as follows: The letter refers to the relative arrangement of hemicellulose and lignin, where “a” denotes a model with all xylan bound on cellulose, and no xylan interspersed with lignin; “b” denotes a model with 70% hemicellulose bound to cellulose, and the remaining 30% interspersed in the surrounding lignin-xylan matrix; “d” denotes a model with lignin-bound cellulose and with hemicellulose as the surrounding layer; “e” denotes a model with phase-separated hemicellulose and lignin both bound to cellulose at the top and bottom, respectively; “h” denotes a model with hemicellulose interspersed within the 18-chain elementary cellulose fibril leaving room for increased direct lignin-cellulose interaction. The number after the decimal denotes the number of 18-chain elementary fibrils in the model. b.8 and b.10 are larger systems constructed with 70% of xylan bound to cellulose, and the remaining 30% interspersed with lignin and consist of eight and ten 18-chain cellulose elementary fibrils, respectively. (**Right**) Calculated percentage of sink atoms within 1 nm of source atoms in the model variants compared to that obtained from ssNMR spin-diffusion experiments depicted on a polar plot. The four dashed lines originating from the center indicate the four ssNMR magnetization metrics, with the experimental values indicated as black dots and the colors indicating the various models.

Despite the wealth of experimental insight described here and in prior studies, the construction of representative atomistic models is stymied by the lack of detailed quantitative metrics pertaining to the relative placements of component biopolymers. Here, we built multiple variations of molecular models and systematically compare them to the criteria described above to determine which macromolecular arrangements provided the best overall agreement with the ssNMR observables. The atomistic models were subjected to MD simulations, and then equilibrated snapshots of the systems were analyzed for reproduction of ssNMR magnetization recovery behavior and consistency with interpolymer distances. A subset of the molecular model variations is presented in Fig. 3 (left), and the entire set is shown in the Supplementary Materials (figs. S37 and S38). Atoms in the molecular model corresponding to the source and sink atoms from ssNMR experiments were identified, and the cumulative number of sink atoms within 1 nm of source atoms was calculated (see the Supplementary Materials). This proximity analysis enables direct comparison of the distance criteria obtained from ssNMR magnetization recovery data, which is presented as a polar plot in Fig. 3 (right).

The simplest model consisted of four, 18-chain elementary cellulose fibrils surrounded by the lignin-xylan polymeric matrix. This model formed a basis for exploring variations in lignin and xylan placement in the SCW, including negative controls that verify the efficacy of the proximity analysis, and to evaluate whether substantial rearrangement of polymers occurs during MD simulations. We did not observe migration or rearrangement of polymers for any of the systems investigated here over the course of the 100-ns simulations (fig. S39), which reinforce the importance of the initial placement during model construction.

Each of the eight (a to h) four-fiber variants (figs. S37 and S38) demonstrate subnanometer interactions as anticipated by prior experimental observations, making it difficult to discern which polymeric arrangement is most correct without additional quantitative metrics. For example, even negative control variants like d.4, in which lignin is deposited in the middle between cellulose and xylan, have subnanometer xylan-cellulose interactions mediated through a lignin layer. Herein lies the key insight—All of these systems including negative controls satisfy the previously published experimental observations of subnanometer through-space interactions between the three major biopolymers; however, the extent of these interactions varies substantially on the basis of the initial relative placement of the biopolymers (figs. S37 and S38). Therefore, the extent of interactions measured in this work establish additional criteria for building and evaluating atomistic molecular models.

Among the four-fiber variants, a.4 had the best agreement with experiment in terms of lignin/cellulose and xylan/cellulose proximities. This variant has all of the xylan bound on cellulose (i.e., no mixing of xylan and lignin in the surrounding matrix). However, experimental data indicate that ~70% xylan is observed in extended, presumably twofold (2_1_) helical screw morphology (fig. S4), suggesting that a fraction of hemicellulose is likely interspersed with lignin. Variant b.4 was built with 70% of the xylan in direct contact with cellulose, and the remaining 30% of the xylan interspersed with the surrounding lignin matrix. While xylan/cellulose proximities were well represented in this model, lignin/cellulose proximities were overestimated. A similar observation was noted for variants c.4, built with xylan randomly interspersed with lignin, and the negative control d.4, built with lignin in direct contact with cellulose and xylan on the outer surface of the lignin. In addition, c.4 and d.4 variants also severely underpredict xylan/cellulose proximity.

Variants d.4 and e.4 were, in part, designed to test direct cellulose/lignin deposition. We note that the extent of direct lignin-cellulose contact within lignocellulose is actively debated. For example, prior molecular modeling results suggest that lignin might interact directly with cellulose preferentially on the hydrophobic face (*38*). Dupree *et al.* (*15*) presented ssNMR evidence that there may be extensive direct lignin/cellulose surface contact within Arabidopsis stems, but other reports claim that direct lignin/cellulose interaction is minimal in grasses (*22*) but somewhat more abundant in woody tissue (*19*). Our molecular models, particularly the negative controls d.4 and e.4, suggest that if substantial lignin is directly deposited onto the cellulose surface, lignin/cellulose proximity is overpredicted relative to our experimental data. Our interpretation of these data is that the cellulose bundle surface is well covered by acetylated hemicelluloses, leaving little room for direct lignin-cellulose contact. Instead, lignin-cellulose subnanometer contacts observed by ^13^C-^13^C spin-diffusion methods are largely mediated through the xylan layer.

**Fig. 4.**
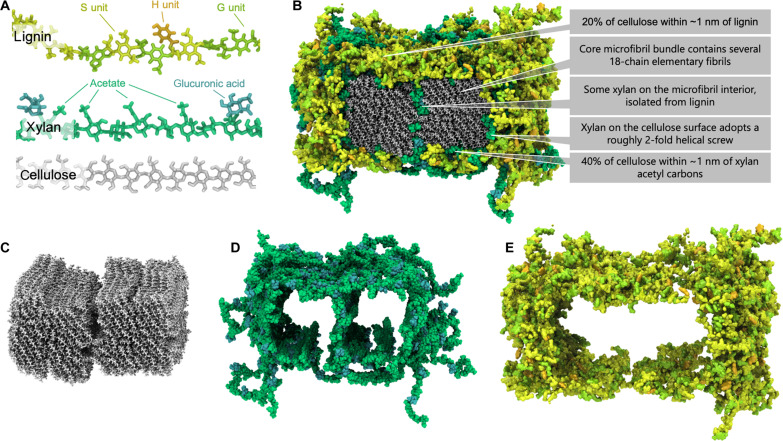
Atomistic model of the lignocellulose assembly within the *Populus* SCW that best represents ssNMR observables. (**A**) Molecular representation of the individual biopolymer constituents. (**B**) Macromolecular assembly of cellulose, xylan, and lignin. The xylan domains on the surface of cellulose adopt an extended configuration with decorations pointing away from the cellulose surface toward lignin. (**C**) Visualization of the cellulosic components only, showing two core bundles comprising four 18-chain elementary fibrils. (**D**) Visualization of the hemicellulose components only shows the nearly complete sheath formed around the cellulose minimizing direct contact between cellulose and lignin; also, some xylan is found between core bundles, isolated from lignin. (**E**) Lignin interacts predominantly with hemicellulose and displays minimal direct contact with cellulose.

Limitations of all four fiber systems include the inability to represent the experimentally observed asymmetry in lignin/xylan and xylan/lignin proximities. This led us to build two larger systems consisting of eight and ten 18-chain elementary fibrils, denoted b.8 and b.10, respectively. Each included a fraction of xylan isolated from lignin and present between the cellulose microfibrils in an effort to rationalize the experimentally observed asymmetry (*35*, *36*). The isolation of a portion of xylan from lignin enables a notable reduction of xylan-sourced magnetization onto lignin sinks for both these systems. These models were also built with 70% xylan bound on cellulose and the remaining xylan entangled with lignin as suggested by ssNMR data (fig. S4) (*19*). Model b.10 overpredicts xylan/cellulose proximities presumably because here xylan is entrapped between elementary 18-chain microfibrils. Ultimately, the eight-elementary fiber system b.8 where xylan is entrapped between larger core cellulose bundles shows the best agreement for xylan-sourced magnetization on cellulose and lignin sinks, lignin-sourced magnetization on cellulose sinks, and the least mismatch with experiment for lignin-sourced magnetization on xylan sinks. This model is presented in greater detail with a summary of its defining features in Fig. 4.

The clustered arrangement of multiple, 18-chain elementary fibrils within the macrofibril core is consistent with studies of SCW synthesis, where multiple cellulose synthase enzymes have been shown to act in concert to produce larger aggregates (*39*). Adjacent elementary cellulose fibrils (i.e., those that were not separated by xylan) within the macrofibril interior exhibit a center-to-center distance of ~2.5 nm, although these tended to further aggregate during MD simulations to form larger, well-ordered domains in the macrofiber core, which agrees well with x-ray and neutron scattering measurements of microfibril size and spacing in hardwoods (*37*, *40*). This feature of aggregated element fibrils also differentiates our model from previously published molecular models of the SCW, wherein individual elementary cellulose fibrils are completely ensheathed with matrixing biopolymers, which prevents their assembly into larger highly ordered bundles (*10*, *41*).

### Acetate group orientations dictate xylan polymer conformation

Our simulations indicate three major conclusions with respect to xylan conformation: (i) Xylan conformation is primarily determined by initial conditions and does not change substantially over the course of MD simulation (fig. S39); (ii) the xylan conformation is better described by acetate group orientation rather than glycosidic torsional angles; and (iii) xylan conformation is only ordered when bound to cellulose, while unbound xylan is primarily disordered. Previous NMR and modeling studies have characterized xylan conformations as twofold (2_1_) or threefold (3_1_) helical screws based on the sum of φ and ψ glycosidic torsion angles of adjacent residues. The 2_1_ conformation of β(1–4) linked glycans is characterized by a φ + ψ sum ~120°, whereas the 3_1_ conformation is characterized by a φ + ψ sum ~50° or 190° (*17*, *25*, *42*, *43*). It has also been suggested that xylan in solution assumes the 3_1_ conformation, while xylan bound on cellulose assumes a 2_1_ conformation driven by the orientation of evenly patterned acetate groups on xylan away from the cellulose surface (*17*, *25*, *43*, *44*). Unlike these previous computational studies, which simulated individual hemicellulose chains on a cellulose surface in a solvated environment, our simulations afford the exploration of xylan conformations in the SCW in its cellulose-bound and unbound states in a representative crowded environment in the presence of lignin.

Xylan conformations over the course of our MD simulations are not clearly described by the sum of backbone glycosidic torsion angles. For example, at φ + ψ equal to 50°, 120°, and 190°, where we would expect to see peaks in populations for 2_1_ or 3_1_ conformation based on previous computational studies, we instead found dips in populations (fig. S39B). This discrepancy may be attributed to the longer xylan polymers (DP40) considered here compared to the shorter (DP9-DP10) xylan polymers investigated by Busse-Wicher *et al.* (*25*) and Mazeau *et al.* (*42*). Busse-Wicher *et al.*’s (*43*) MD simulations of DP16 xylan chains bound to cellulose suggest that the torsion angles of xylan bound to cellulose may not be limited to 120° but also have large populations distributed around 90° and 180°, as we observe in our simulations. In addition, the dense cell wall matrix may preclude xylan from assuming idealized conformations that are observed in more simple systems (*25*, *42*).

**Fig. 5.**
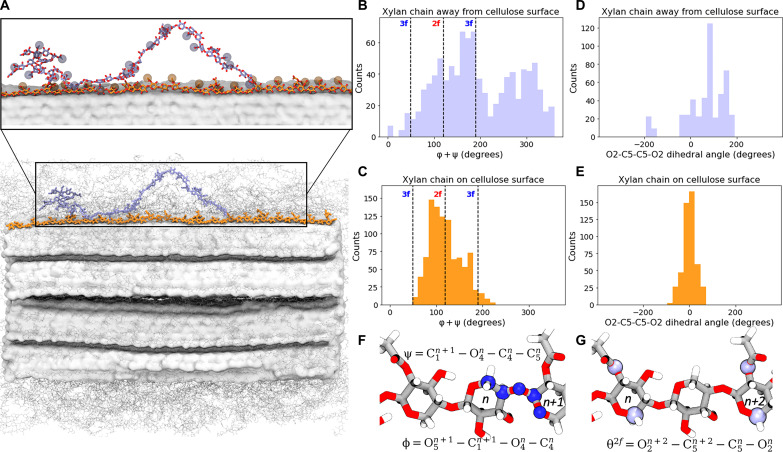
Contrasting conformation of xylan polymers with different proximities to cellulose. (**A**) Xylan bound to cellulose (orange) adopts conformations that enable acetate groups to consistently orient away from the cellulose surface. Unbound xylose (light blue) adopts a more random orientation of acetate groups. Exemplary xylan polymers are shown in licorice representation, with their acetate groups highlighted as spheres, and cellulose is shown as a white surface. (**B**) Unbound xylan φ + ψ distributions are not exclusively 3_1_ and span a large range of values with a peak at ~170^0^. (**C**) For xylan chains with similarly oriented acetate groups, a wide distribution of canonical φ + ψ conformations is observed, although a peak ~100^0^ is prominent. (**D**) θ^2*f*^(O_2_-C_5_-C_5_-O_2_) values for the unbound xylan chain are widely distributed corresponding with the nonuniform acetate group orientations. (**E**) Bound xylan exhibits a narrow θ^2*f*^ distribution centered at 0^0^, which highlights the consistent orientation of acetate groups. Definition of canonical φ + ψ and θ^2*f*^(O_2_-C_5_-C_5_-O_2_) dihedral measurements is portrayed in (**F**) and (**G**), respectively.

Glycosidic torsional angles therefore provide an imprecise metric for xylan conformation in the SCW. To remedy this discrepancy, and since the core hypothesis describing xylan conformation as 2_1_ versus 3_1_ is linked with acetate group orientation on xylan, we propose a redefinition of the metrics used to describe 2_1_ xylan. The θ^2*f*^(O_2_-C_5_-C_5_-O_2_) dihedral involves the acetate-bound oxygen (O_2_ or O_3_) and the C_5_ cyclic carbon atom of acetylated xylose residues (Fig. 5G). Analysis of the θ^2f^ dihedral for xylan polymers over the simulation trajectory showed that xylan in contact with cellulose does assume a linear 2_1_ conformation with acetate groups pointed away from the cellulose surface, while xylan away from cellulose is in a disordered non-2_1_ conformation with acetate groups oriented nonuniformly. This behavior is illustrated in Fig. 5 (A to E).

## DISCUSSION

The ssNMR results presented here for air-dried ^13^C-enriched lignified poplar stems, when interpreted through the lens of atomistic molecular models, substantially advance our understanding of the hardwood plant SCW. Polymer-polymer contacts on the 1-nm length scale, estimated from selective 1D ^13^C-^13^C spin-diffusion measurements, have provided crucial knowledge of molecular arrangements of biopolymers to guide the construction of representative atomistic models of the SCW. Our MD simulations demonstrate that initial polymer placement is extremely crucial when building SCW atomistic models given that polymers do not substantially rearrange even over several hundred nanoseconds of simulated time. Among the many SCW variants with distinct initial polymer placements, the best agreement with ssNMR observables was achieved with model b.8 (Fig. 4), containing core cellulose bundles with some entrapped xylan and the full microbundle encased in a xylan-lignin matrix. The xylan in this matrix is predominantly cellulose bound, while direct lignin-cellulose interaction is minimal and instead is mediated through the xylan layer. Through ssNMR criteria and by redefining the metrics of xylan conformational analysis, we also confirm that bound xylan predominantly orients its acetyl groups away from cellulose and enables hydrophobic interactions with lignin.

There are some limitations to this study that could affect overall results. For example, we observed an intriguing asymmetry between lignin-sourced and xylan-sourced magnetization recovery curves: ~80% of all the lignin resides within ~1 nm of acetate methyl carbons, but ~60% of acetate carbons reside within ~1 nm of lignin. Considering that (i) the data were collected using the quantitative MultiCP (*34*) block for initial ^13^C polarization (fig. S1), (ii) xylan-to-lignin and lignin-to-xylan spin-diffusion time constants are identical (~300 ms) in both directions, and (iii) the asymmetry was not observed on control samples alanine and n-formylmethionine-leucyl-phenylalanine (fMLF) (figs. S1 and S3), we hypothesize that the asymmetry is truly architectural and not an artifact of asymmetric magnetization transfer kinetics. No single molecular model was able to completely reproduce this feature. However, the ssNMR magnetization behavior obtained from the poplar samples is an amalgam of more than one tissue or cell type each with its own distinct biopolymeric arrangement, for example, primary wall and middle lamella, fiber and vessel cells, and other heterogeneities. Structural variations are very probable in the cell walls and a “one-size-fits-all” model is likely not a realistic view of the SCW molecular architecture. A solution to the exact reproduction of this experimental observable from the atomistic scale might necessitate some representation from an ensemble of molecular model variants described in this study. If practical, cell type differentiation before ssNMR, for example, by using laser capture microdissection, could help with distinguishing cell wall architecture variability among differing cell types. Another possible degree of uncertainty emerges from our heavy reliance on spectral deconvolution of 1D spin-diffusion data. Resolved signals like xylan acetate and lignin aromatics are confidently deconvoluted, but peak-fitting of overlapping regions, in particular, the 110- to 60-ppm region, is indeed tricky. While our spectral deconvolution strategy was carefully informed (see the Supplementary Materials) and results are highly consistent across all replicate samples, we acknowledge that this may impart some unspecified level of uncertainty. Further, the ^13^C-enriched poplar stems studied in this work are young. Our data may represent mature wood (the stems are highly lignified and contain little primary wall material, and the polymeric composition and lignin linkage abundances match those in the literature for DN34 poplar), but it would be interesting to see whether the models hold true for secondary wall tissue in more mature trees. In addition, the ssNMR data were obtained on destarched and extracted woody material that was air-dried to ~3 to 8% moisture content. Some degree of irreversible pore collapse due to water removal is expected. While our data suggest that starch and extractive removal does not alter the wood macrostructure (table S7), the results and models should therefore be more applicable for partially dehydrated wood, while their ability to represent never-dried wood warrants future investigation. Phenotypic variability was also not investigated in this work; we collected data on multiple replicates but only one *Populus* species, DN34 hybrid poplar. Like other phenotypes, poplar cell wall architecture may vary throughout the genotypic domain or as a function of other properties including environmental growth conditions as well as the presence of biotic and abiotic stresses.

Despite these limitations, our work to quantitatively inform polymer/polymer interactions and articulate the current understanding of cell wall superstructure into computationally accessible atomistic molecular models represents the culmination of a decade of research. The utility of the molecular models developed here extend beyond improving our basic understanding of plant cell wall architecture. Advances in our understanding of how molecular composition and architecture affect function and conversion will accelerate utilization of lignocellulose for a variety of applications. Many chemical conversion processes require infiltration of the cell wall to promote catalytic deconstruction (*45*). Similarly, structural applications of wood that rely upon adhesives will benefit from improved intra–cell wall diffusion and stronger intermolecular interactions (*46*). Experimental studies of ionic diffusion within wood cell walls have demonstrated that the process is sensitive to macromolecular architecture, water content, and polymer dynamics (*47*, *48*). MD simulation in the context of reliable SCW models can provide a useful mechanistic understanding of molecular and macromolecular phenomena that govern intra–cell wall migration and interaction for a variety of applications. Beyond traditional applications such as biofuels, biochemicals, and lumber, there has been a recent upsurge in lignocellulosic materials for nonconventional applications, such as batteries (*49*), photonic devices (*50*), and super-strong and malleable structural materials (*51*, *52*). These next-generation applications have potential to displace nonrenewable and environmentally detrimental incumbents with sustainable alternatives, and their development will be undoubtedly accelerated by improved molecular models and simulation methods to understand and predict how molecular features of lignocellulose and its derivatives affect bulk properties and functionality (*53*). This approach of building atomistic models with varied spatial arrangements of constituent biopolymers, benchmarked with ssNMR data, presents a robust protocol for the development of realistic and holistic plant cell wall models. These experimentally supported atomistic models lay the foundations for future in silico explorations to gain unprecedented insights into the molecular level determinants of the emergent properties of naturally occurring, treated, and engineered biomass.

## MATERIALS AND METHODS

### Experimental design

The objective of this study was to provide quantitative metrics from ssNMR measurements that can be used to inform the construction and validation of atomistic molecular models of the woody secondary wall. The major tenants therefore include ^13^C enrichment of biomass, ssNMR measurements and analysis, and construction and analysis of atomistic models in silico.

### Preparation of ^13^C-enriched DN34 hybrid poplar woody stems

^13^C-enriched hybrid poplar (DN34) trees were grown in a ^13^C CO_2_ (Sigma-Aldrich) atmosphere using a Percival Scientific (Perry, IA, USA) growth chamber. Trees were initially rooted outside the growth chamber and then transferred into the chamber for 6 weeks under the following growth conditions: 16-hour day cycle, 23°C, 65% relative humidity, 700 ppm ^13^C CO_2_, 8-hour night cycle, 18°C, 65% relative humidity, and 500 ppm ^13^C CO_2_. At the end of the labeling period, the plants were harvested, and leaves, stems, and roots were separated. The level of ^13^C enrichment was ~80% as determined by pyrolysis molecular beam mass spectroscopy (fig. S42). Stems were debarked while wet and then allowed to dry on the benchtop for many days. Debarked stems were then cut into 1- to 2-inch segments, and each segment was milled to 40 mesh using a Wiley knife mill. Five samples from two biological replicates were analyzed after removal of starches and extractives as follows: Milled samples were wrapped inside a standard mesh tea bag, then starches were removed using commercial amylases as described by Decker *et al.* (*54*), and small-molecule polar organics were removed by 95% ethanol extraction using a Soxhlet setup for many hours. Samples were further washed with deionized (DI) water and air-dried (20 to 40% humidity) and then stored in glass vials until NMR analysis. To investigate the structural effects from the destarching and extraction procedure, proximity analysis for two more samples was analyzed from a third biological replicate that was washed with DI water and air-dried but otherwise untreated (table S7).

### 2D ^13^C-^13^C correlation ssNMR for shift assignments and informing spectral deconvolution

To aid in accurate ^13^C chemical shift assignments used for spectral deconvolution and to provide a broad overview of the material, 2D through-bond and through-space ^13^C-^13^C correlation experiments were collected at higher field (600 MHz, 14.1 T) and 2D through-space measurements were collected at lower field (200 MHz, 4.7 T). All (600 MHz) data were collected on a Bruker AvanceIII NMR spectrometer equipped with a PhoenixNMR HXY MAS probe (Loveland, CO) using either a 1.6-mm (30 kHz) or 4-mm (13.5 kHz) probe head operating in HC double-resonance mode, while all 200-MHz (4.7 T) data were collected on a Bruker AvanceIIIHD NMR spectrometer equipped with a 4-mm Bruker HX MAS probe. 2D double-quantum (DQ) CP-Refocused INADEQUATE (*55*, *56*) was collected with the following conditions: 1.6-mm PhoenixNMR probe head, 30-kHz MAS, 128 scan averages, 192 pts. in f1, 2× DQ delays of τ_1_ = 2.5 ms, τ_2_ = 2.1 ms, 5-s recycle delay, 60-kHz sweep width in both dimensions, radio frequency (RF) field strengths of 140 kHz for ^1^H hard pulses and decoupling, 100 kHz for ^13^C hard pulses, and ^1^H-^13^C CP achieved using a square 2-ms 67-kHz spin locking pulse on the ^13^C channel and a 10% ramped pulse on the ^1^H channel matched to the +1 spinning side band. 2D INADEQUATE data were processed in MestreNova using 20-Hz exponential line broadening in the direct dimension, and in the indirect dimension, we used Gaussian apodization (GF = 0.032) paired with −100 Hz exponential line broadening. 2D through-space ^13^C-^13^C correlation experiments (600 MHz) were collected at 13.5-kHz MAS using a 4-mm PhoenixNMR probe head; 32 scan averages and 400 indirect points; 60-kHz sweep widths in both dimensions; and with mixing times of 30, 100, 500, 1000, and 2000 ms using CORD (*57*) recoupling. Hard pulses were 3 and 4 μs for ^1^H and ^13^C, respectively, and CP was achieved using a square 2-ms 57-kHz spin-locking pulse on the ^13^C channel and a 10% ramped pulse on the ^1^H channel matched to the +1 sideband. 2D ^13^C-^13^C DARR was also collected at 200 MHz using a short (50 ms) spin-diffusion mixing time using continuous wave ^1^H recoupling (ω_1_ = ω_r_) to further aid in ^13^C shift assignments and to provide initial linewidth estimates for spectral deconvolution of 1D datasets. Data were collected at 10-kHz MAS with 64 scan averages and 256 indirect points, and 20-kHz sweep widths in both dimensions. 2D through-space spin-diffusion data were typically processed with MestreNova using 20-Hz exponential line broadening in the direct dimension and with a 90° shifted sine bell apodization in the indirect dimension.

### Selective and nonselective 1D MultiCP-DARR

1D ^13^C-^13^C spin-diffusion measurements were collected similar to our previous work (*18*) with some modifications; most notably, we used the MultiCP (*34*) block to obtain quantitative spin-diffusion information. MultiCP was performed using a total of three 1.1-ms CP blocks (square 62-kHz spin-lock on ^13^C, 14% ramped spin-lock step on ^1^H matched to the +1 sideband) separated by 0.8-s *t*_z_ repolarization delays, and a recycle delay of 0.5 s. Three CP steps is fewer than typically used for biomass (*34*), but these conditions were confirmed quantitative by comparing a clean MultiCP ^13^C spectrum with a fully quantitative direct polarization (DP-MAS, 35-s recycle delay) spectrum (fig. S7).

1D ^13^C-^13^C spin-diffusion measurements were collected at 16 increasing mixing times to track the time-dependent equilibration of ^13^C magnetization between the selected and all other ^13^C sites: τ_m_ = 0.001, 2, 5, 10, 20, 50, 100, 200, 500, 750, 1000, 1500, 2000, 3000, 4000, and 5000 ms. At each of the 16 ^13^C-^13^C spin-diffusion mixing times, three experiments were collected: (i) a selective 1D MultiCP-DARR-difference spectrum using Xylan Acetate methyl selection [10-ms Gaussian Cascade Q3 (*58*) selective inversion pulse at 22 ppm], (ii) selective 1D MultiCP-DARR-difference spectrum using Lignin S_3,5_/G_3,4_ selection [5-ms Gaussian Cascade Q3 (*58*) selective inversion pulse centered at 150 ppm], and (iii) a nonselective 1D MultiCP-DARR spectrum was collected to provide a quantitative and relaxation-compensated reference. The latter experiment is effectively the first slice of the conventional 2D DARR (*59*) experiment using MultiCP for initial ^13^C polarization. During the spin-diffusion period τ_m_, ^1^H dipolar coupling was reintroduced using a continuous wave RF pulse matched to the first rotor resonance condition of ω_1_ = 10 kHz. All nonselective 1D MultiCP-DARR experiments were collected with 256 scan averages, while selective 1D MultiCP-DARR-difference data were collected with either 8192 or 4096 scan averages for τ_m_ greater than 1 s or 2048 scan averages for τ_m_ = 1 s or shorter. For control experiments, we applied the same methodology of quantitative MultiCP excitation and selective 1D spin-diffusion measurements on ^13^C-alanine (Cambridge Isotopes) and ^13^C-formyl-Met-Leu-Phe microcrystalline peptide (fMLF, CortecNet). MultiCP conditions were optimized on each sample and validated by comparing 1D MultiCP to fully relaxed ^13^C spectra from direct excitation. For alanine, selective inversion was performed using 10-ms inversion pulses at Ala Cβ and Ala CO, while for fMLF we used 5-ms selective inversion pulses for Phe Cδ,ε near 129 ppm and a 30-ms inversion pulse for Leu Cδ2 at 19 ppm. ^13^C chemical shifts for all measurements were referenced externally to tetramethylsilane (TMS) at 0.0 ppm by setting the downfield adamantane signal to 38.48 ppm (*60*). All spectra were processed with 20-Hz exponential line broadening before Fourier transform, and resulting spectra were individually phased and baseline-corrected before spectral deconvolution. Details of how spectral deconvolution was performed are provided in the Supplementary Materials.

### MD methods

#### Additional considerations for preparation of atomistic molecular models

In addition to ssNMR observations, compositional analyses including biopolymer ratios, water content, lignin monomer composition and linkage abundances, and characterization of xylan decoration pattern provide further constraints to guide construction of a representative molecular model. The relative polymer ratios in the secondary wall were set on the basis of percent dry weights of 45% cellulose, 20% hemicellulose, and 25% lignin (see table S2), which represent the average polymer ratios extracted from a large panel of poplar wood samples (*61*) and closely match the percent dry weight values determined gravimetrically for DN34 poplar stems per NREL standard Laboratory Analytical Procedure “Determination of Structural Carbohydrates and Lignin in Biomass” (NREL LAP TP-510-42618). In dicots, the dominant hemicellulose polysaccharide is a β(1,4)-linked xylan backbone that is heavily substituted with acetyl and glucuronosyl residues at the O-2 and O-3 positions. For most of the xylan backbone, these substitutions typically occur at every other xylosyl unit for acetyl groups or every 8 to 10 residues for glucuronosyl decorations (*16*, *17*, *25*, *26*, *62*, *63*). We therefore built each individual xylan polymer with a degree of polymerization of 40, and with acetyl groups every two residues and glucuronic acid moieties were also placed every eight xylose units to represent the “major domain” of xylan within hardwoods. A schematic model of lignin 20-mers proposed by Ralph *et al.* (*64*) for hardwoods was used to construct the structure of the lignin model, which has an S/G ratio of 1.8/1 and a β-O-4 content of 80%. These values closely match experiment (fig. S34) and literature averages for poplar wood (*65*). The cellulose domains were constructed on the basis of an emerging consensus that the elementary cellulose microfibril in the plant cell wall is composed of 18 glucan chains in a 234432 arrangement (*30*, *66*–*69*). How these individual microfibrils are arranged into larger-scale macrofibrils is an active area of research, but quantitative comparison of molecular models to ssNMR results along with recent literature evidence (*19*) supports macrofibrils composed of a core cellulose bundle of roughly three to four elementary 18-chain cellulose microfibrils, with some acetylated hemicelluloses trapped between the core cellulose bundles, and the entire substructure wrapped in matrix polymers.

#### Preparation of cellulose model

The 18-chain cellulose I_β_ (*70*) model with DP of 40 in the 234432 fibril motif was constructed as an individual cellulose bundle, which exhibits surfaces of each of the (100), (200), (110), and (1-10) crystallographic planes. Different sizes of cellulose models were considered in this work, i.e., the number of elementary 18-chain cellulose microfibrils that composed the larger lignocellulose bundle varied from 4 to 10.

#### Preparation of hemicellulose model

Four different substituted xylan models with DP of 40 were constructed in this work. Experimental studies suggest that 40 to 70% of the xylose residues are acetylated on C2 or C3 positions and d-glucuronic acid groups are also attached to C2 or C3 positions in ~10% of the xylose residues (*71*). In our models, the d-glucuronic acid groups are attached to the C2 positions in every one of eight xylose residues, whereas the acetyl group is attached to the C2 position in every other xylose residue. In models where the C2 position has been occupied by a d-glucuronic acid group, the acetyl group is attached to the C3 position instead. All the glucuronic acid and acetyl substitutions are located on the even numbered xylose residues numbered from the reducing end, leading to a xylan molecule with all substitutions on the same side of the xylan backbone. Four different xylan models were constructed in this work, with the first d-glucuronic acid group located on the second, fourth, sixth, and eighth xylose residues, respectively.

#### Preparation of lignin model

A schematic model of lignin 20-mers proposed by Ralph *et al.* (*64*) for hardwood was used to construct the structure of the lignin model, which contains 13 syringyl units and 7 guaiacyl units (S:G = 65:35). Two different initial conformations of lignin 20-mers, i.e., one in a fully extended conformation, and the other one in a globular conformation, were considered in this work.

#### Assembly of whole plant SCW model

Table S2 summarizes the compositions of the SCW characterized by experiments and the PCW models used in the simulations. The xylan and lignin molecules were placed on cellulose bundles under various arrangements to generate the SCW models. Ten different initial packing scenarios were considered for the SCW complex models: (i) All the 30 xylan molecules with a twofold conformation were initially placed on the surface of the cellulose microfibril; the 58 lignin molecules were randomly placed surrounding the cellulose/xylan complex; (ii) 21 xylan molecules (70%) with a twofold conformation were placed on the surface of the cellulose microfibril; the remaining 9 xylan molecules (30%) and the 58 lignin molecules were randomly placed surrounding the cellulose/xylan complex; (iii) all the 30 xylan molecules with a twofold conformation and the 58 lignin molecules were randomly placed surrounding the cellulose bundles; (iv) eight-bundles model: 10% of xylan molecules with a twofold conformation were placed between two cellulose microfibrils, each of which contains four cellulose bundles; 60% of xylan molecules with a twofold conformation were placed on the surface of the cellulose microfibril; the remaining 30% of xylan molecules with a twofold conformation and 116 lignin molecules were randomly placed surrounding the cellulose/xylan complex; (v) 10-bundle model: 10% of xylan molecules with a twofold conformation were placed among 10 cellulose bundles; 60% of xylan molecules with a two-fold conformation were placed on the surface of the cellulose bundles; the remaining 30% of xylan molecules with twofold conformation and 145 lignin molecules were randomly placed surrounding the cellulose/xylan complex; (vi) similar to scenario b, but 30% of lignin molecules with a globular conformation were used in the initial packing; (vii) similar to scenario f, but 10% of xylan molecules were placed among the four cellulose bundles; (viii) all the 30 xylan molecules with threefold initial conformations and the 58 lignin molecules were randomly placed surrounding the four cellulose bundles; (ix) all the 30 xylan molecules with a threefold initial conformations were randomly placed surrounding the four cellulose bundles at the top half of the simulation box, whereas the 58 lignin molecules were randomly placed surrounding the four cellulose bundles at the bottom half of the simulation box; (x) all the 58 lignin molecules were randomly placed surrounding the four cellulose bundles first and then compressed. Subsequently, the 30 xylan molecules with a threefold initial conformation were randomly placed surrounding the cellulose/lignin complex to exclude any direct contacts between cellulose and xylan molecules.

#### MD simulation

The generated SCW complex models were compressed slowly by decreasing the simulation box dimensions until a desired density of 1.5 g/cm^3^ was achieved. The complex was then solvated with appropriate number of TIP3P water molecules to maintain the moisture of 3 or 6%, according to the experimental characterization (fig. S41). Sodium ions were added accordingly to maintain the total charge of each system to be neutral. The MD simulations were performed using the NAMD program (*72*), with the CHARMM36 force field for carbohydrates (*73*, *74*) and the lignin force field (*75*). The force field parameters of acetylation and glucuronic acid were generated using CHARMM General Force Field (CGenFF) program (*76*, *77*). Simulations were performed under periodic boundary conditions. Electrostatic interactions were treated with particle mesh Ewald with grid spacing of 1 Å (*78*) and short-range interactions with cutoff distance of 12 Å. The chemical bonds involving hydrogen atoms were constrained. Temperature was kept at 300 K with Langevin thermostat (*72*). The simulations were performed in NVT ensemble for 50 ns with a timestep of 1 fs for each of the 10 SCW models.

## Supplementary Materials

### This PDF file includes:

Legend for file S1 Figs. S1 to S42 Tables S1 to S9 References

## References

[1] Y. Pan, R. A. Birdsey, J. Fang, R. Houghton, P. E. Kauppi, W. A. Kurz, O. L. Phillips, A. Shvidenko, S. L. Lewis, J. G. Canadell, P. Ciais, R. B. Jackson, S. W. Pacala, A. D. McGuire, S. Piao, A. Rautiainen, S. Sitch, D. Hayes, A large and persistent carbon sink in the world's forests. Science 333, 988–993 (2011). 21764754 10.1126/science.1201609

[2] H. Zhu, W. Luo, P. N. Ciesielski, Z. Fang, J. Y. Zhu, G. Henriksson, M. E. Himmel, L. Hu, Wood-derived materials for green electronics, biological devices, and energy applications. Chem. Rev. 116, 9305–9374 (2016). 27459699 10.1021/acs.chemrev.6b00225

[3] C. Somerville, H. Youngs, C. Taylor, S. C. Davis, S. P. Long, Feedstocks for lignocellulosic biofuels. Science 329, 790–792 (2010). 20705851 10.1126/science.1189268

[4] L. Petridis, J. C. Smith, Molecular-level driving forces in lignocellulosic biomass deconstruction for bioenergy. Nat. Rev. Chem. 2, 382–389 (2018).

[5] M. E. Himmel, S. Y. Ding, D. K. Johnson, W. S. Adney, M. R. Nimlos, J. W. Brady, T. D. Foust, Biomass recalcitrance: Engineering plants and enzymes for biofuels production. Science 315, 804–807 (2007). 17289988 10.1126/science.1137016

[6] X. Zhao, L. Zhang, D. Liu, Biomass recalcitrance. Part I: The chemical compositions and physical structures affecting the enzymatic hydrolysis of lignocellulose. Biofuels Bioprod. Biorefin. 6, 465–482 (2012).

[7] C. Chen, Y. Kuang, S. Zhu, I. Burgert, T. Keplinger, A. Gong, T. Li, L. Berglund, S. J. Eichhorn, L. Hu, Structure–property–function relationships of natural and engineered wood. Nat. Rev. Mater. 5, 642–666 (2020).

[8] G. T. Beckham, J. F. Matthews, B. Peters, Y. J. Bomble, M. E. Himmel, M. F. Crowley, Molecular-level origins of biomass recalcitrance: Decrystallization free energies for four common cellulose polymorphs. J. Phys. Chem. B 115, 4118–4127 (2011). 21425804 10.1021/jp1106394

[9] P. N. Ciesielski, R. Wagner, V. S. Bharadwaj, J. Killgore, A. Mittal, G. T. Beckham, S. R. Decker, M. E. Himmel, M. F. Crowley, Nanomechanics of cellulose deformation reveal molecular defects that facilitate natural deconstruction. Proc. Natl. Acad. Sci. U.S.A. 116, 9825–9830 (2019). 31036649 10.1073/pnas.1900161116 PMC6525519

[10] K. Kulasinski, D. Derome, J. Carmeliet, Impact of hydration on the micromechanical properties of the polymer composite structure of wood investigated with atomistic simulations. J. Mech. Phys. Solids 103, 221–235 (2017).

[11] C. Zhang, M. Chen, Keten, B. Coasne, D. Derome, J. Carmeliet, Hygromechanical mechanisms of wood cell wall revealed by molecular modeling and mixture rule analysis. Sci. Adv. 7, eabi8919 (2021). 34516889 10.1126/sciadv.abi8919 PMC8442895

[12] A. McDermott, T. Polenova, *Solid State NMR Studies of Biopolymers* (Wiley, 2012), 592 pp.

[13] K. Schmidt-Rohr, H. W. Spies, *Multidimensional Solid-State NMR and Polymers* (Academic Press, 1994), 478 pp.

[14] B. Reif, S. E. Ashbrook, L. Emsley, M. Hong, Solid-state NMR spectroscopy. Nat. Rev. Methods Primers 1, 2 (2021). 34368784 10.1038/s43586-020-00002-1 PMC8341432

[15] R. Dupree, T. J. Simmons, J. C. Mortimer, D. Patel, D. Iuga, S. P. Brown, P. Dupree, Probing the molecular architecture of *Arabidopsis thaliana* secondary cell walls using two- and three-dimensional ^13^C solid state nuclear magnetic resonance spectroscopy. Biochemistry 54, 2335–2345 (2015). 25739924 10.1021/bi501552k

[16] N. J. Grantham, J. Wurman-Rodrich, O. M. Terrett, J. J. Lyczakowski, K. Stott, D. Iuga, T. J. Simmons, M. Durand-Tardif, S. P. Brown, R. Dupree, M. Busse-Wicher, P. Dupree, An even pattern of xylan substitution is critical for interaction with cellulose in plant cell walls. Nat. Plants 3, 859–865 (2017). 28993612 10.1038/s41477-017-0030-8

[17] T. J. Simmons, J. C. Mortimer, O. D. Bernardinelli, A. C. Pöppler, S. P. Brown, E. R. deAzevedo, R. Dupree, P. Dupree, Folding of xylan onto cellulose fibrils in plant cell walls revealed by solid-state NMR. Nat. Commun. 7, 13902 (2016). 28000667 10.1038/ncomms13902 PMC5187587

[18] B. Addison, D. Stengel, V. S. Bharadwaj, R. M. Happs, C. Doeppke, T. Wang, Y. J. Bomble, G. P. Holland, A. E. Harman-Ware, Selective one-dimensional ^13^C–^13^C spin-diffusion solid-state nuclear magnetic resonance methods to probe spatial arrangements in biopolymers including plant cell walls, peptides, and spider silk. J. Phys. Chem. B 124, 9870–9883 (2020). 33091304 10.1021/acs.jpcb.0c07759

[19] A. Kirui, W. Zhao, F. Deligey, H. Yang, X. Kang, F. Mentink-Vigier, T. Wang, Carbohydrate-aromatic interface and molecular architecture of lignocellulose. Nat. Commun. 13, 538 (2022). 35087039 10.1038/s41467-022-28165-3 PMC8795156

[20] O. M. Terrett, J. J. Lyczakowski, L. Yu, D. Iuga, W. T. Franks, S. P. Brown, R. Dupree, P. Dupree, Molecular architecture of softwood revealed by solid-state NMR. Nat. Commun. 10, 4978 (2019). 31673042 10.1038/s41467-019-12979-9 PMC6823442

[21] R. Cresswell, R. Dupree, S. P. Brown, C. S. Pereira, M. S. Skaf, M. Sorieul, P. Dupree, S. Hill, Importance of water in maintaining softwood secondary cell wall nanostructure. Biomacromolecules 22, 4669–4680 (2021). 34669375 10.1021/acs.biomac.1c00937 PMC8579401

[22] X. Kang, A. Kirui, M. C. Dickwella Widanage, F. Mentink-Vigier, D. J. Cosgrove, T. Wang, Lignin-polysaccharide interactions in plant secondary cell walls revealed by solid-state NMR. Nat. Commun. 10, 347 (2019). 30664653 10.1038/s41467-018-08252-0 PMC6341099

[23] Y. Gao, A. S. Lipton, Y. Wittmer, D. T. Murray, J. C. Mortimer, A grass-specific cellulose–xylan interaction dominates in sorghum secondary cell walls. Nat. Commun. 11, 6081 (2020). 33247125 10.1038/s41467-020-19837-z PMC7695714

[24] P. Duan, S. J. Kaser, J. J. Lyczakowski, P. Phyo, T. Tryfona, P. Dupree, M. Hong, Xylan structure and dynamics in native *brachypodium* grass cell walls investigated by solid-state NMR spectroscopy. ACS Omega 6, 15460–15471 (2021). 34151124 10.1021/acsomega.1c01978 PMC8210444

[25] M. Busse-Wicher, T. C. F. Gomes, T. Tryfona, N. Nikolovski, K. Stott, N. J. Grantham, D. N. Bolam, M. S. Skaf, P. Dupree, The pattern of xylan acetylation suggests xylan may interact with cellulose microfibrils as a twofold helical screw in the secondary plant cell wall of Arabidopsis thaliana. Plant J. 79, 492–506 (2014). 24889696 10.1111/tpj.12575 PMC4140553

[26] J. R. Bromley, M. Busse-Wicher, T. Tryfona, J. C. Mortimer, Z. Zhang, D. M. Brown, P. Dupree, GUX1 and GUX2 glucuronyltransferases decorate distinct domains of glucuronoxylan with different substitution patterns. Plant J. 74, 423–434 (2013). 23373848 10.1111/tpj.12135

[27] M. P. Wierzbicki, V. Maloney, E. Mizrachi, A. A. Myburg, Xylan in the middle: Understanding xylan biosynthesis and its metabolic dependencies toward improving wood fiber for industrial processing. Front. Plant Sci. 10, 176 (2019). 30858858 10.3389/fpls.2019.00176 PMC6397879

[28] C. S. Pereira, R. L. Silveira, P. Dupree, M. S. Skaf, Effects of xylan side-chain substitutions on xylan–cellulose interactions and implications for thermal pretreatment of cellulosic biomass. Biomacromolecules 18, 1311–1321 (2017). 28252951 10.1021/acs.biomac.7b00067

[29] Z. Jaafar, K. Mazeau, A. Boissière, S. le Gall, A. Villares, J. Vigouroux, N. Beury, C. Moreau, M. Lahaye, B. Cathala, Meaning of xylan acetylation on xylan-cellulose interactions: A quartz crystal microbalance with dissipation (QCM-D) and molecular dynamic study. Carbohydr. Polym. 226, 115315 (2019). 31582074 10.1016/j.carbpol.2019.115315

[30] H. Yang, J. D. Kubicki, A density functional theory study on the shape of the primary cellulose microfibril in plants: Effects of C6 exocyclic group conformation and H-bonding. Cellulose 27, 2389–2402 (2020).

[31] T. Manolikas, T. Herrmann, B. H. Meier, Protein structure determination from ^13^C spin-diffusion solid-state NMR spectroscopy. J. Am. Chem. Soc. 130, 3959–3966 (2008). 18321098 10.1021/ja078039s

[32] M. Hong, K. Schmidt-Rohr, Magic-angle-spinning NMR techniques for measuring long-range distances in biological macromolecules. Acc. Chem. Res. 46, 2154–2163 (2013). 23387532 10.1021/ar300294x PMC3714308

[33] T. Wang, J. K. Williams, K. Schmidt-Rohr, M. Hong, Relaxation-compensated difference spin diffusion NMR for detecting ^13^C–^13^C long-range correlations in proteins and polysaccharides. J. Biomol. NMR 61, 97–107 (2015). 25510834 10.1007/s10858-014-9889-0 PMC4522165

[34] R. L. Johnson, K. Schmidt-Rohr, Quantitative solid-state ^13^C NMR with signal enhancement by multiple cross polarization. J. Magn. Reson. 239, 44–49 (2014). 24374751 10.1016/j.jmr.2013.11.009

[35] L. Salmén, On the organization of hemicelluloses in the wood cell wall. Cellulose 29, 1349–1355 (2022).

[36] R. Shah, S. Huang, S. V. Pingali, D. Sawada, Y. Pu, M. Rodriguez Jr., A. J. Ragauskas, S. H. Kim, B. R. Evans, B. H. Davison, H. O’Neill, Hemicellulose–cellulose composites reveal differences in cellulose organization after dilute acid pretreatment. Biomacromolecules 20, 893–903 (2019). 30554514 10.1021/acs.biomac.8b01511

[37] L. H. Thomas, V. T. Forsyth, A. Martel, I. Grillo, C. M. Altaner, M. C. Jarvis, Structure and spacing of cellulose microfibrils in woody cell walls of dicots. Cellulose 21, 3887–3895 (2014).

[38] J. V. Vermaas, M. F. Crowley, G. T. Beckham, A quantitative molecular atlas for interactions between lignin and cellulose. ACS Sustainable Chem. Eng. 7, 19570–19583 (2019).

[39] S. Li, L. Bashline, Y. Zheng, X. Xin, S. Huang, Z. Kong, S. H. Kim, D. J. Cosgrove, Y. Gu, Cellulose synthase complexes act in a concerted fashion to synthesize highly aggregated cellulose in secondary cell walls of plants. Proc. Natl. Acad. Sci. U.S.A. 113, 11348–11353 (2016). 27647923 10.1073/pnas.1613273113 PMC5056089

[40] M. C. Jarvis, Structure of native cellulose microfibrils, the starting point for nanocellulose manufacture. Philos. Trans. A. Math. Phys. Eng. Sci. 376, 20170045 (2018). 29277742 10.1098/rsta.2017.0045

[41] A. Zitting, A. Paajanen, L. Rautkari, P. A. Penttilä, Deswelling of microfibril bundles in drying wood studied by small-angle neutron scattering and molecular dynamics. Cellulose 28, 10765–10776 (2021).

[42] K. Mazeau, C. Moine, P. Krausz, V. Gloaguen, Conformational analysis of xylan chains. Carbohydr. Res. 340, 2752–2760 (2005). 16288999 10.1016/j.carres.2005.09.023

[43] M. Busse-Wicher, A. Li, R. L. Silveira, C. S. Pereira, T. Tryfona, T. C. F. Gomes, M. S. Skaf, P. Dupree, Evolution of xylan substitution patterns in gymnosperms and angiosperms: Implications for xylan interaction with cellulose. Plant Physiol. 171, 2418–2431 (2016). 27325663 10.1104/pp.16.00539 PMC4972281

[44] I. Nieduszynski, R. H. Marchessault, Structure of β-D-(1→4′) xylan hydrate. Nature 232, 46–47 (1971). 16062821 10.1038/232046a0

[45] N. E. Thornburg, M. B. Pecha, D. G. Brandner, M. L. Reed, J. V. Vermaas, W. E. Michener, R. Katahira, T. B. Vinzant, T. D. Foust, B. S. Donohoe, Y. Román-Leshkov, P. N. Ciesielski, G. T. Beckham, Mesoscale reaction–diffusion phenomena governing lignin-first biomass fractionation. ChemSusChem 13, 4495–4509 (2020). 32246557 10.1002/cssc.202000558

[46] N. Z. Plaza, S. V. Pingali, S. Qian, W. T. Heller, J. E. Jakes, Informing the improvement of forest products durability using small angle neutron scattering. Cellulose 23, 1593–1607 (2016).

[47] J. Jakes, C. G. Hunt, S. Zelinka, P. N. Ciesielski, N. Plaza, Effects of moisture on diffusion in unmodified wood cell walls: A phenomenological polymer science approach. Forests 10, 1084 (2019).

[48] J. E. Jakes, S. L. Zelinka, C. G. Hunt, P. Ciesielski, C. R. Frihart, D. Yelle, L. Passarini, S.-C. Gleber, D. Vine, S. Vogt, Measurement of moisture-dependent ion diffusion constants in wood cell wall layers using time-lapse micro x-ray fluorescence microscopy. Sci. Rep. 10, 9919 (2020). 32555373 10.1038/s41598-020-66916-8 PMC7303177

[49] C. Liedel, Sustainable battery materials from biomass. ChemSusChem 13, 2110–2141 (2020). 32212246 10.1002/cssc.201903577 PMC7318311

[50] Y. Li, S. Yu, J. G. C. Veinot, J. Linnros, L. Berglund, I. Sychugov, Luminescent transparent wood. Opt. Mater. 5, 1600834 (2017).

[51] S. Xiao, C. Chen, Q. Xia, Y. Liu, Y. Yao, Q. Chen, M. Hartsfield, A. Brozena, K. Tu, S. J. Eichhorn, Y. Yao, J. Li, W. Gan, S. Q. Shi, V. W. Yang, M. Lo Ricco, J. Y. Zhu, I. Burgert, A. Luo, T. Li, L. Hu, Lightweight, strong, moldable wood via cell wall engineering as a sustainable structural material. Science 374, 465–471 (2021). 34672741 10.1126/science.abg9556

[52] J. Song, C. Chen, S. Zhu, M. Zhu, J. Dai, U. Ray, Y. Li, Y. Kuang, Y. Li, N. Quispe, Y. Yao, A. Gong, U. H. Leiste, H. A. Bruck, J. Y. Zhu, A. Vellore, H. Li, M. L. Minus, Z. Jia, A. Martini, T. Li, L. Hu, Processing bulk natural wood into a high-performance structural material. Nature 554, 224–228 (2018). 29420466 10.1038/nature25476

[53] P. N. Ciesielski, M. B. Pecha, A. M. Lattanzi, V. S. Bharadwaj, M. F. Crowley, L. Bu, J. V. Vermaas, K. X. Steirer, M. F. Crowley, Advances in multiscale modeling of lignocellulosic biomass. ACS Sustainable Chem. Eng. 8, 3512–3531 (2020).

[54] S. R. Decker M. Carlile, M. J. Selig, C. Doeppke, M. Davis, R. Sykes, G. Turner, A. Ziebell. Reducing the effect of variable starch levels in biomass recalcitrance screening, in *Biomass Conversion: Methods and Protocols*, M. E. Himmel, Ed. (Humana Press, 2012), pp. 181–195. 10.1007/978-1-61779-956-3_17 22843400

[55] S. Cadars, J. Sein, L. Duma, A. Lesage, T. N. Pham, J. H. Baltisberger, S. P. Brown, L. Emsley, The refocused INADEQUATE MAS NMR experiment in multiple spin-systems: Interpreting observed correlation peaks and optimising lineshapes. J. Magn. Reson. 188, 24–34 (2007). 17588789 10.1016/j.jmr.2007.05.016

[56] A. Lesage, M. Bardet, L. Emsley, Through-bond carbon−carbon connectivities in disordered solids by NMR. J. Am. Chem. Soc. 121, 10987–10993 (1999).

[57] G. Hou, S. Yan, J. Trébosc, J.-P. Amoureux, T. Polenova, Broadband homonuclear correlation spectroscopy driven by combined R2_n_^v^ sequences under fast magic angle spinning for NMR structural analysis of organic and biological solids. J. Magn. Reson. 232, 18–30 (2013). 23685715 10.1016/j.jmr.2013.04.009 PMC3703537

[58] L. Emsley, G. Bodenhausen, Gaussian pulse cascades: New analytical functions for rectangular selective inversion and in-phase excitation in NMR. Chem. Phys. Lett. 165, 469–476 (1990).

[59] K. Takegoshi, S. Nakamura, T. Terao, ^13^C–^1^H– dipolar-assisted rotational resonance in magic-angle spinning NMR. Chem. Phys. Lett. 344, 631–637 (2001).

[60] C. R. Morcombe, K. W. Zilm, Chemical shift referencing in MAS solid state NMR. J. Magn. Reson. 162, 479–486 (2003). 12810033 10.1016/s1090-7807(03)00082-x

[61] P. Sannigrahi, A. J. Ragauskas, G. A. Tuskan, Poplar as a feedstock for biofuels: A review of compositional characteristics. Biofuels Bioprod. Biorefin. 4, 209–226 (2010).

[62] M. F. Qaseem, A.-M. Wu, Balanced xylan acetylation is the key regulator of plant growth and development, and cell wall structure and for industrial utilization. Int. J. Mol. Sci. 21, 7875 (2020). 33114198 10.3390/ijms21217875 PMC7660596

[63] P. J. Smith, H.-T. Wang, W. S. York, M. J. Peña, B. R. Urbanowicz, Designer biomass for next-generation biorefineries: Leveraging recent insights into xylan structure and biosynthesis. Biotechnol. Biofuels 10, 286 (2017). 29213325 10.1186/s13068-017-0973-z PMC5708106

[64] J. Ralph, C. Lapierre, W. Boerjan, Lignin structure and its engineering. Curr. Opin. Biotechnol. 56, 240–249 (2019). 30921563 10.1016/j.copbio.2019.02.019

[65] R. M. Happs, B. Addison, C. Doeppke, B. S. Donohoe, M. F. Davis, A. E. Harman-Ware, Comparison of methodologies used to determine aromatic lignin unit ratios in lignocellulosic biomass. Biotechnol. Biofuels 14, 58 (2021). 33676549 10.1186/s13068-021-01897-y PMC7936455

[66] J. K. Polko, J. J. Kieber, The regulation of cellulose biosynthesis in plants. Plant Cell 31, 282–296 (2019). 30647077 10.1105/tpc.18.00760 PMC6447023

[67] J. L. Hill, M. B. Hammudi, M. Tien, The *Arabidopsis* cellulose synthase complex: A proposed hexamer of CESA trimers in an equimolar stoichiometry. Plant Cell 26, 4834–4842 (2014). 25490917 10.1105/tpc.114.131193 PMC4311198

[68] B. Song, S. Zhao, W. Shen, C. Collings, S.-Y. Ding, Direct measurement of plant cellulose microfibril and bundles in native cell walls. Front. Plant Sci. 11, 479 (2020). 32391038 10.3389/fpls.2020.00479 PMC7193091

[69] P. Purushotham, R. Ho, J. Zimmer, Architecture of a catalytically active homotrimeric plant cellulose synthase complex. Science 369, 1089–1094 (2020). 32646917 10.1126/science.abb2978

[70] P. Langan, N. Sukumar, Y. Nishiyama, H. Chanzy, Synchrotron x-ray structures of cellulose Iβ and regenerated cellulose II at ambient temperature and 100 K. Cellulose 12, 551–562 (2005).

[71] J. C. Walker, in *Primary Wood Processing* (Springer, 2006), pp. 23–67.

[72] J. C. Phillips, R. Braun, W. Wang, J. Gumbart, E. Tajkhorshid, E. Villa, C. Chipot, R. D. Skeel, L. Kalé, K. Schulten, Scalable molecular dynamics with NAMD. J. Comput. Chem. 26, 1781–1802 (2005). 16222654 10.1002/jcc.20289 PMC2486339

[73] E. P. Raman, O. Guvench, A. D. MacKerell Jr., CHARMM additive all-atom force field for glycosidic linkages in carbohydrates involving furanoses. J. Phys. Chem. B. 114, 12981–12994 (2010). 20845956 10.1021/jp105758h PMC2958709

[74] O. Guvench, E. Hatcher, R. M. Venable, R. W. Pastor, A. D. MacKerell Jr., CHARMM additive all-atom force field for glycosidic linkages between hexopyranoses. J. Chem. Theory Comput. 5, 2353–2370 (2009). 20161005 10.1021/ct900242e PMC2757763

[75] J. V. Vermaas, L. Petridis, J. Ralph, M. F. Crowley, G. T. Beckham, Systematic parameterization of lignin for the CHARMM force field. Green Chem. 21, 109–122 (2019).

[76] K. Vanommeslaeghe, A. D. MacKerell Jr., Automation of the CHARMM General Force Field (CGenFF) I: Bond perception and atom typing. J. Chem. Inf. Model. 52, 3144–3154 (2012). 23146088 10.1021/ci300363c PMC3528824

[77] K. Vanommeslaeghe, E. P. Raman, A. D. MacKerell Jr., Automation of the CHARMM General Force Field (CGenFF) II: Assignment of bonded parameters and partial atomic charges. J. Chem. Inf. Model. 52, 3155–3168 (2012). 23145473 10.1021/ci3003649 PMC3528813

[78] T. Darden, D. York, L. Pedersen, Particle mesh Ewald: An *N*⋅log(*N*) method for Ewald sums in large systems. J. Chem. Phys. 98, 10089–10092 (1993).

[79] A. E. Bennett, C. M. Rienstra, M. Auger, K. V. Lakshmi, R. G. Griffin, Heteronuclear decoupling in rotating solids. J. Chem. Phys. 103, 6951–6958 (1995).

