# Supplementary material for: Atomistic, macromolecular model of the Populus secondary cell wall informed by solid-state NMR
Source: Sci Adv. 2024 Jan 3;10(1):eadi7965. doi: 10.1126/sciadv.adi7965 (PMC10776008; doi:10.1126/sciadv.adi7965)
Supplement: Supplementary file 1 — Legend for file S1 Figs. S1 to S42 Tables S1 to S9 References [file sciadv.adi7965_sm.pdf]

Science Advances
AAAS

Supplementary Materials for

Atomistic, macromolecular model of the *Populus* secondary cell wall informed by solid-state NMR

Bennett Addison *et al.*

Corresponding author: Yannick J. Bomble, yannick.bomble@nrel.gov;
Peter N. Ciesielski, peter.ciesielski@nrel.gov

Sci. Adv. **10**, eadi7965 (2024)
DOI: 10.1126/sciadv.adi7965

The PDF file includes:

Legend for file S1
Figs. S1 to S42
Tables S1 to S9
References

Other Supplementary Material for this manuscript includes the following:

File S1

Supplementary File S1

A CHARMM-compatible pdb file of the model b.8 is provided as File S1. This file has identical atomic coordinates to the model to submitted to the PRB-Dev archive under accession code PDBDEV_00000215; however, Supplementary File S1 retains its original formatting as output from CHARMM to enable facile compatibility with molecular dynamics simulation software.

Spectral Deconvolution Workflow

Spectral deconvolution of 1D ^{13}C-^{13}C spin-diffusion datasets was used to quantify polymer-polymer through-space interactions on the ~1 nm length scale. Reliable peak-fitting is therefore crucial for this work. The workflow for spectral deconvolution was as follows:

1) **2D through-bond and through-space ssNMR for initial fitting parameters:**
First, peak centers and estimates for full width half max (FWHM) values were extracted from 2D ^{13}C-^{13}C correlation ssNMR datasets collected at both 600 MHz and 200 MHz field strengths (Figs S4-S6).

2) **Perform initial spectral deconvolution on polymer-selective training sub-spectra**
Then we extracted three selective sub-spectra for acetylated hemicelluloses, lignin, and cellulose using a) ~150 ppm selective 1D MultiCP-DARR-difference spectrum at short (50 ms, Fig S8) mixing time; b) 22 ppm selective 1D MultiCP-DARR-difference data at short (100 ms, Fig S9) mixing time; and c) subtracting a 3000 ms xylan-selective (22 ppm) MultiCP-DARR-difference spectrum from a non-selective 1D MultiCP-DARR spectrum (τ_m = 3000 ms) to obtain a "double-difference" plot that shows only ^{13}C sites (predominantly cellulose) that are outside ^{13}C-^{13}C spin-diffusion range from Xn Ac^{Me} sites (Fig. S10). Notably, "double-difference" spectra at mixing times from 1500 to 5000 ms are highly similar, suggesting cellulose signals can largely be fit to a profile defined by this training spectrum (Fig. S35). The three training spectra were individually phased, baseline corrected and prepared for spectral deconvolution using MestreNova version 14. Using information gathered from 2D datasets and also consulting the literature,(*19, 22*) initial peak positions and linewidths were imported into MestreNova's Line Fitting module (Global Spectral Deconvolution, GSD). All signals were first initialized using mixed (50/50) Gaussian/Lorentzian line profiles. MestreNova GSD was then asked to freely fit the training spectra. The resulting fitted parameters (peak position and widths) were evaluated for consistency with initial estimates and the literature. Grossly inconsistent signals were re-parameterized and re-fit. The process was repeated for all three training spectra (Figs. S8-10), and resulting parameters were tabulated into a peak-fitting spreadsheet accessed by a python script to perform algorithmic deconvolution on all datasets.

3) **Peak Fitting: Free fits for resolved signals, and "coached peak fitting" for unresolved signals**
For each sample, a total of 48 spectra were deconvoluted; 16 spectra for each experiment (22 ppm selection, 150 ppm selection, and non-selective quantitative reference). Spectra were pre-processed using MestreNova version 14, then exported as ascii text files. Peak fitting of all datasets was then performed algorithmically using custom python code (using lmfit module) in which the spectra were deconvoluted into Pseudo-Voight signals using the tabulated starting parameters derived from fitting the training spectra. Generally, only peak amplitudes were varied while peak positions and linewidths derived from training spectra were held constant. The code used for spectral deconvolution can be made available on request.

Resolved signals: For all resolved ^{13}C signals (e.g., Xn AcMe and AcCO, OMe, lignin aromatics), peak amplitude was allowed to vary freely while peak position and linewidths were held constant, as informed from the training spectra.

Unresolved signals: Lignin and xylan also have unresolved signals that overlap with cellulose in the 110 – 60 ppm range. One cannot confidently fit this spectral region freely due to the highly overlapped features. Therefore, for these signals we used a 'coached peak fitting' routine that required a key assumption: the spin-diffusion rate constants for lignin (source) to xylan backbone (unresolved sinks), and xylan (source) to lignin sidechain (unresolved sinks) sites are assumed identical to the lignin to xylan acetate methyl (resolved sink), or xylan to lignin methoxy (resolved sink) sites, respectively. In other words – we scaled the intensity of the unresolved sink carbons by the intensity of resolved sink carbons within the same polymer.

This "coached peak-fitting" algorithm (e.g. order of operations) was validated by visualizing the relaxation-adjusted spin-diffusion buildup behavior of inter-polymer and intra-polymer contacts (Figs. S27-S33). Namely, single-exponential buildup behavior is expected for inter-polymer spatial contacts, while buildup and decay biexponential behavior is expected for intra-polymer contacts. Incorrect coaching resulted in inconsistent buildup and decay behaviors.

The entire process was repeated for each of the 5 replicates. Fitting parameters are summarized in Table S3, which represents the average extracted fitting parameters from all replicates.

Example coached peak fitting routine: Xylan 22 ppm selection, 5000 ms mixing time

#- 1) load data, ascii format
#- 2) import initial peak positions and lineshapes.
#- 3) Fit all cellulose signals freely. All other signals locked
#- 4) Fit resolved lignin OMe, Xn AcMe, Xn AcCO only. All other signals locked
#- 5) Scale unresolved lignin and xylan signals based on resolved lignin/xylan signals (OMe, AcCO for example)
#- 6) Fit all cellulose signals freely. All other signals locked
#- 7) Fit all resolved signals (AcMe, AcCO, lignin aromatics, OMe)
#- 8) Scale unresolved lignin and xylan signals based on resolved lignin/xylan signals (OMe, AcCO for example)
#- 9) Fit all cellulose signals freely. All other signals locked
#- 10) Fit resolved lignin OMe, Xn AcMe, Xn AcCO only. All other signals locked
#- 11) Fit all cellulose signals freely. All other signals locked
#- 12) Final fit: fit all hemicellulose signals only

The exact fitting routine used for each dataset and mixing time may vary slightly from above. Example deconvoluted spectra from all 48 experiments (3 spectra, 16 spin-diffusion mixing times) are shown in Figs S11-S26. Spin-diffusion kinetics were then analyzed using either individual or summed deconvoluted peak areas.

^{13}C-^{13}C Spin-Diffusion Buildup Kinetics

During the spin-diffusion mixing period τ_m, ^{13}C magnetization exchanges with neighboring ^{13}C nuclei (T_{SD}) and also with the surrounding environment as T_1 relaxation. Since both selective (MultiCP-DARR-difference) and non-selective (MultiCP-1D DARR) datasets were collected, it was possible to isolate T_{SD} from T_1 relaxation by dividing out magnetization loss to the lattice, as previously described.(*18*) Relaxation-compensated spin-diffusion buildup curves were then fit to either single or double exponential fits (Equation S1 or Equation S2) depending on if the ^{13}C-^{13}C interaction was intra-molecular (e.g., Xn Ac^{Me} source to Xn2,3 backbone sink), or inter-molecular (e.g. Xn Ac^{Me} source to Lignin or Cellulose sinks):

1. $$\frac{\left(\frac{M_{C\,[1D-DARR\,Difference]}(t)}{M_{C\,[1D-DARR\,Difference]}(t_0)}\right)}{\left(\frac{M_{C\,[1D-DARR]}(t)}{M_{\,[1D-DARR]}(t_0)}\right)} = A\,exp\left(-\frac{t}{T_{SD}}\right) + B$$

2. $$\frac{\left(\frac{M_{C\,[1D-DARR\,Difference]}(t)}{M_{C\,[1D-DARR\,Difference]}(t_0)}\right)}{\left(\frac{M_{C\,[1D-DARR]}(t)}{M_{C\,[1D-DARR]}(t_0)}\right)} = \left[A\,exp\left(-\frac{t}{T_{SD1}}\right)\right] + \left[B\,exp\left(-\frac{t}{T_{SD2}}\right)\right] + C$$

Where $M_{c[1D\text{-}DARR\text{-}Difference]}(t)$ represents the deconvoluted signal or sum of signals of interest M for a chosen carbon(s) at mixing time $t = \tau_m$ for the selective (Xn Ac^{Me} or S3,5 / G3,4) datasets, and $M_{c[1D\text{-}DARR]}(t)$ represents the identical deconvoluted signal area for the parallel non-selective 1D-MultiCP-DARR dataset at mixing time $t = \tau_m$. Data are summarized in Tables S4 and S5 and shown in Figs. S27-S33

Supplementary Figures and Tables

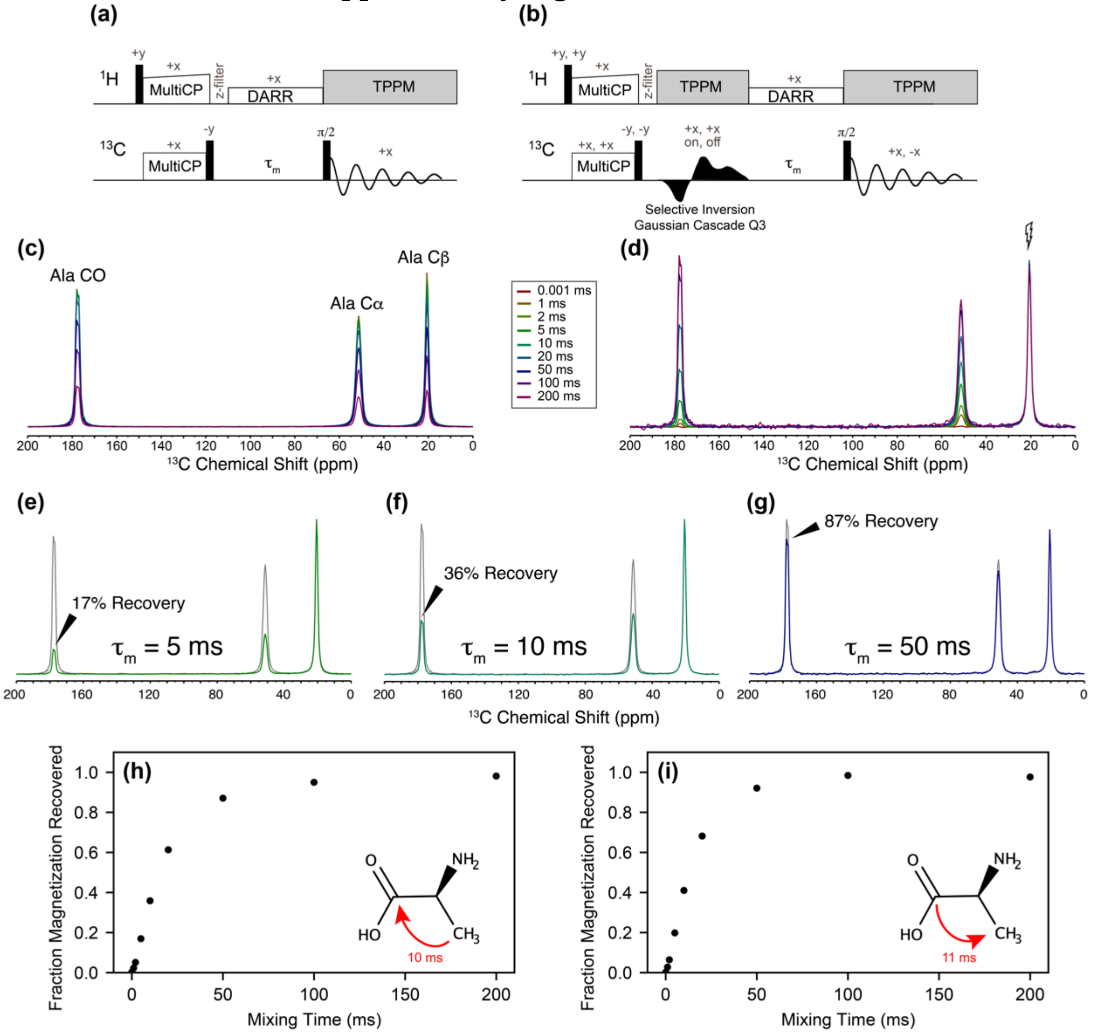

Fig. S1. MultiCP-1D-DARR pulse sequence and example methodology on ^{13}C-Alanine. (a) Non-selective MultiCP-DARR pulse sequence. Quantitative initial ^{13}C polarization is achieved using the MultiCP block,(*34*) followed by a hard ^{13}C 90 storage pulse to place all ^{13}C magnetization along +z, and a variable ^{13}C-^{13}C spin-diffusion mixing period τ_m during which dipolar coupling is reintroduced under the DARR ($\omega 1 = \omega r$) condition. A readout pulse is then applied, and high-power two-pulse phase modified (TPPM) proton decoupling is applied during acquisition.(*79*) **(b)** Selective MultiCP-DARR-Difference pulse sequence,(*18*) which is similar to the non-selective version but a 10 ms selective inversion pulse (Gaussian Cascade Q3) rotates a chosen resolved resonance to -z prior to spin diffusion.(*58*) The data is collected in an interleaved fashion (the selective inversion pulse is applied every other scan, timed with alternating receiver phase) to generate a difference spectrum so that only signal involved in ^{13}C-^{13}C spin-diffusion with the selected resonance are observed. **(c, d)** Stacked spectra from both sequences in (a) and (b) applied to [u-^{13}C]-Alanine are shown in (c) and (d), respectively. The lightning bolt signifies Ala Cβ at 21 ppm as the selected signal. Spin diffusion mixing times ranged from 0.001 to 200 ms. Non-selective spectra in (c) are shown without any normalization to highlight ^{13}C spin-lattice (T_1) relaxation, while selective spectra are normalized to the chosen Ala Cβ signal to illustrate ^{13}C-^{13}C spin-diffusion between Cβ (source) and CO and Cα sink carbons. Example stacked plots of Cβ-selected (colored) and non-selective (grey) at τ_m = 5, 10, and 50 ms, normalized to the selected signal are shown respectively in **(e, f, g)**. As indicated in each example for the CO signal, magnetization recovery is derived from the relative signal area of the sink carbons with respect to the non-selective counterpart experiment at identical mixing times. **(h, i)** Magnetization recovery plots for **(h)** Cβ (source) to CO sink and **(i)** CO (source) to Cβ (sink) shows 100% recovery by 200 ms in both directions, as expected for a uniformly labeled small crystalline compound with an inter-carbon distance of less than 0.3 nm. Spin-diffusion time constants T_{SD} extracted according to Equation S1 were also symmetric at 10-11 ms in both directions.

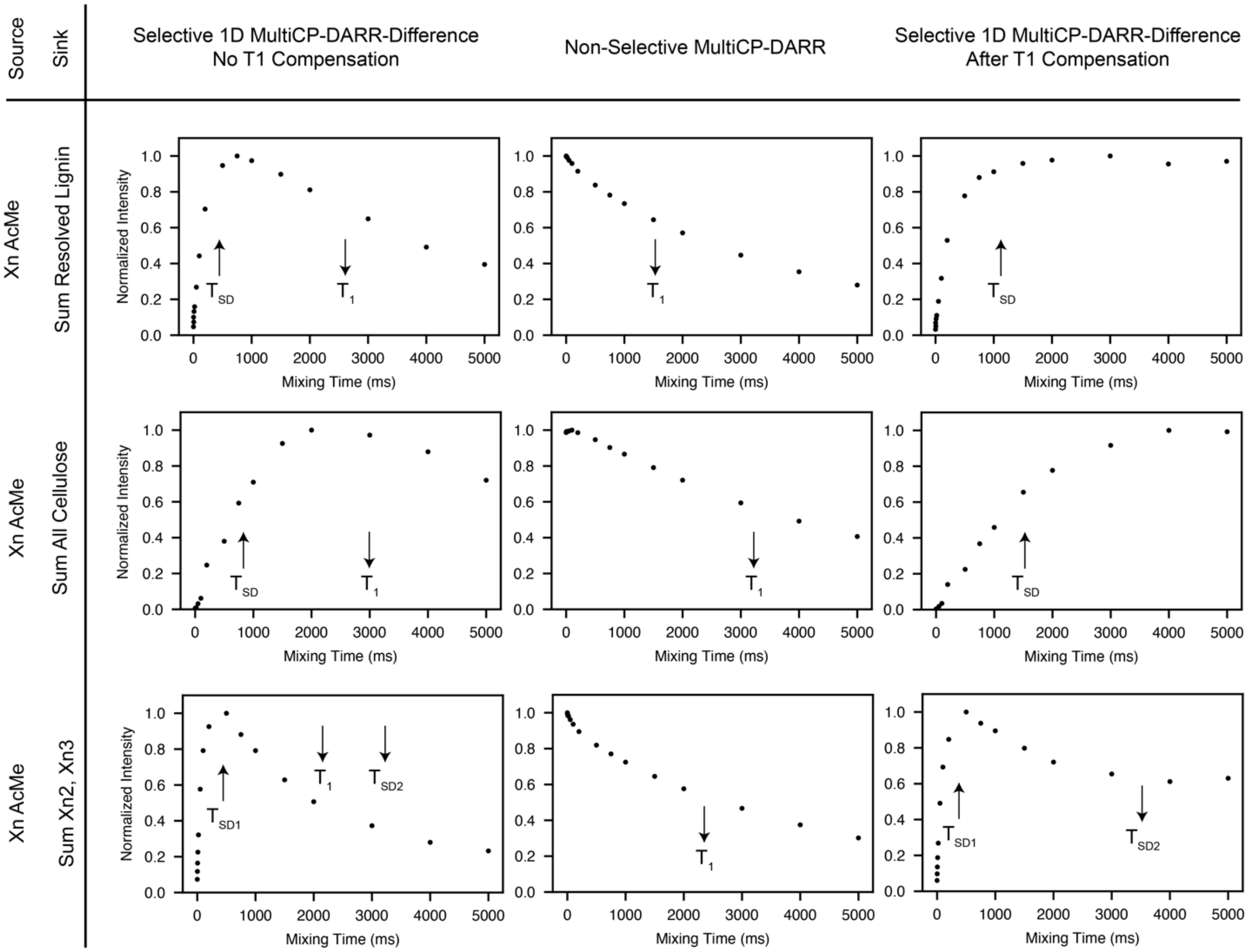

Fig. S2. Example of how T_1 compensation is achieved using parallel selective and non-selective MultiCP-1D-DARR datasets. Two datasets are collected for each replicate: Left - Selective 1D MultiCP-DARR-Difference will show magnetization transfer from the selected signal to sink carbons, but also will show signal loss to the lattice (T_1), and Middle - a parallel non-selective 1D MultiCP-DARR-Difference dataset using identical conditions and spin-diffusion mixing times. Shown here, the magnetization source is Xn AcMe at 22 ppm, and the sinks are derived from a sum of deconvoluted signals as follows: Top - Sum of resolved lignin signals including aromatics and methoxy; Middle - Sum of all cellulose signals (C1, ^{1}C4, ^{2}C4, C2/5, C3, ^{1}C6, ^{2}C6); Bottom - Sum of Xylan backbone signals at 72-75 ppm (Xn2, Xn3). Data are analyzed according to Equation S1 or S2, depending on if the interaction is intra-polymer or inter-polymer.

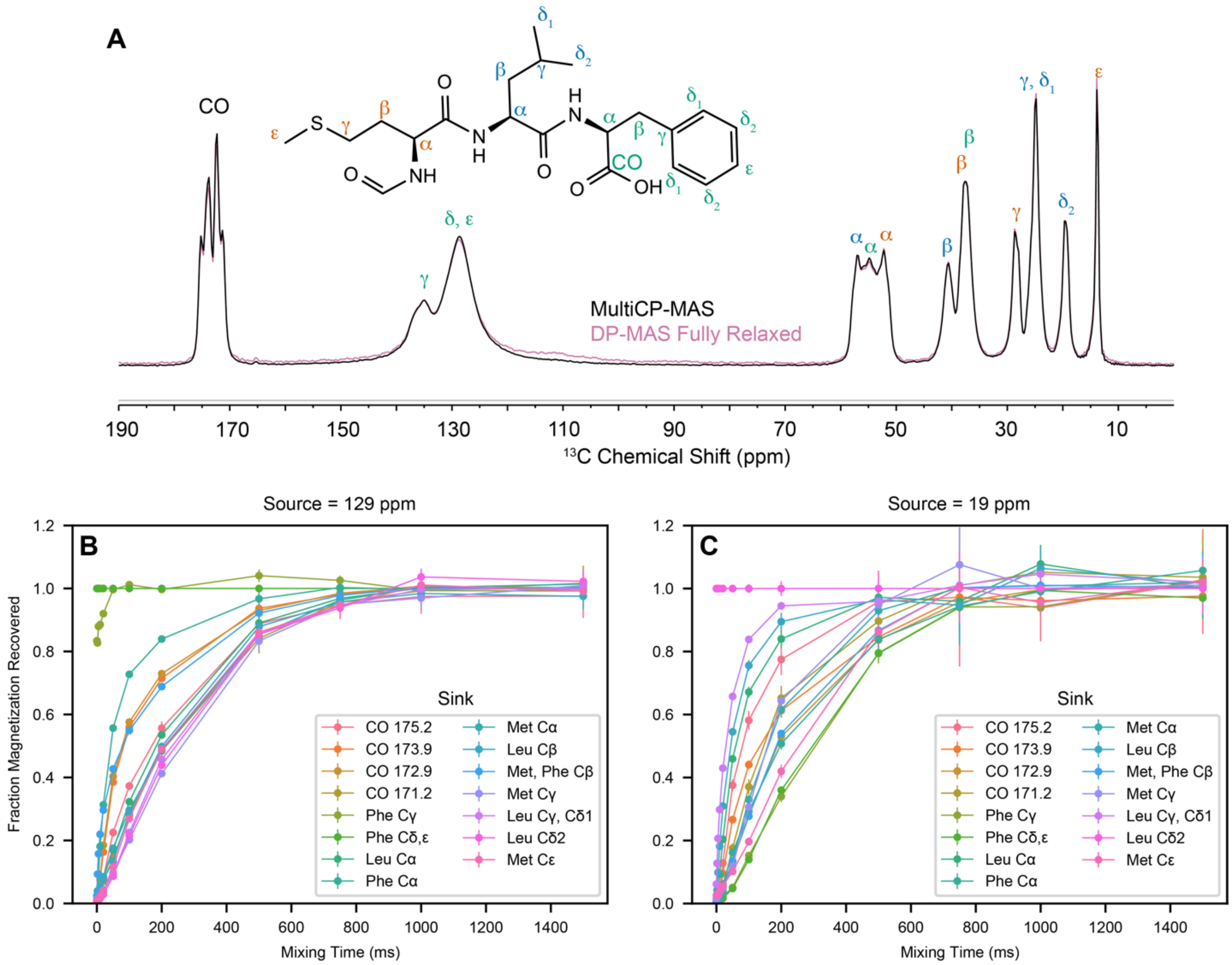

Fig. S3. MultiCP-1D-DARR on microcrystalline ^{13}C-formyl-Met-Leu-Phe (fMLF). (a) Overlay of 1D ^{13}C MultiCP-MAS and fully relaxed DP-MAS spectra of fMLF, and their assignments. (**b, c**) Magnetization recovery plots for all signals using **(b)** Phe Cδ,ε aromatic ring selection near 129 ppm, or Leu Cδ2 selection at 19 ppm. Inter-carbon distances range up to ~1 nm according to the PDB structure 1Q7O.

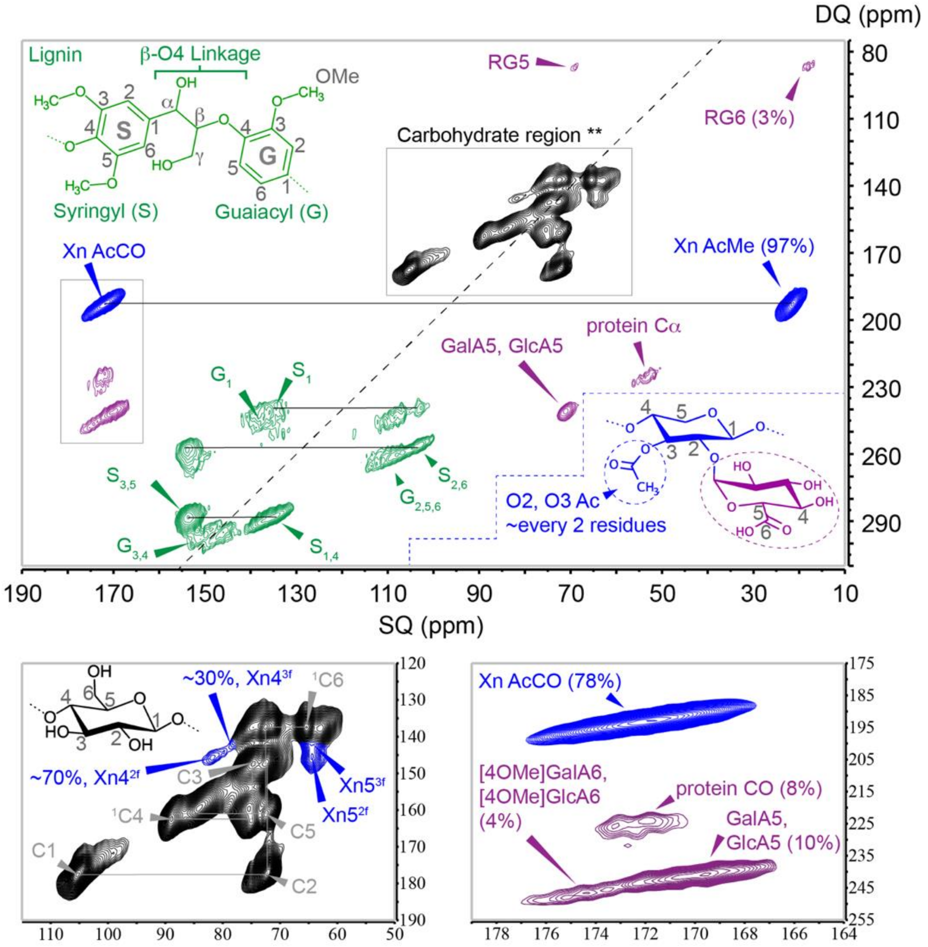

Fig. S4. 2D CP-Refocused ^{13}C-^{13}C SQ-DQ INADEQUATE data on ^{13}C-enriched poplar wood. Two-dimensional (2D) through-bond (shown here) and through-space (Fig S5, S6) ^{13}C-^{13}C solid-state NMR data were used to inform ^{13}C chemical shift assignments of the constituent polymers within poplar hardwoods in the solid state, and to compare our data to the literature. Within the neutral carbohydrate region one can easily assign cellulose signals as the dominant features. ^{13}C spin systems for both domain1 and domain2 cellulose types well resolved. Some xylan backbone signals only found after secondary wall deposition are also clearly identified. Particularly clear are the Xn4/Xn5 correlations, which show the presence of extended 2-fold (2f) xylan as the dominant type (~70% based on 2D contour integrations), and a minor component assigned to disordered 3-fold xylan (Xn4 ~78 ppm, DQ shift 142 ppm) is also identified (~30%). The carbonyl region near 170 ppm shows three main clusters at DQ frequency of 193, 225 and 240 ppm, which are assigned to acetate, structural protein, and charged carbohydrate C6 (GalA6, GlcA6) based on the chemical shifts of their neighboring carbons at 22 ppm (acetate methyl), 53 ppm (protein Cα) and ~71ppm (GalA5, GlcA5). While one cannot assume that 2D contour integrations of the SQ/DQ INAD spectrum are quantitative, they are nevertheless enlightening: roughly 80% of all rigid carbonyl carbons detected in the INAD experiment are acetate CO carbons, 8% from structural protein and 14% from negatively charged glucuronic acid (decorated xylan) or galacturonic acid (pectin). Recalling that xylan is decorated with both acetate groups every other xylose backbone unit and glucuronic acid groups every 8 units within poplar wood, the expected ratio of acetate to glucuronic acid for decorated xylan in the secondary wall is roughly in line with these integrations. This would suggest very low primary wall pectin content within the woody material. Aromatic signals from both S and G lignin subunits are also clearly identified. For example, the full ring spin system within the dominant syringyl (S) units can be traced in the 2D INADEQUATE spectrum. Lignin methoxy signals do not have a covalently attached carbon so are not observed by 2D ^{13}C-^{13}C through-bond methods, while lignin inter-unit linkage carbons expected in the 90-60 ppm range are buried beneath the dominant cellulose signals so are better assigned by 2D ^{13}C-^{13}C through-space measurement like the 2D CORD spectrum (Fig. S5). The double asterisks on the carbohydrate region indicates reduced scaling of 2D contours.

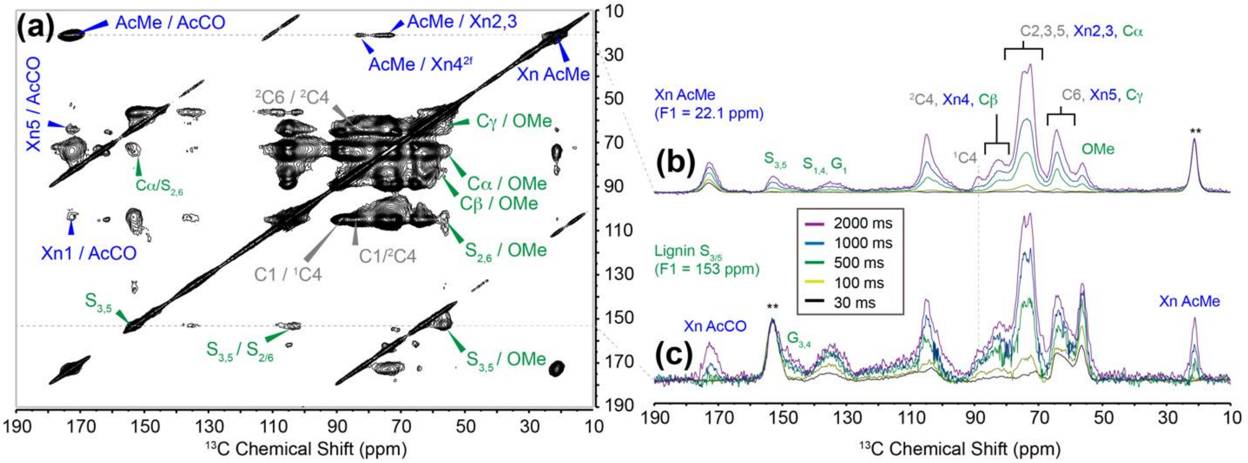

Fig. S5. 2D ^{13}C-^{13}C CORD (150 MHz ^{13}C Larmor Frequency) for assignments, and qualitative understanding of polymer contacts. (a) The short (30 ms) spin-diffusion mixing time spectrum shown in (a) reveals short range intramolecular through-space contacts, some of which are indicated with some color-coded assignments. Cellulose intramolecular correlations dominate the neutral carbohydrate region. The resolved acetate methyl and carbonyl signals show nicely resolved 2D cross-peaks with xylan backbone signals; correlations can be found for Xn1 near 104 ppm, Xn2/3 near 72-75 ppm, Xn5 predominantly near 64 ppm and the resolved Xn4 signal near 82 ppm. It's also interesting to note that resolved lignin signals, for example S3,5 at 153 ppm and lignin OMe groups at 56 ppm, show short range (30 ms) 2D cross-peaks with broad carbon signals centered near 62, 73, and 83 ppm. One might be quite tempted to assign these correlations as lignin/polysaccharide through-space interactions but considering that these cross peaks are present at short (30 ms) mixing times that generally favor intramolecular crosspeaks, and also that lignin βO4 inter-unit linkages Cγ Cα and Cβ, are known to resonate respectively at 62, 74 and 83 ppm, the most probable assignment for these cross-peaks is intramolecular lignin. ^{13}C shifts extracted from 2D methods were used to inform initial positions for spectral deconvolution. 2D CORD experiments were repeated at ^{13}C-^{13}C mixing times of 30, 100, 500, 1000 and 2000 ms. **(b, c)** Stacked 1D horizontal slices taken through the acetate methyl signal at 22 ppm **(b)** and lignin ring carbons $S_{3,5}$ at 153 ppm **(c)** are displayed for each mixing time, normalized at the intensity of the selected signal (double asterisks). Results qualitatively confirm sub-nanometer through-space contact between all three major biopolymers within poplar woody stems. Quantifying these inter-polymer interactions and estimating inter-polymer distances was achievable using selective 1D ^{13}C-^{13}C spin-diffusion methods.

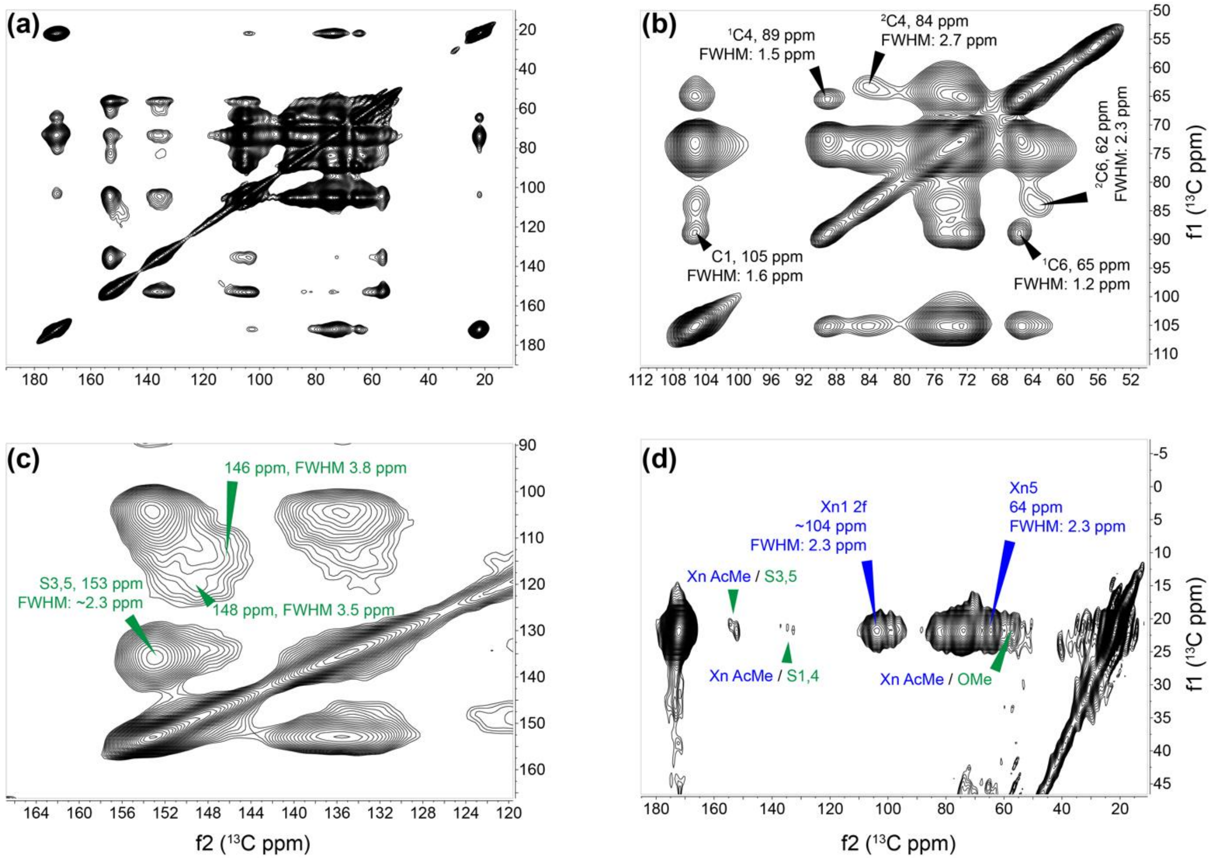

Fig. S6. Short-range (50 ms) 2D ^{13}C-^{13}C DARR (50 MHz ^{13}C Larmor Frequency) to further aid in ^{13}C chemical shift assignments and to estimate peak widths used for spectral deconvolution. Panels show differing spectral regions for clarity, including **(a)** full spectral region, **(b)** neutral carbohydrate region showing predominantly cellulose signals, **(c)** select lignin aromatic region, and **(d)** ~22 ppm region to highlight short-range correlations between xylan acetate methyl and all other carbon types. Observation of short-range correlation between Xn Ac^{Me} / lignin ring carbons from 2D data confirms our interpretation from selective 1D data. Full Width Half Max (FWHM) values are estimated for some resolved signals in the direct (f2) dimension.

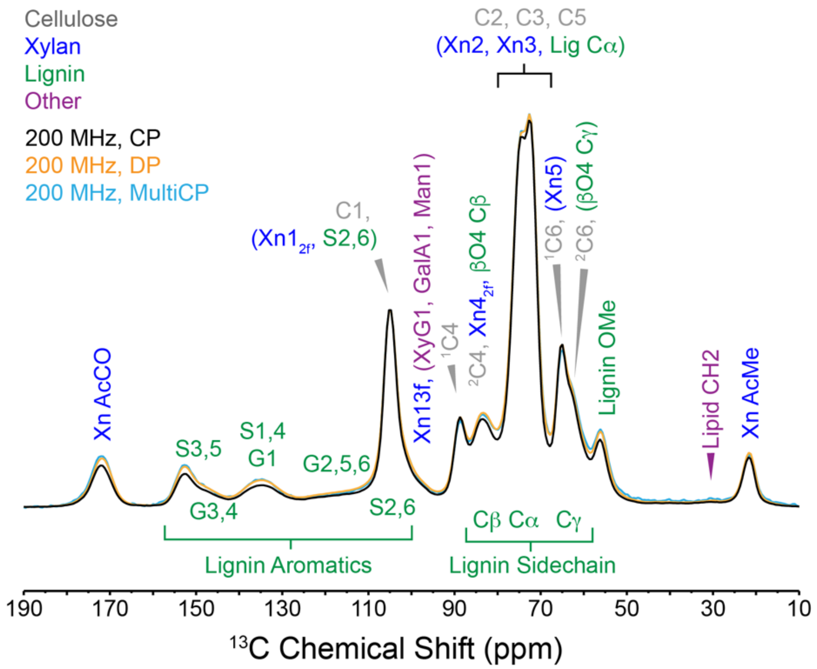

Fig. S7. Stacked 1D ^{13}C spectra at 200 MHz CP-MAS, MultiCP, and quantitative Direct Polarization (DP) data. All spectra were collected on a 4.7 Tesla NMR spectrometer and 10 kHz spinning speed (^{13}C Larmor frequency of 50 MHz). Results suggest that using the MultiCP pulse sequence yields quantitative initial ^{13}C polarization.

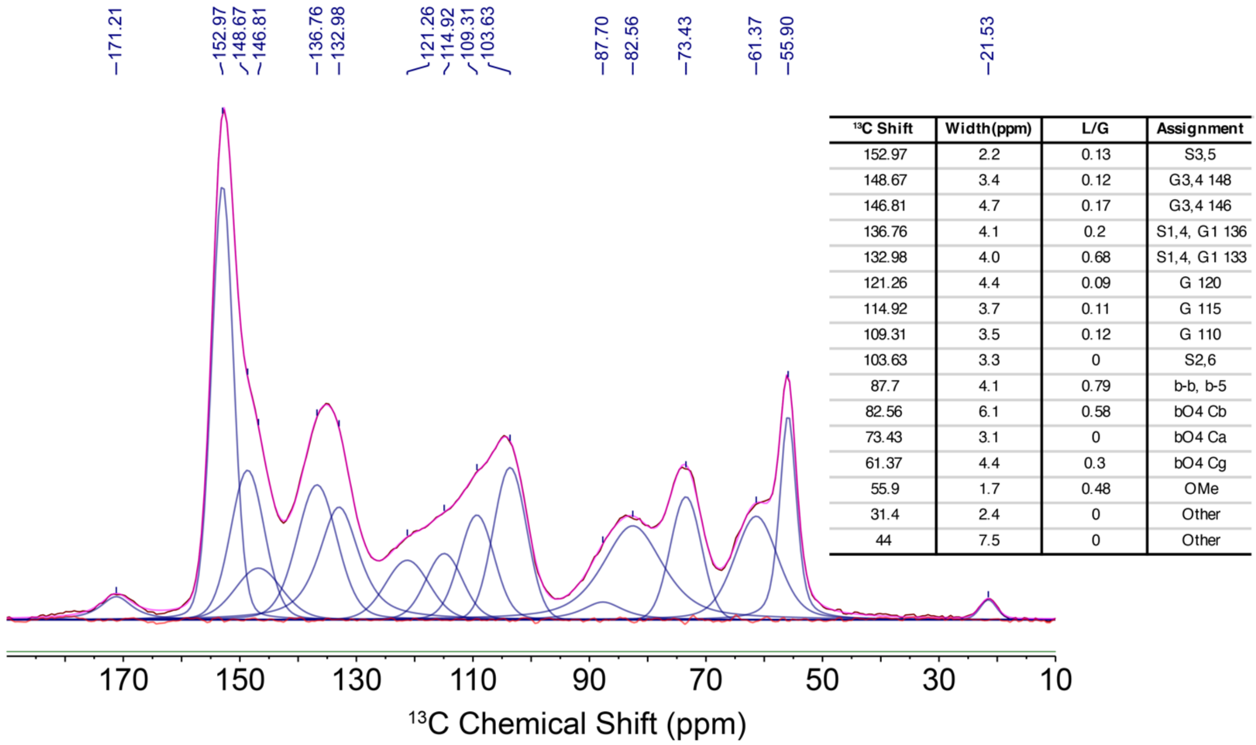

^{13}C Shift	Width(ppm)	L/G	Assignment
152.97	2.2	0.13	S3,5
148.67	3.4	0.12	G3,4 148
146.81	4.7	0.17	G3,4 146
136.76	4.1	0.2	S1,4, G1 136
132.98	4.0	0.68	S1,4, G1 133
121.26	4.4	0.09	G 120
114.92	3.7	0.11	G 115
109.31	3.5	0.12	G 110
103.63	3.3	0	S2,6
87.7	4.1	0.79	b-b, b-5
82.56	6.1	0.58	bO4 Cb
73.43	3.1	0	bO4 Ca
61.37	4.4	0.3	bO4 Cg
55.9	1.7	0.48	OMe
31.4	2.4	0	Other
44	7.5	0	Other

Fig. S8. Example deconvoluted lignin-selective (50 ms) training spectrum, obtained from the lignin-selective (S3,5 / G3,4 150 ppm selection) MultiCP-DARR-difference method with short (50 ms) ^{13}C-^{13}C spin diffusion period for a single experimental replicate. Spectral deconvolution was performed using MestreNova version 14 using the Global Spectral Deconvolution (GSD) module. Fitting parameters were initially informed from 2D ^{13}C-^{13}C correlation data. The raw data is shown as the black trace, deconvoluted signals in blue, summed resulting fit in pink, and the residuals in red. The process was repeated for all replicates. Average values from all replicates are reported in Table S3.

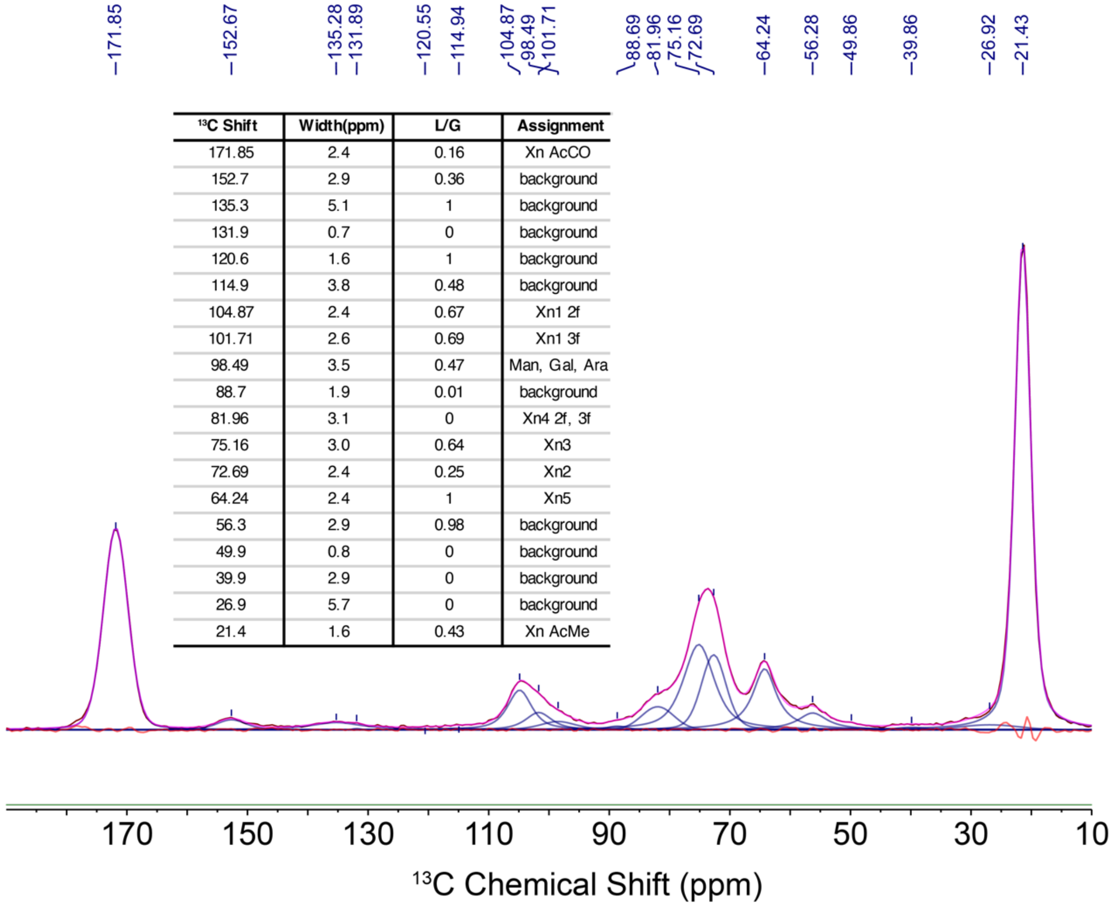

^{13}C Shift	Width(ppm)	L/G	Assignment
171.85	2.4	0.16	Xn AcCO
152.7	2.9	0.36	background
135.3	5.1	1	background
131.9	0.7	0	background
120.6	1.6	1	background
114.9	3.8	0.48	background
104.87	2.4	0.67	Xn1 2f
101.71	2.6	0.69	Xn1 3f
98.49	3.5	0.47	Man, Gal, Ara
88.7	1.9	0.01	background
81.96	3.1	0	Xn4 2f, 3f
75.16	3.0	0.64	Xn3
72.69	2.4	0.25	Xn2
64.24	2.4	1	Xn5
56.3	2.9	0.98	background
49.9	0.8	0	background
39.9	2.9	0	background
26.9	5.7	0	background
21.4	1.6	0.43	Xn AcMe

Fig. S9. Example deconvoluted xylan-selective (100 ms) training spectrum, obtained from the xylan-selective (Xn Ac^{Me}, 22 ppm selection) MultiCP-DARR-difference method with short (100 ms) ^{13}C-^{13}C spin diffusion period for a single experimental replicate. Spectral deconvolution was performed using MestreNova version 14 using the Global Spectral Deconvolution (GSD) module. Fitting parameters were initially informed from 2D ^{13}C-^{13}C correlation data. The raw data is shown as the black trace, deconvoluted signals in blue, summed resulting fit in pink, and the residuals in red. The process was repeated for all replicates. Average values from all replicates are reported in Table S3.

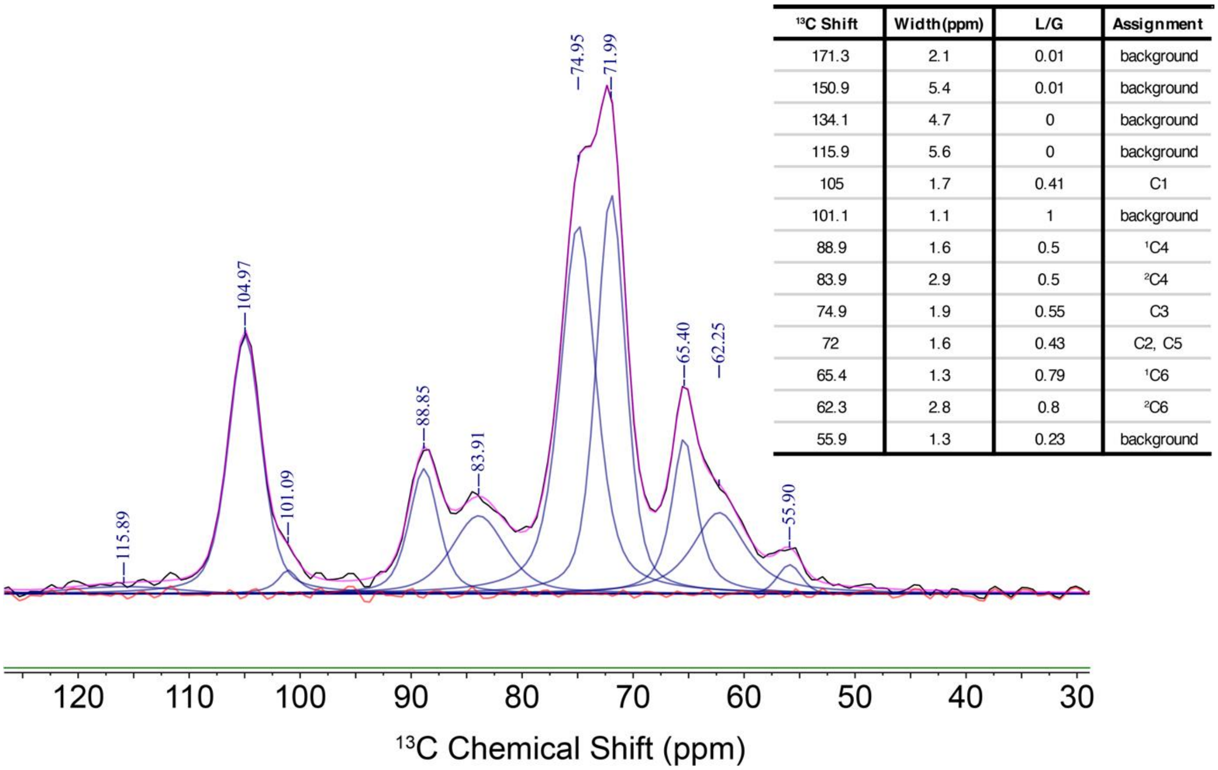

^{13}C Shift	Width(ppm)	L/G	Assignment
171.3	2.1	0.01	background
150.9	5.4	0.01	background
134.1	4.7	0	background
115.9	5.6	0	background
105	1.7	0.41	C1
101.1	1.1	1	background
88.9	1.6	0.5	^{1}C4
83.9	2.9	0.5	^{2}C4
74.9	1.9	0.55	C3
72	1.6	0.43	C2, C5
65.4	1.3	0.79	^{1}C6
62.3	2.8	0.8	^{2}C6
55.9	1.3	0.23	background

Fig. S10. Example deconvoluted selective 1D cellulose double-difference training spectrum, obtained from the 3-second double-difference (3s MultiCP-DARR minus 3s MultiCP-DARR-difference with Xn Ac^{Me} selection) for a single experimental replicate. Spectral deconvolution was performed using MestreNova version 14 using the Global Spectral Deconvolution (GSD) module. Fitting parameters were initially informed from 2D ^{13}C-^{13}C correlation data. The raw data is shown as the black trace, deconvoluted signals in blue, summed resulting fit in pink, and the residuals in red. The process was repeated for all replicates. Average values from all replicates are reported in Table S3.

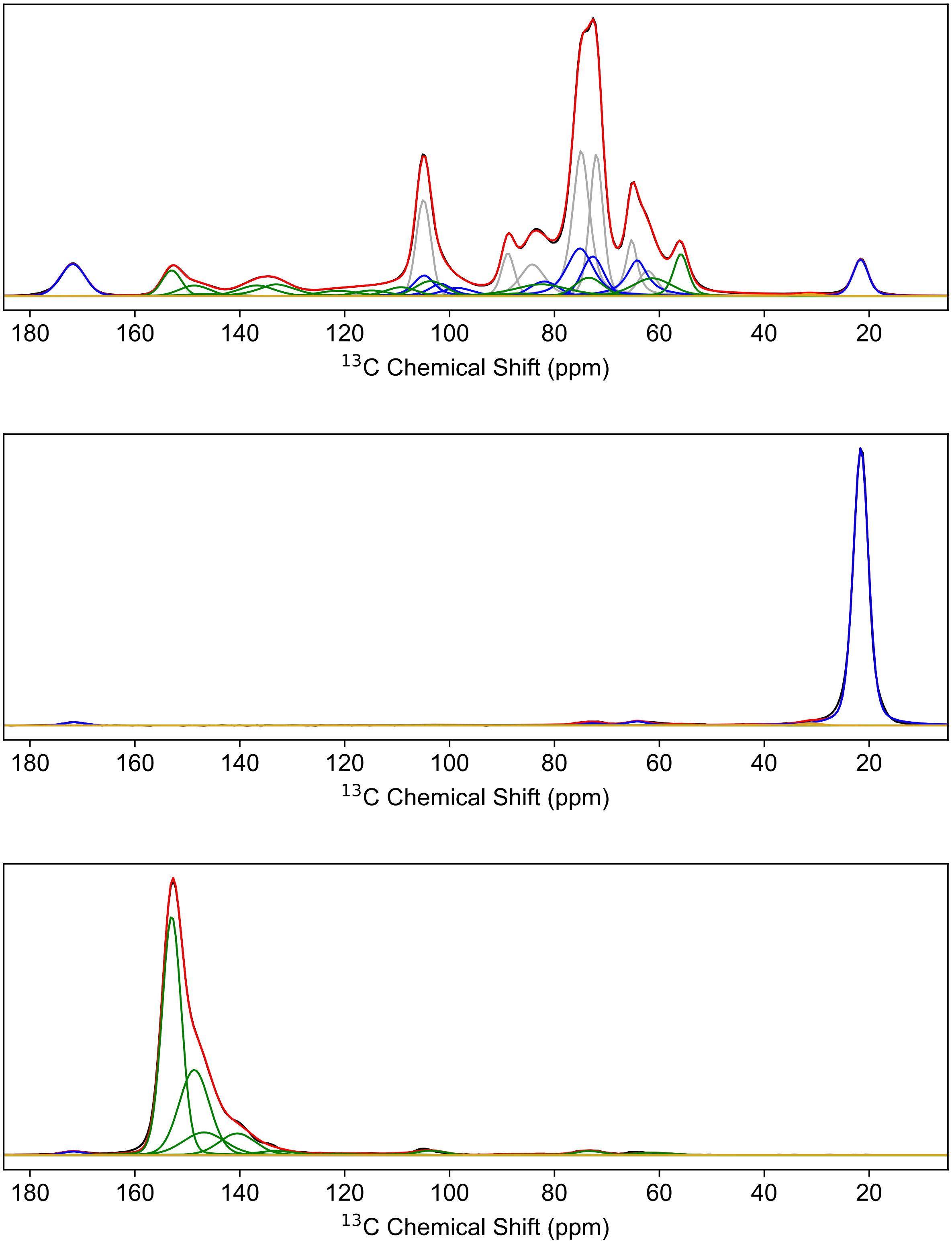

Fig. S11. 1D MultiCP-DARR Spectra, mixing time = 0.001 ms. ^{13}C MultiCP-DARR (top), MultiCP-DARR-difference at 22 ppm selection (middle) and MultiCP-DARR-difference at 150 ppm selection (bottom) using τm = 0.001 ms

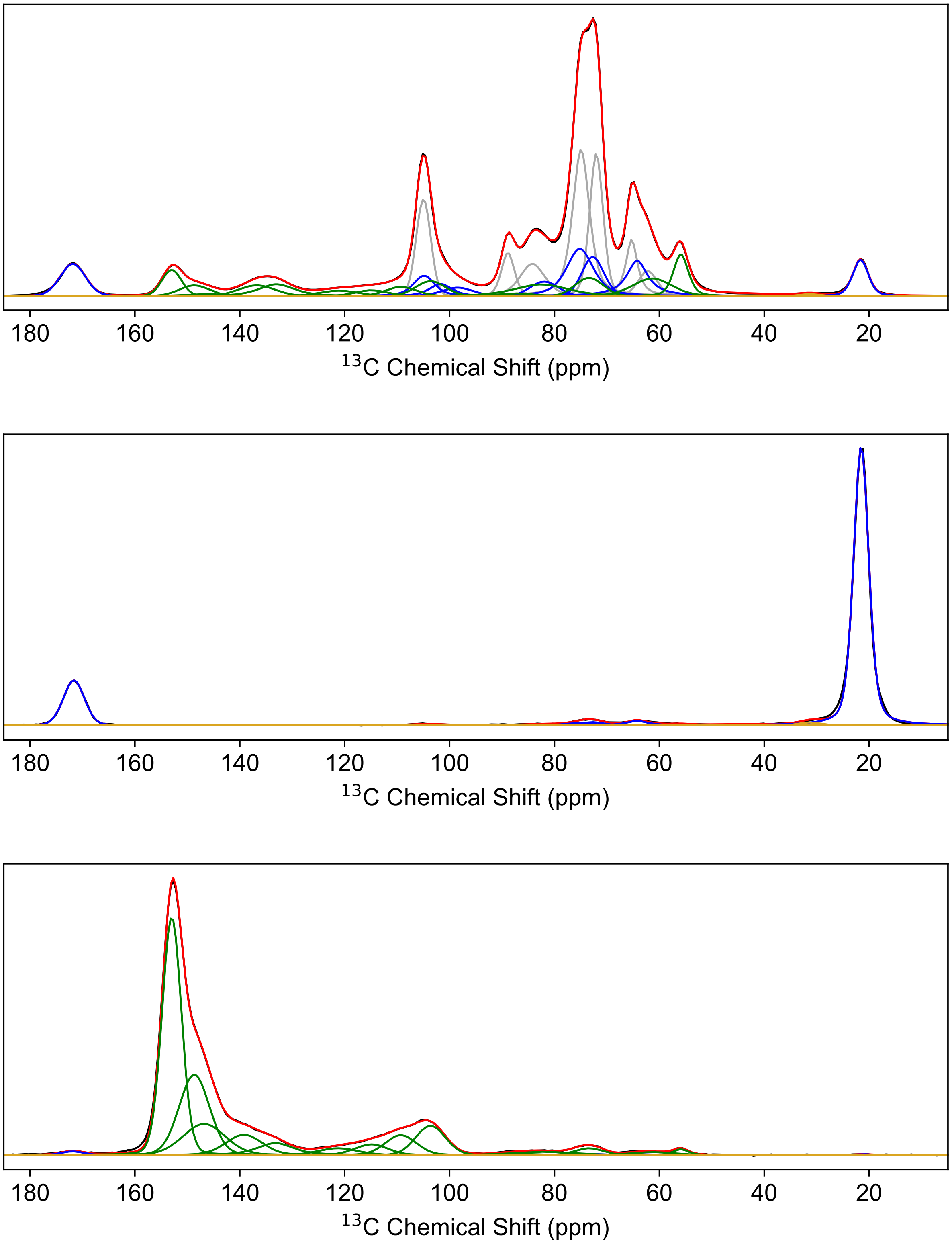

Fig. S12. 1D MultiCP-DARR Spectra, mixing time = 2 ms. ^{13}C MultiCP-DARR (top), MultiCP-DARR-difference at 22 ppm selection (middle) and MultiCP-DARR-difference at 150 ppm selection (bottom) using tm = 2 ms

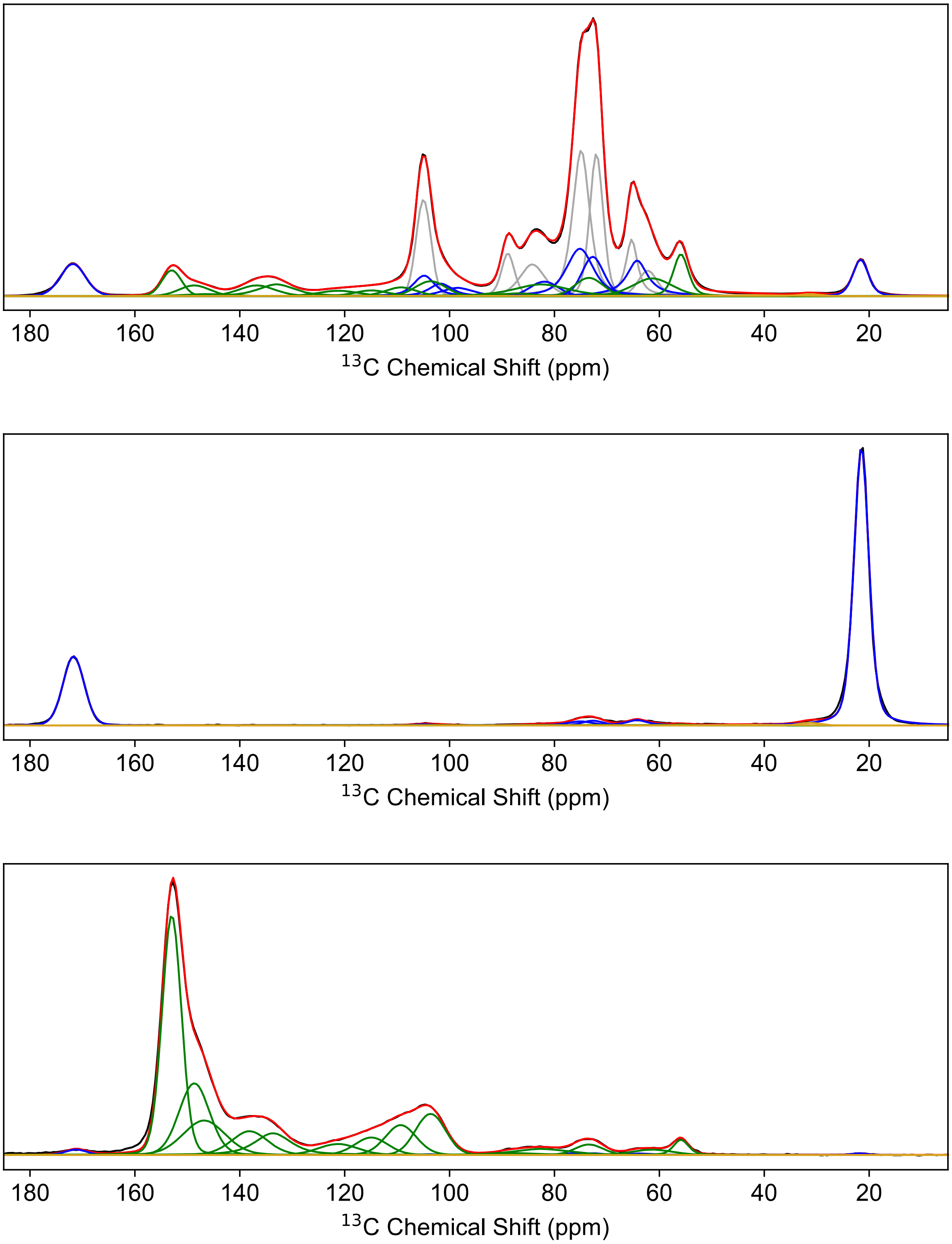

Fig. S13. 1D MultiCP-DARR Spectra, mixing time = 5 ms. ^{13}C MultiCP-DARR (top), MultiCP-DARR-difference at 22 ppm selection (middle) and MultiCP-DARR-difference at 150 ppm selection (bottom) using tm = 5 ms

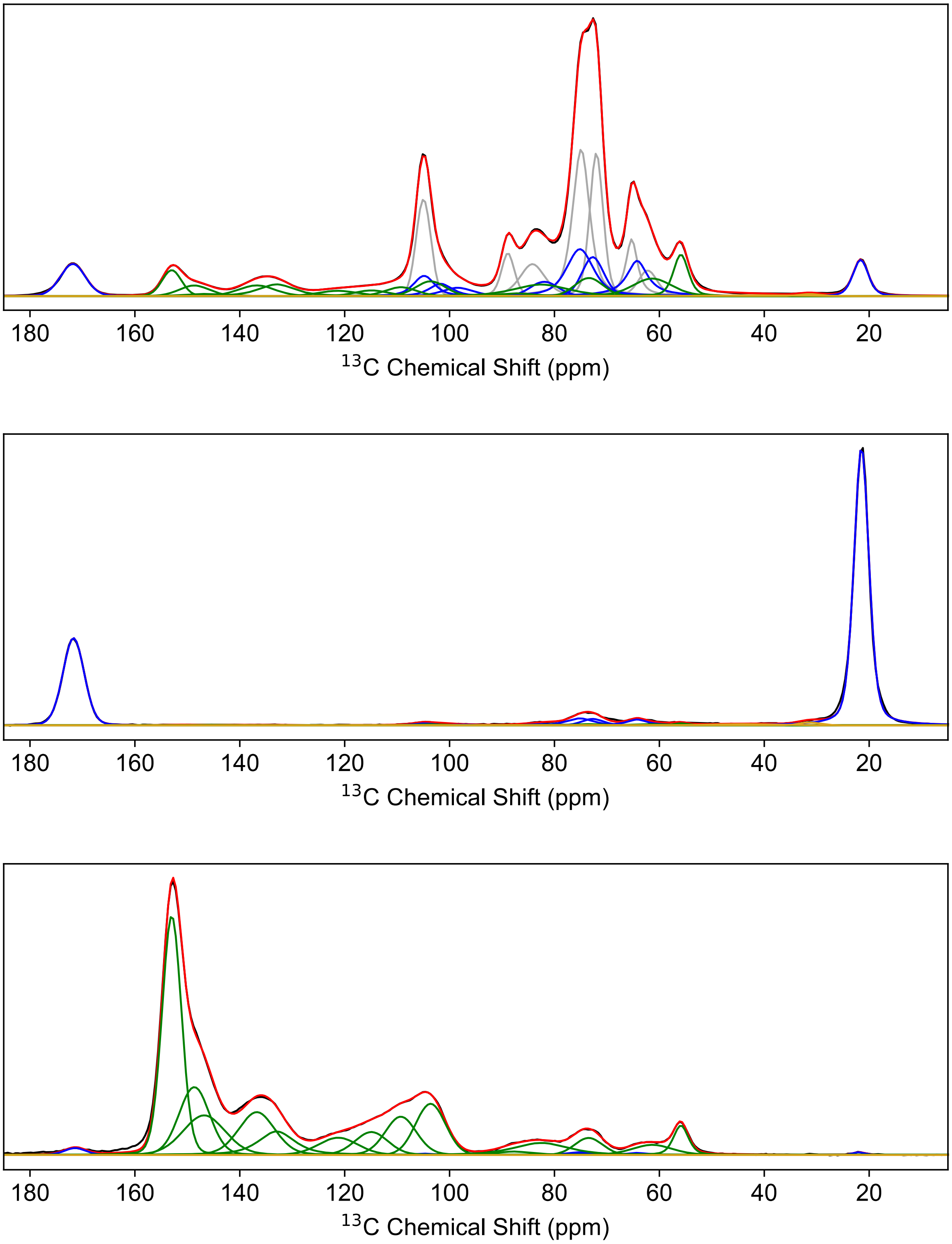

Fig. S14. 1D MultiCP-DARR Spectra, mixing time = 10 ms. ^{13}C MultiCP-DARR (top), MultiCP-DARR-difference at 22 ppm selection (middle) and MultiCP-DARR-difference at 150 ppm selection (bottom) using τm = 10 ms

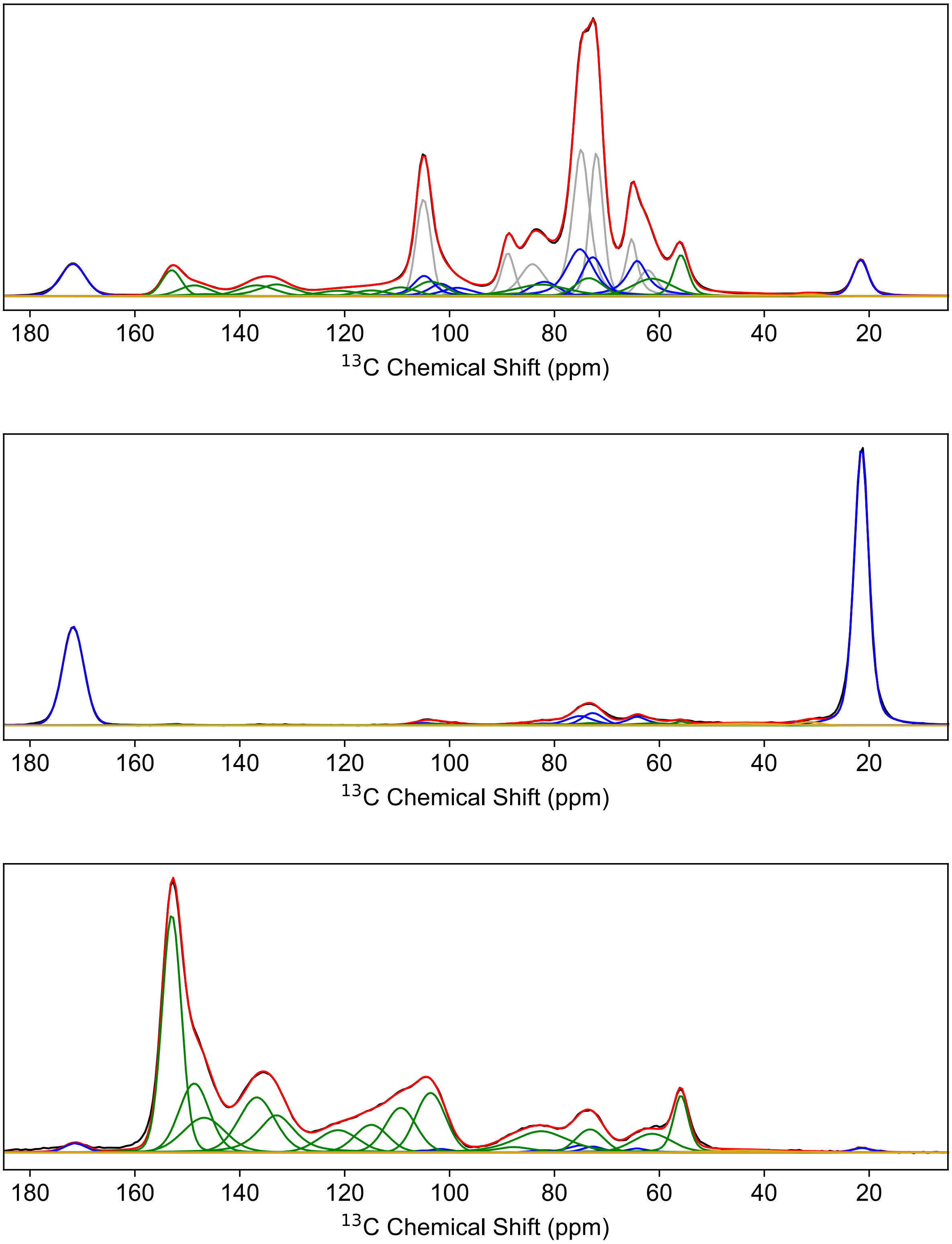

Fig. S15. 1D MultiCP-DARR Spectra, mixing time = 20 ms. ^{13}C MultiCP-DARR (top), MultiCP-DARR-difference at 22 ppm selection (middle) and MultiCP-DARR-difference at 150 ppm selection (bottom) using τm = 20 ms

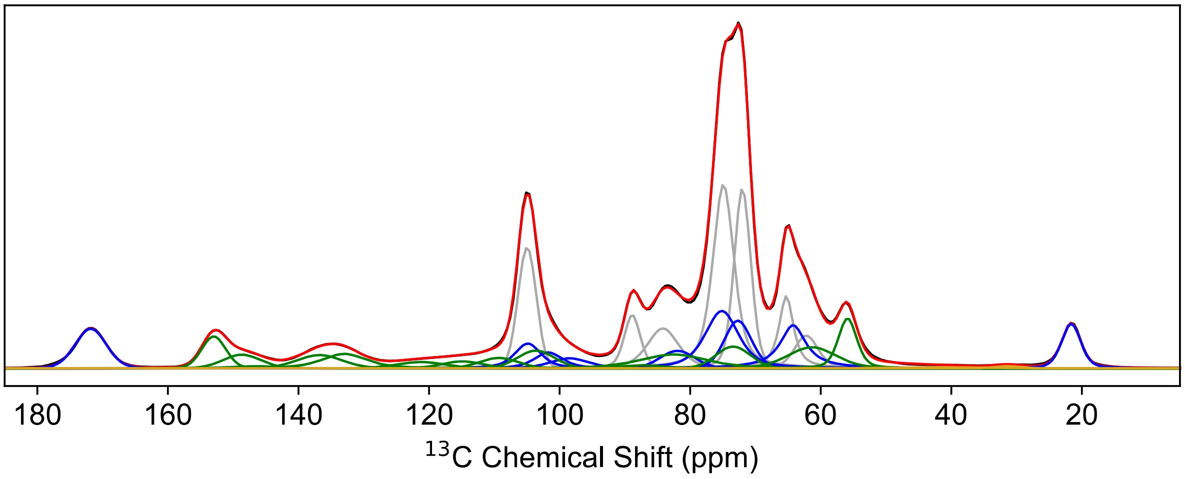

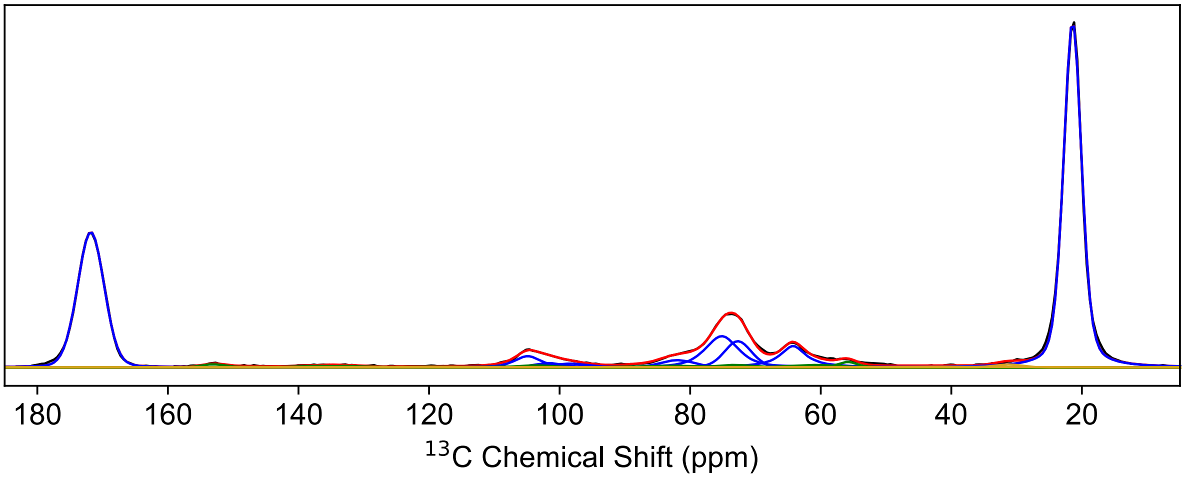

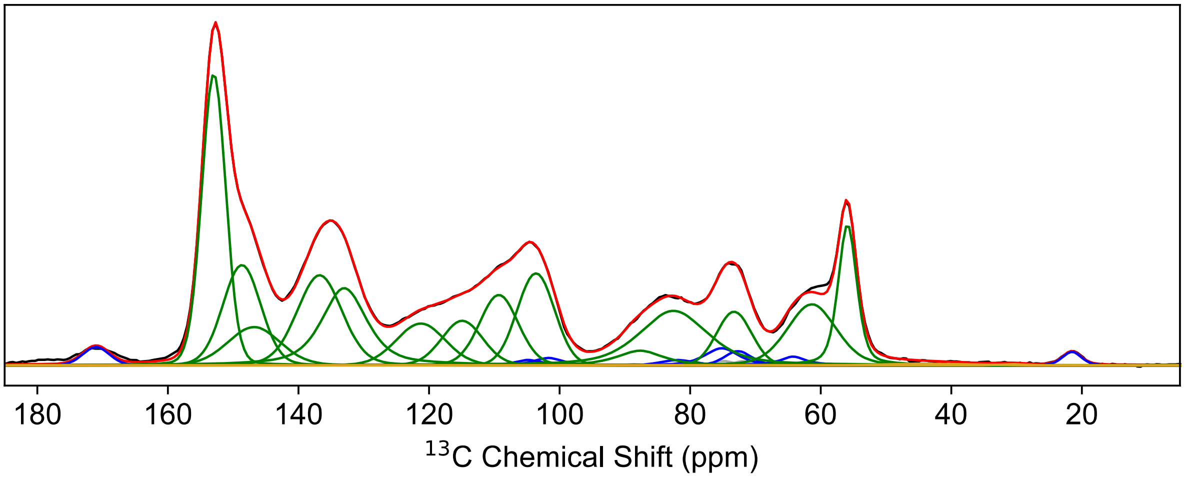

Fig. S16. 1D MultiCP-DARR Spectra, mixing time = 50 ms. ^{13}C MultiCP-DARR (top), MultiCP-DARR-difference at 22 ppm selection (middle) and MultiCP-DARR-difference at 150 ppm selection (bottom) using τm = 50 ms

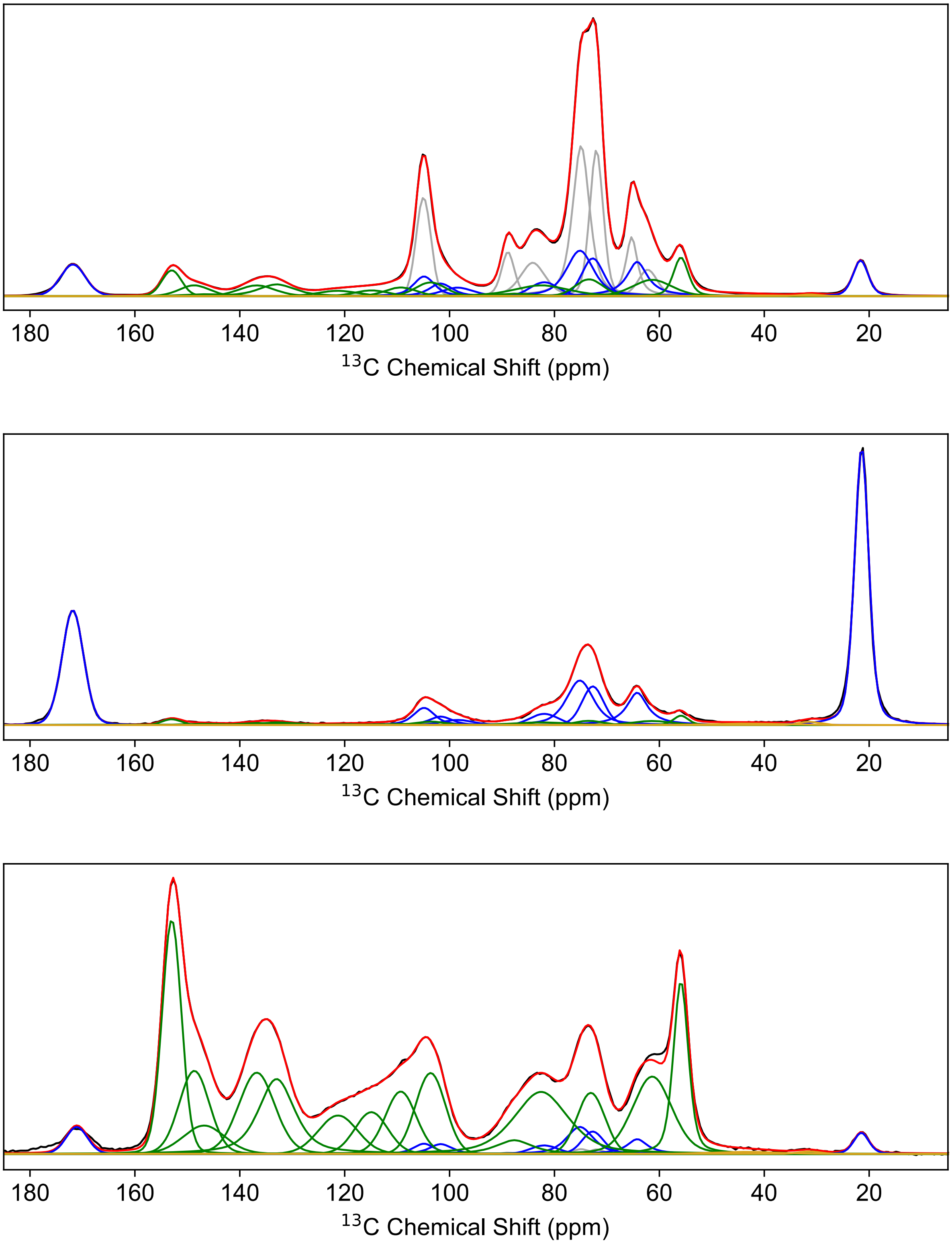

Fig. S17. 1D MultiCP-DARR Spectra, mixing time = 100 ms. ^{13}C MultiCP-DARR (top), MultiCP-DARR-difference at 22 ppm selection (middle) and MultiCP-DARR-difference at 150 ppm selection (bottom) using τm = 100 ms

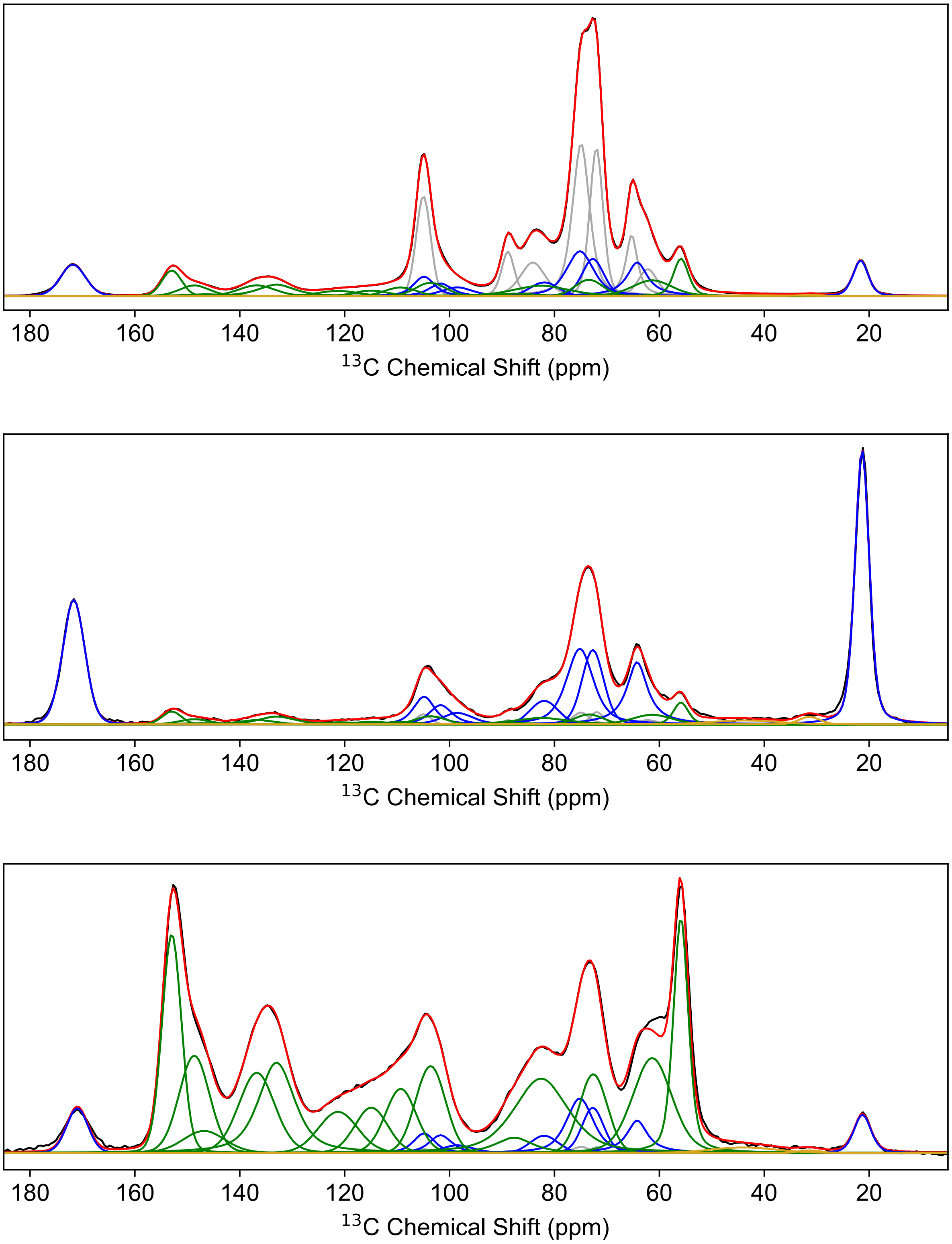

Fig. S18. 1D MultiCP-DARR Spectra, mixing time = 200 ms. ^{13}C MultiCP-DARR (top), MultiCP-DARR-difference at 22 ppm selection (middle) and MultiCP-DARR-difference at 150 ppm selection (bottom) using τm = 200 ms

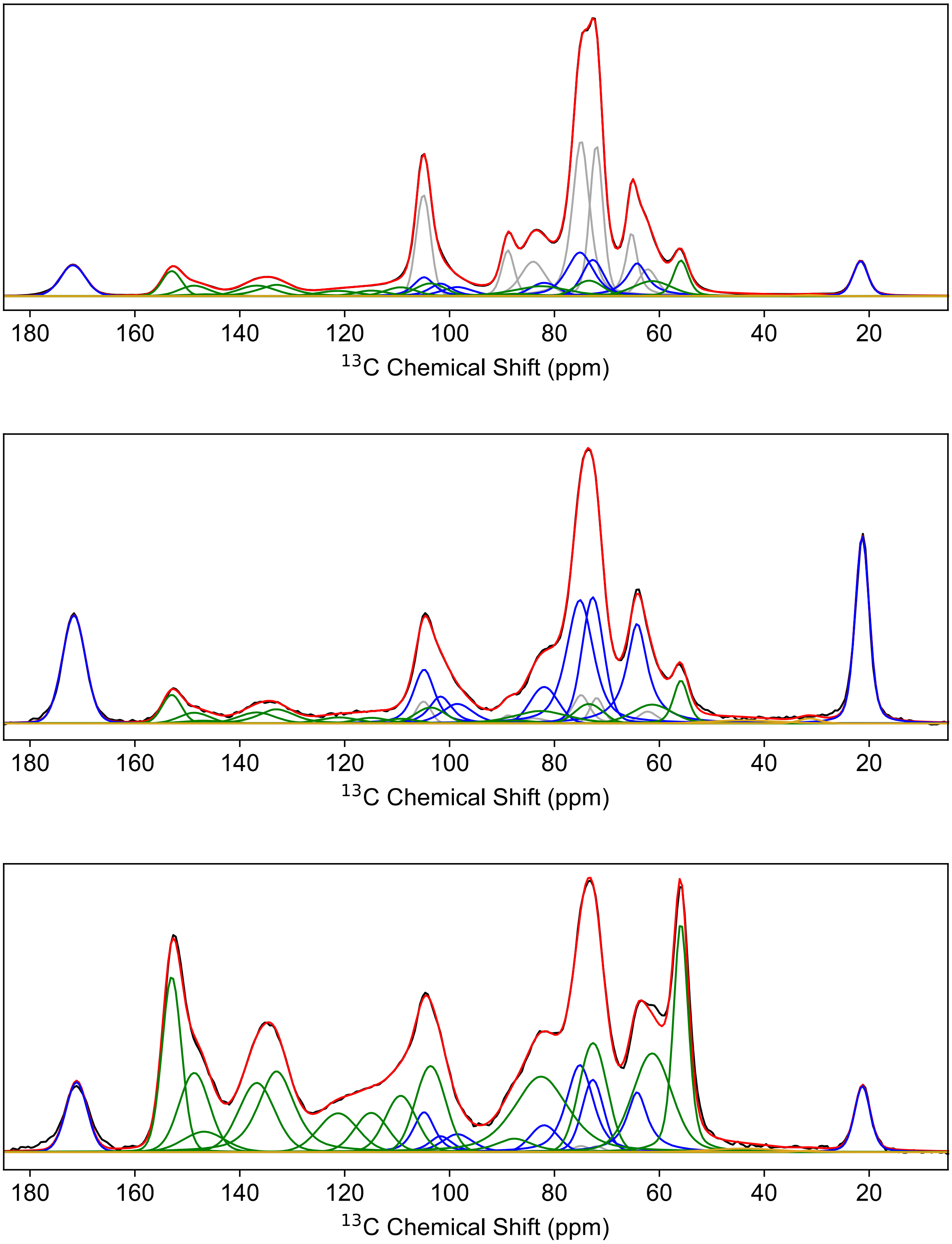

Fig. S19. 1D MultiCP-DARR Spectra, mixing time = 500 ms. ^{13}C MultiCP-DARR (top), MultiCP-DARR-difference at 22 ppm selection (middle) and MultiCP-DARR-difference at 150 ppm selection (bottom) using τm = 500 ms

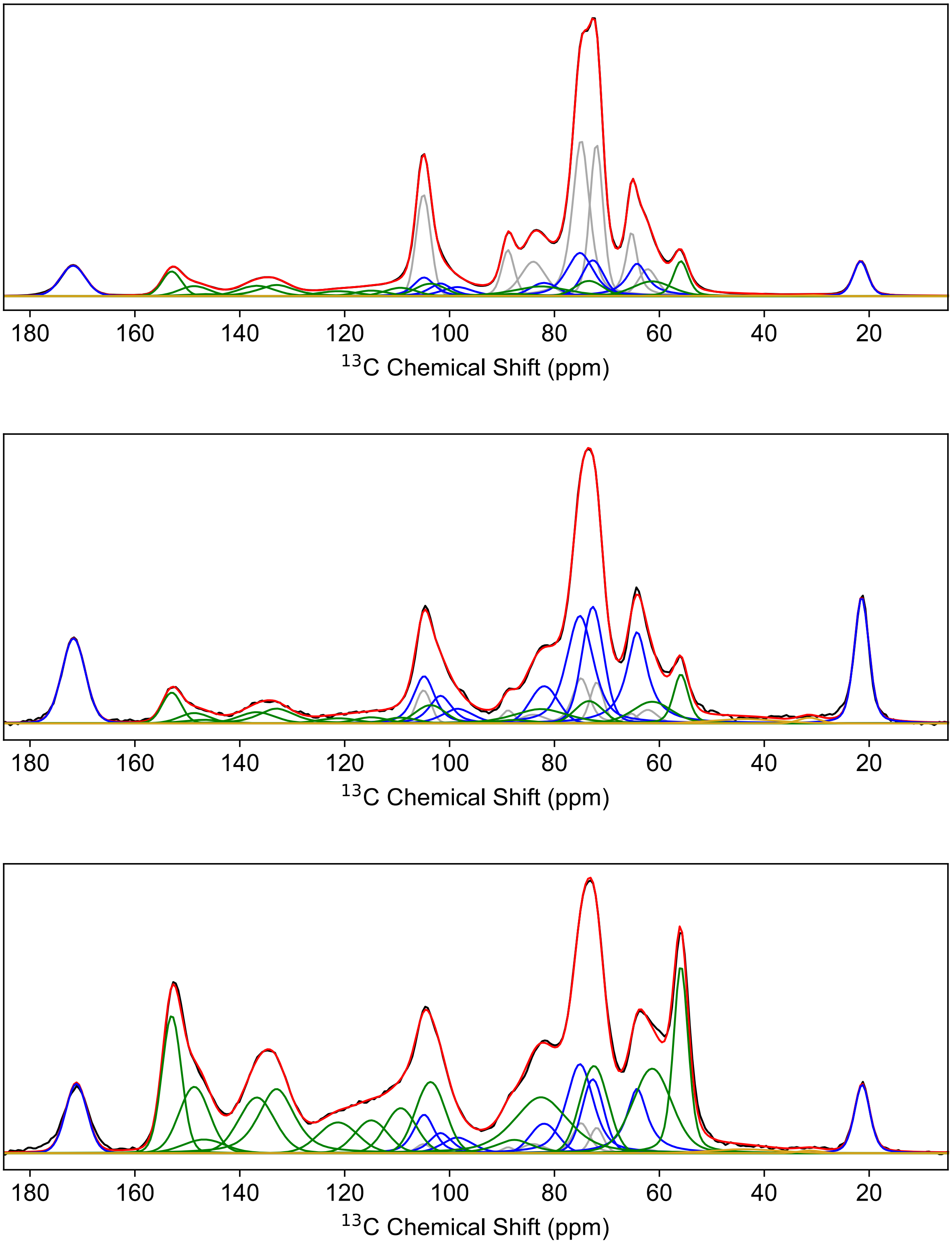

Fig. S20. 1D MultiCP-DARR Spectra, mixing time = 750 ms. ^{13}C MultiCP-DARR (top), MultiCP-DARR-difference at 22 ppm selection (middle) and MultiCP-DARR-difference at 150 ppm selection (bottom) using τm = 750 ms

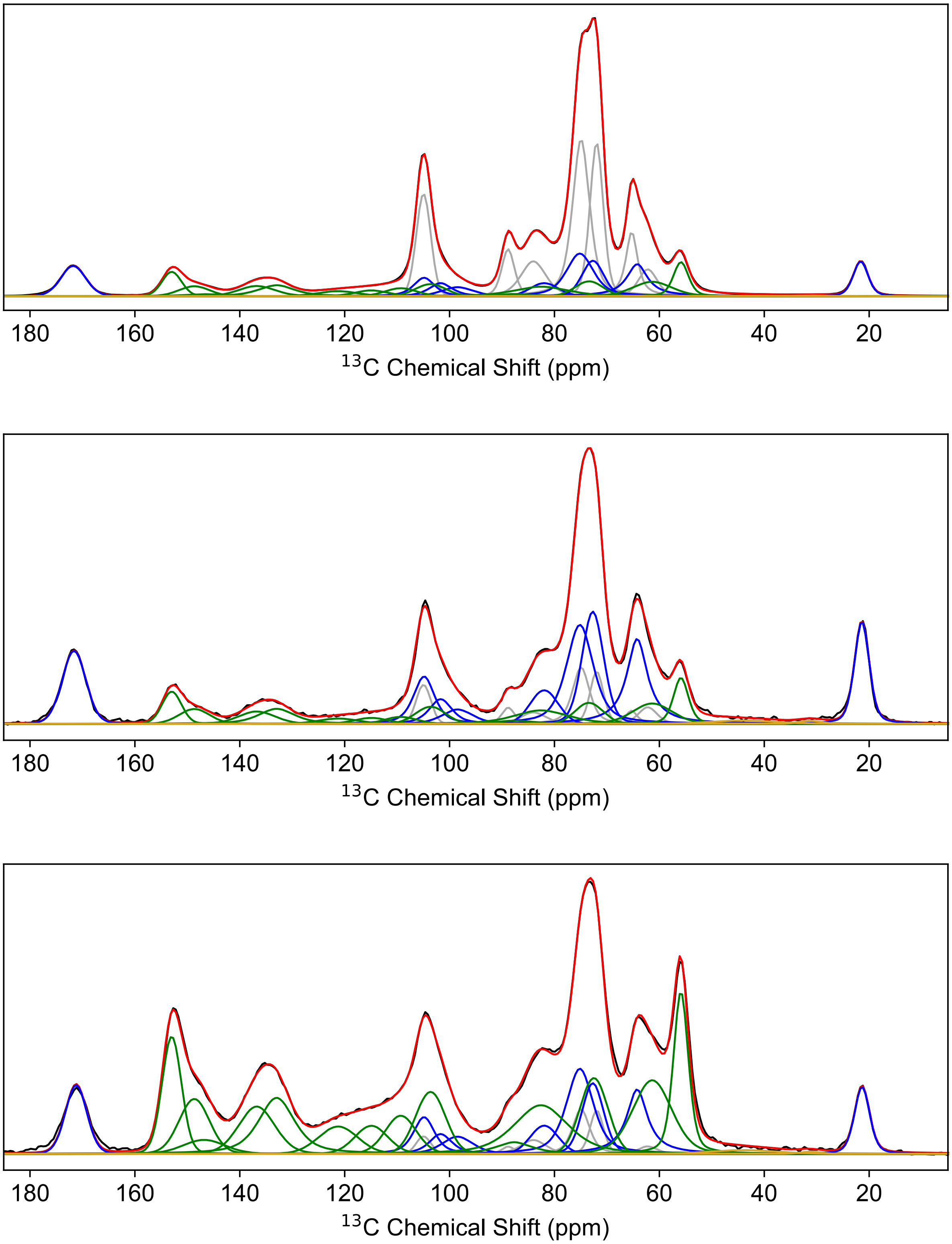

Fig. S21. 1D MultiCP-DARR Spectra, mixing time = 1000 ms. ^{13}C MultiCP-DARR (top), MultiCP-DARR-difference at 22 ppm selection (middle) and MultiCP-DARR-difference at 150 ppm selection (bottom) using τm = 1000 ms

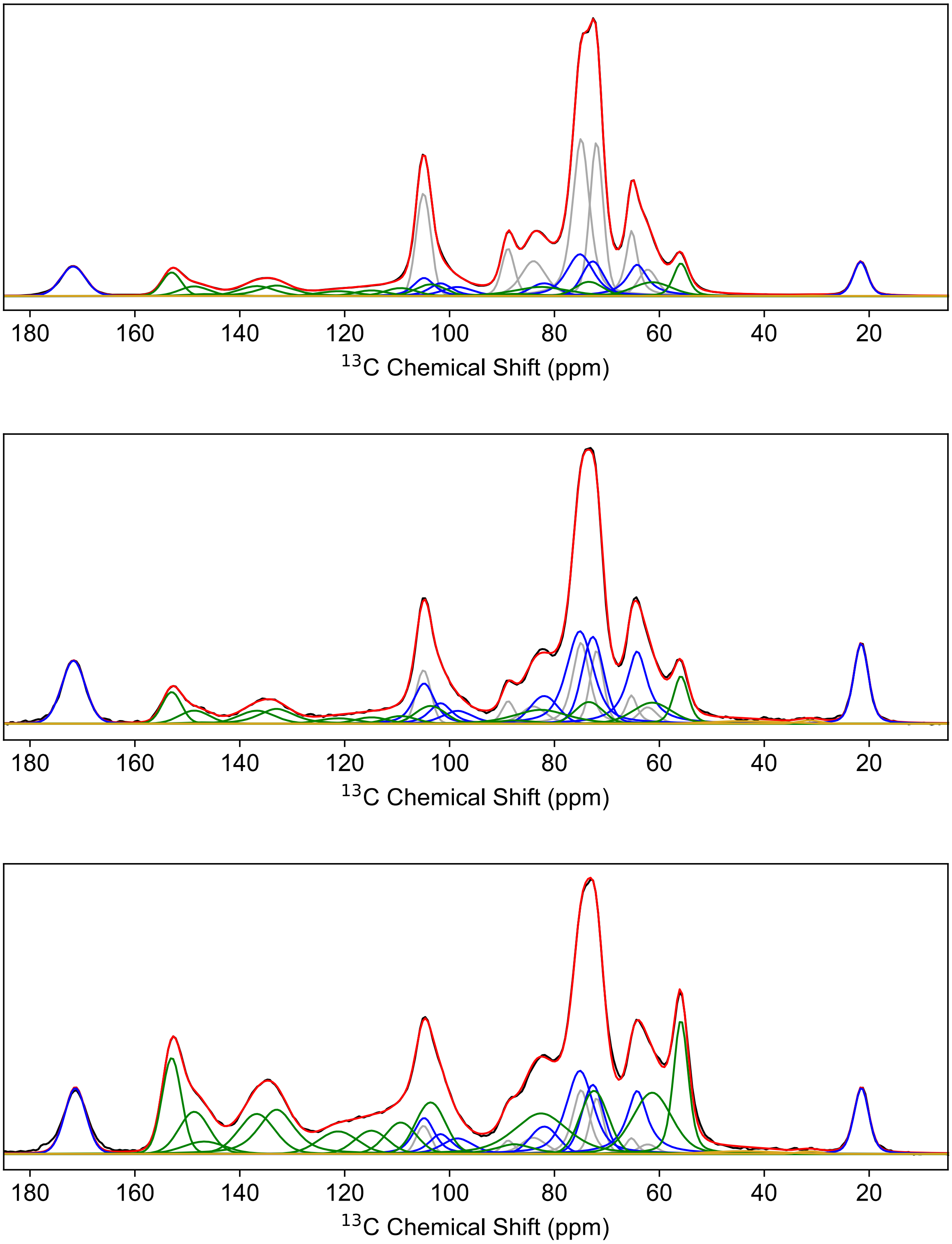

Fig. S22. 1D MultiCP-DARR Spectra, mixing time = 1500 ms. ^{13}C MultiCP-DARR (top), MultiCP-DARR-difference at 22 ppm selection (middle) and MultiCP-DARR-difference at 150 ppm selection (bottom) using τm = 1500 ms

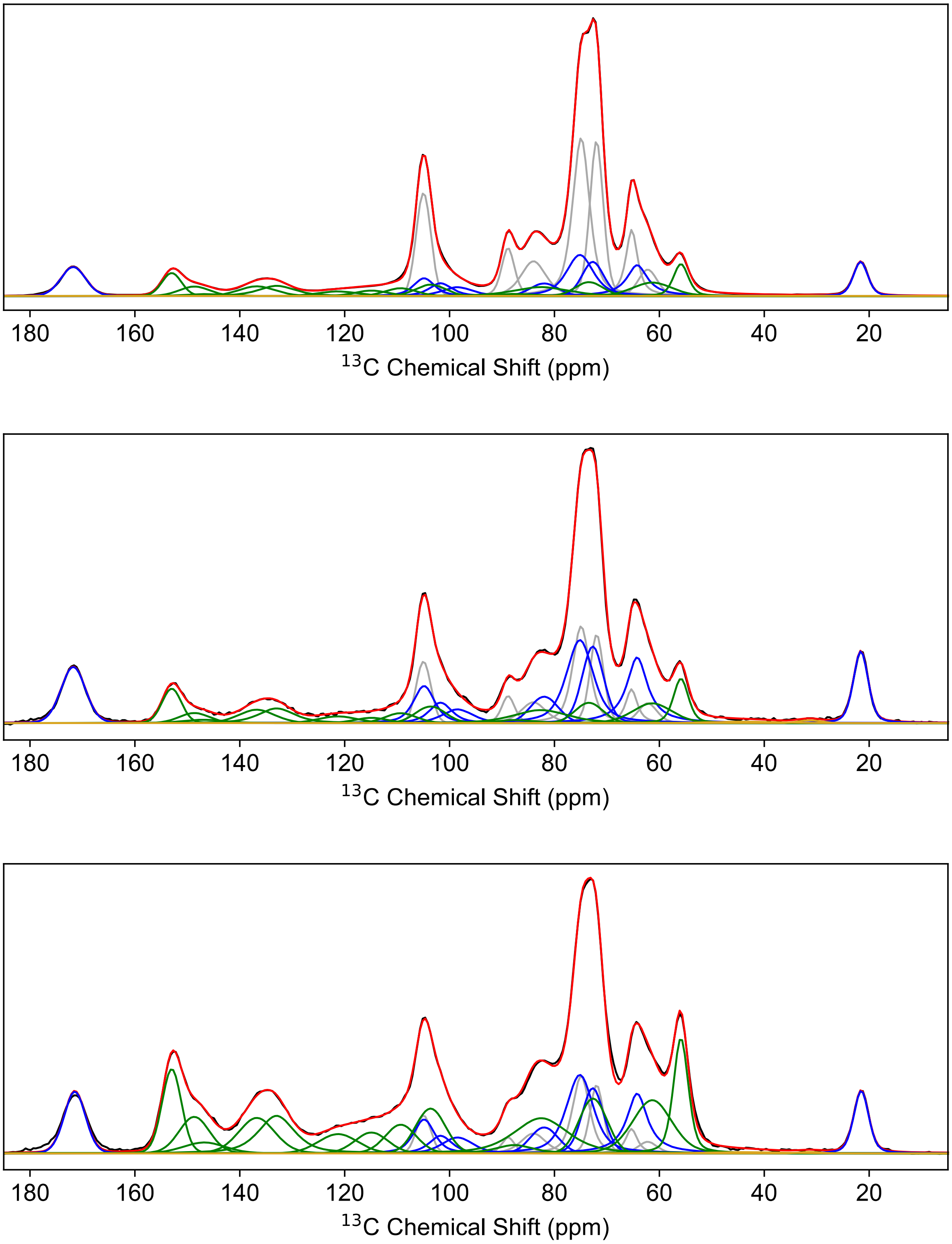

Fig. S23. 1D MultiCP-DARR Spectra, mixing time = 2000 ms. ^{13}C MultiCP-DARR (top), MultiCP-DARR-difference at 22 ppm selection (middle) and MultiCP-DARR-difference at 150 ppm selection (bottom) using τm = 2000 ms

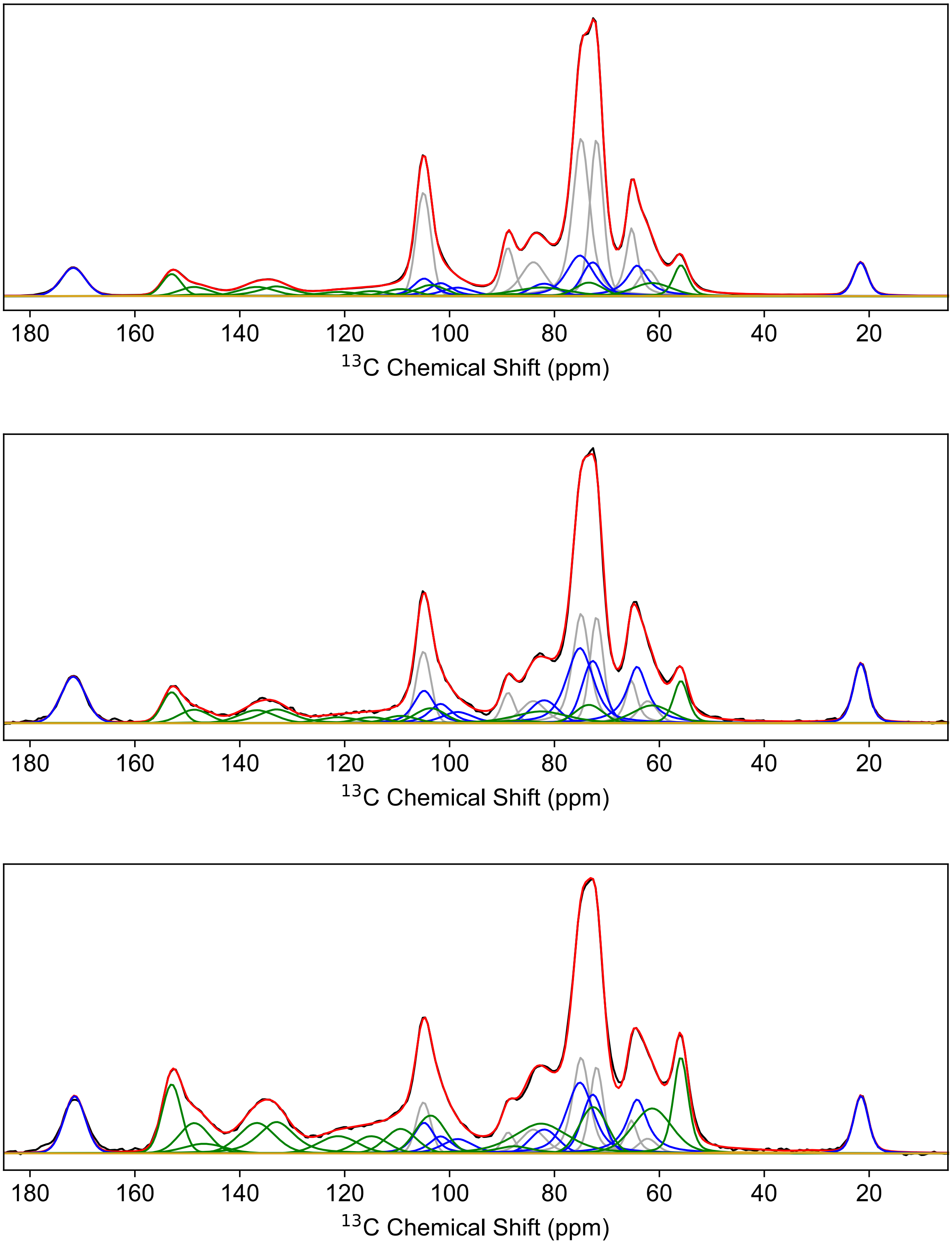

Fig. S24. 1D MultiCP-DARR Spectra, mixing time = 3000 ms. ^{13}C MultiCP-DARR (top), MultiCP-DARR-difference at 22 ppm selection (middle) and MultiCP-DARR-difference at 150 ppm selection (bottom) using τm = 3000 ms

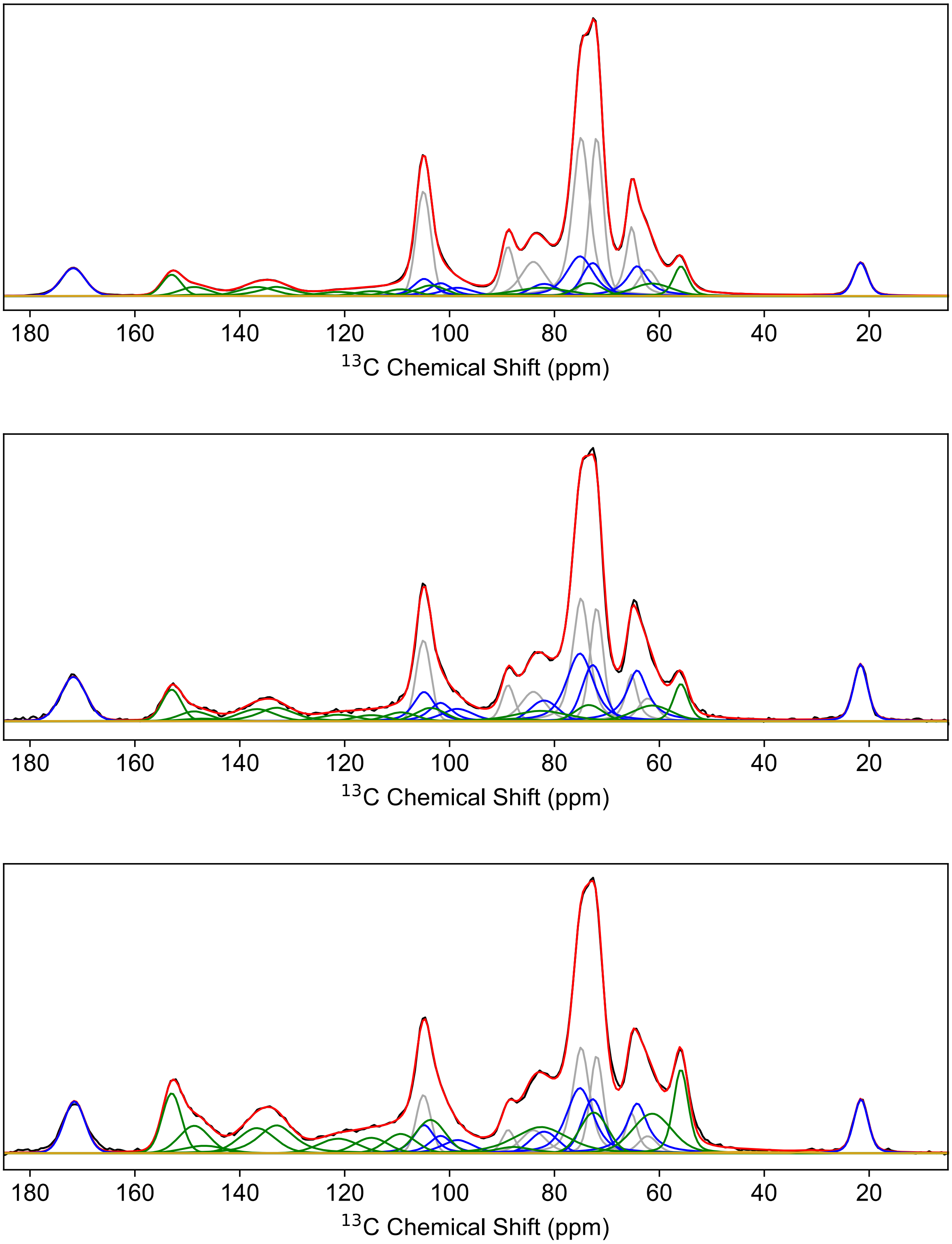

Fig. S25. 1D MultiCP-DARR Spectra, mixing time = 4000 ms. ^{13}C MultiCP-DARR (top), MultiCP-DARR-difference at 22 ppm selection (middle) and MultiCP-DARR-difference at 150 ppm selection (bottom) using τm = 4000 ms

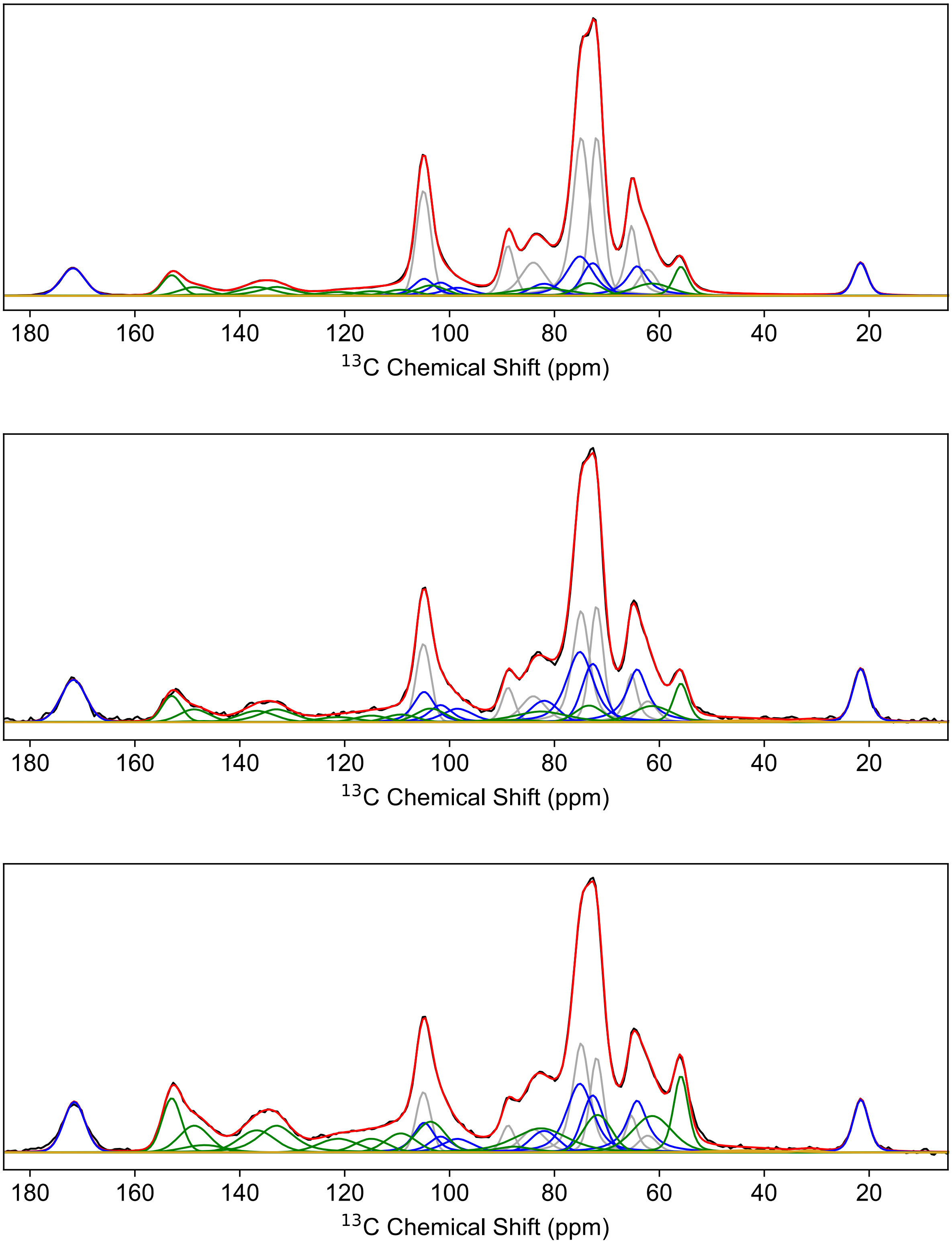

Fig. S26. 1D MultiCP-DARR Spectra, mixing time = 5000 ms. ^{13}C MultiCP-DARR (top), MultiCP-DARR-difference at 22 ppm selection (middle) and MultiCP-DARR-difference at 150 ppm selection (bottom) using τm = 5000 ms

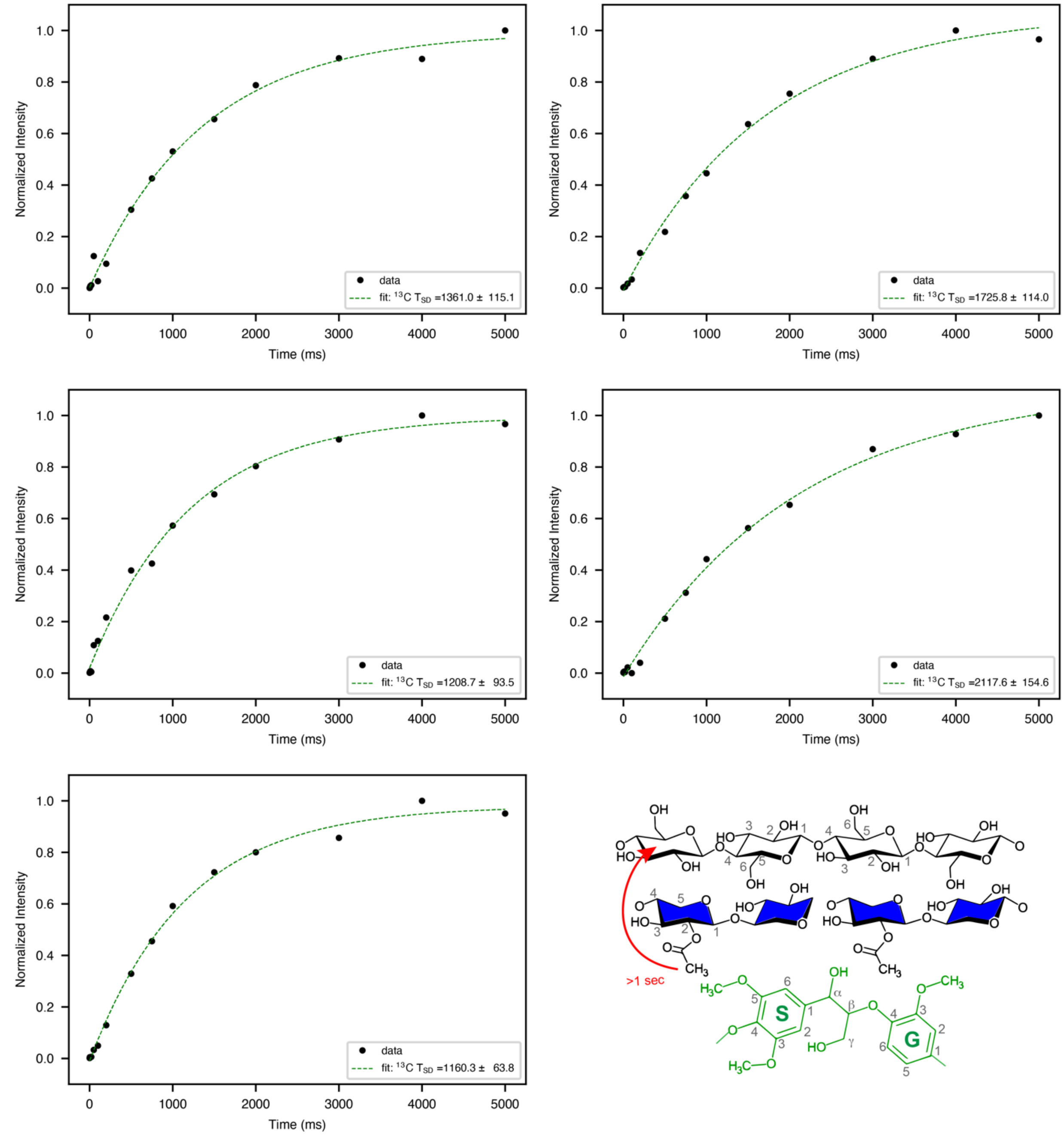

Fig. S27. Inter-polymer ^{13}C-^{13}C spin-diffusion buildup plots from xylan acetate methyl (source) to cellulose (sink) for 5 replicates. Spin-diffusion rate constants T_{SD} are longer than 1 second. Distance estimation is ~0.5 – 1 nm, but shorter than lignin-cellulose distance.

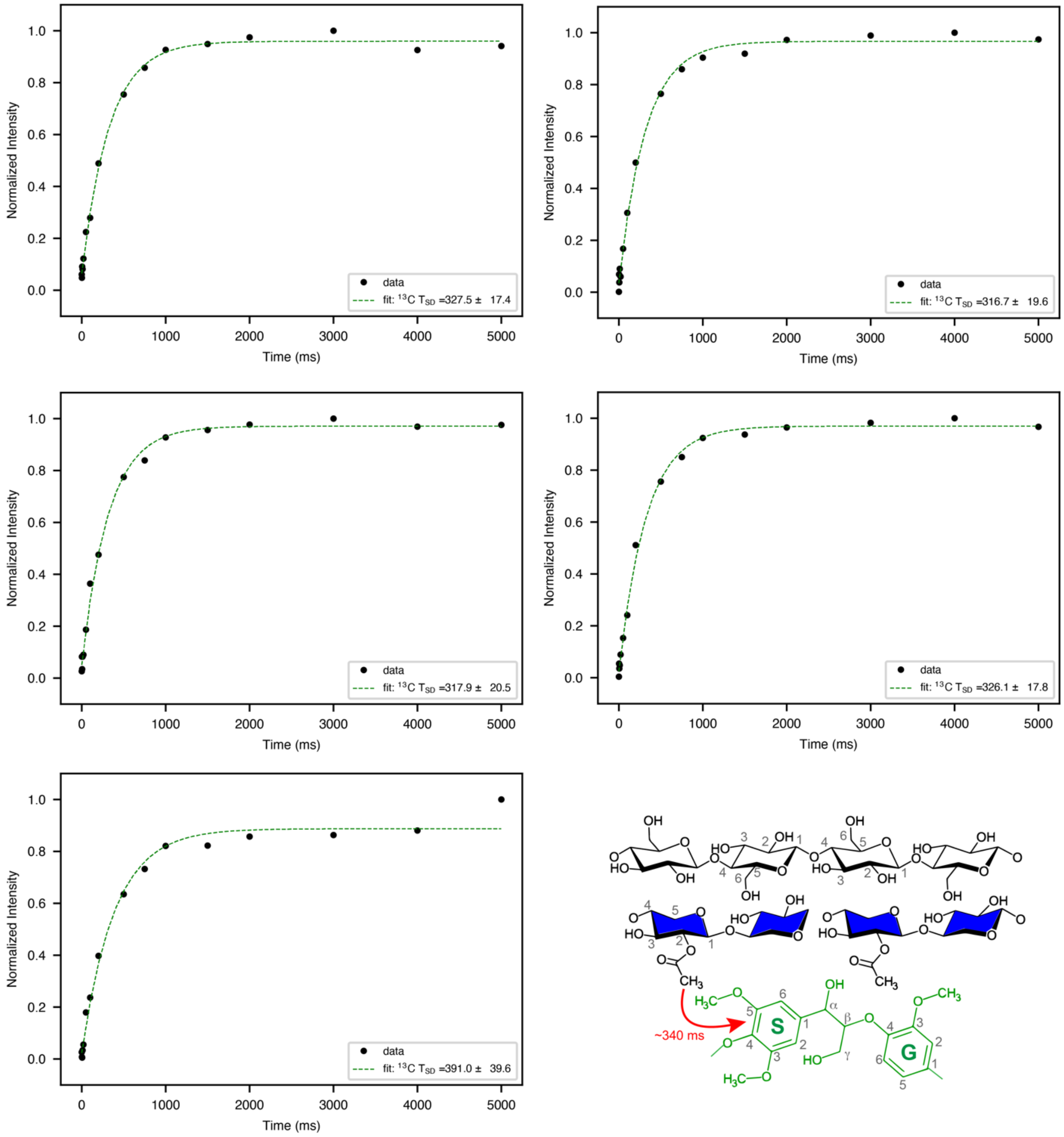

Fig. S28. Inter-polymer ^{13}C-^{13}C spin-diffusion buildup plots from xylan acetate methyl (source) to lignin S3,5 / G3,4 carbons near 150 ppm (sink) for 5 replicates. Spin-diffusion rate constants T_{SD} are ~340 ms. Xylan-lignin distance estimation is ~0.3-0.5 nm. Values are identical within error to the reverse spin diffusion case from lignin to xylan.

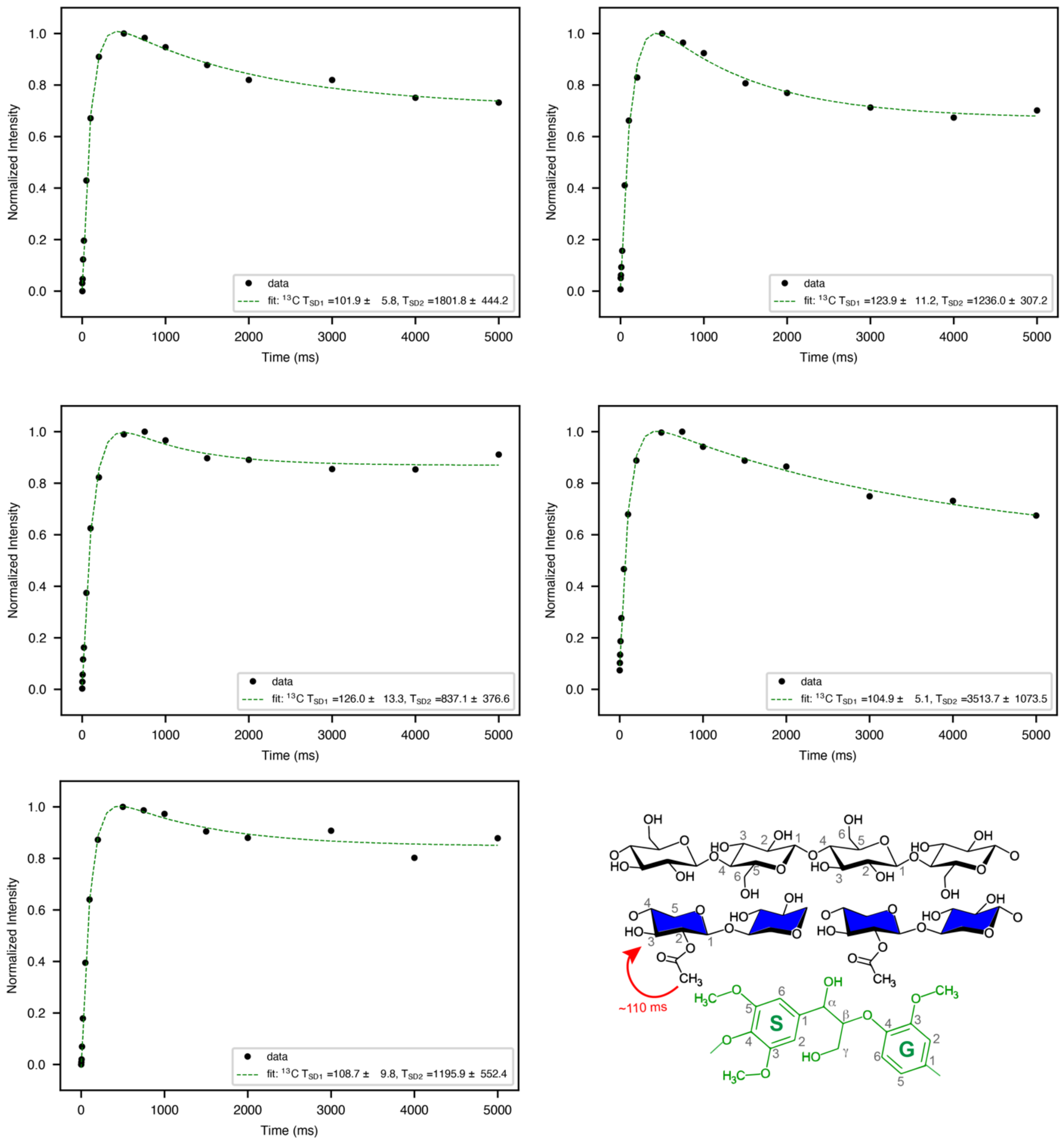

Fig. S29. Intra-polymer ^{13}C-^{13}C spin-diffusion buildup plots from xylan acetate methyl (source) to xylan backbone Xn2/Xn3 (sink) carbons for 5 replicates. Data are fit to two exponential functions representing magnetization transfer from Xn AcMe to Xn2/3 (T_{SD1}) and then outwards diffusion from Xn2/3 to other carbon sites (T_{SD2}). The spin-diffusion rate constant T_{SD1} for transfer from Xn AcMe to xylan backbone is ~110 ms, for a distance estimation of ~0.3 nm.

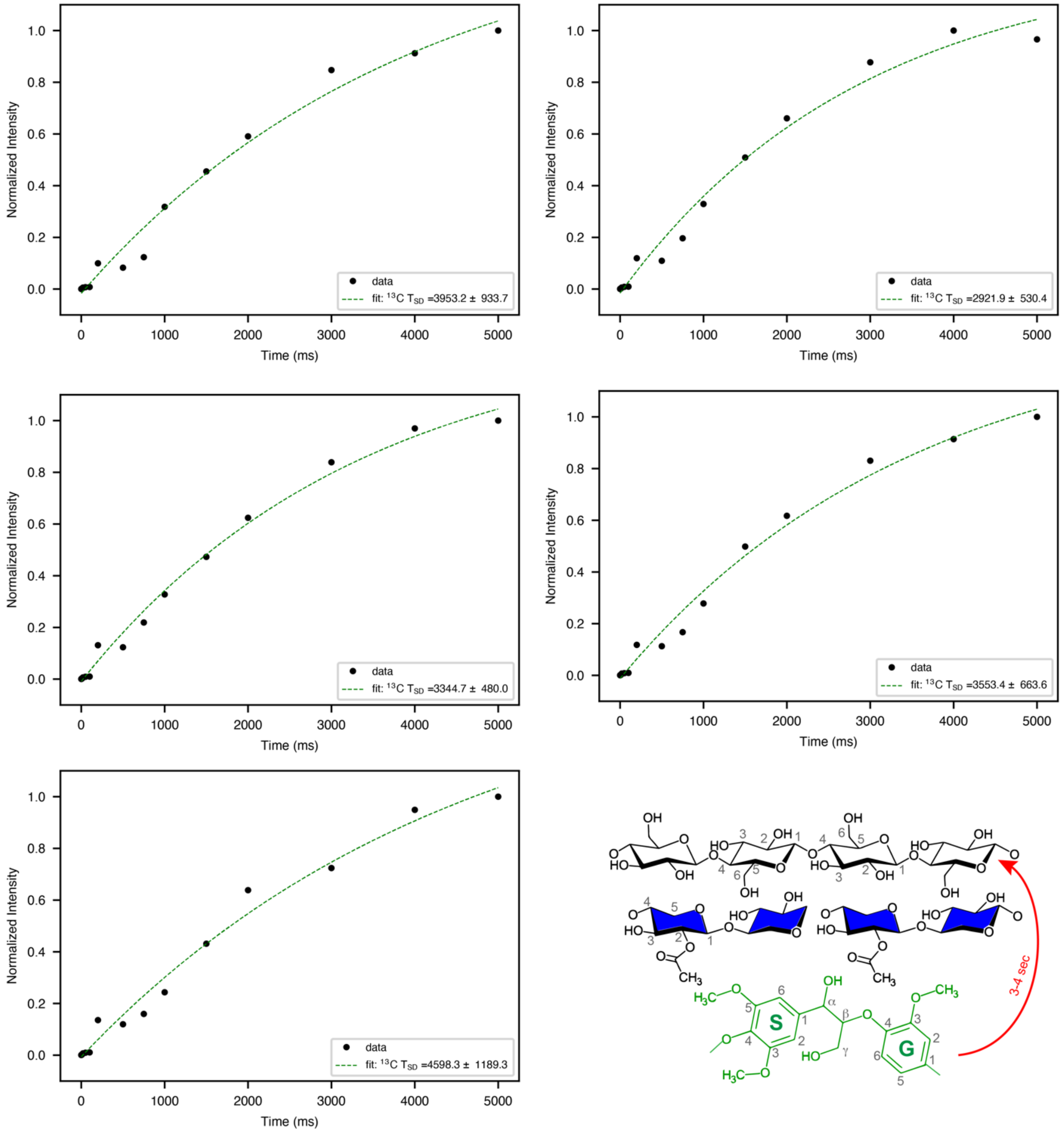

Fig. S30. Inter-polymer ^{13}C-^{13}C spin-diffusion buildup plots from lignin (source) to cellulose (sink) for 5 replicates. Spin-diffusion rate constants T_{SD} are longer than 2 seconds. Distance estimation is ~0.5 – 1 nm, but longer than xylan-cellulose distance.

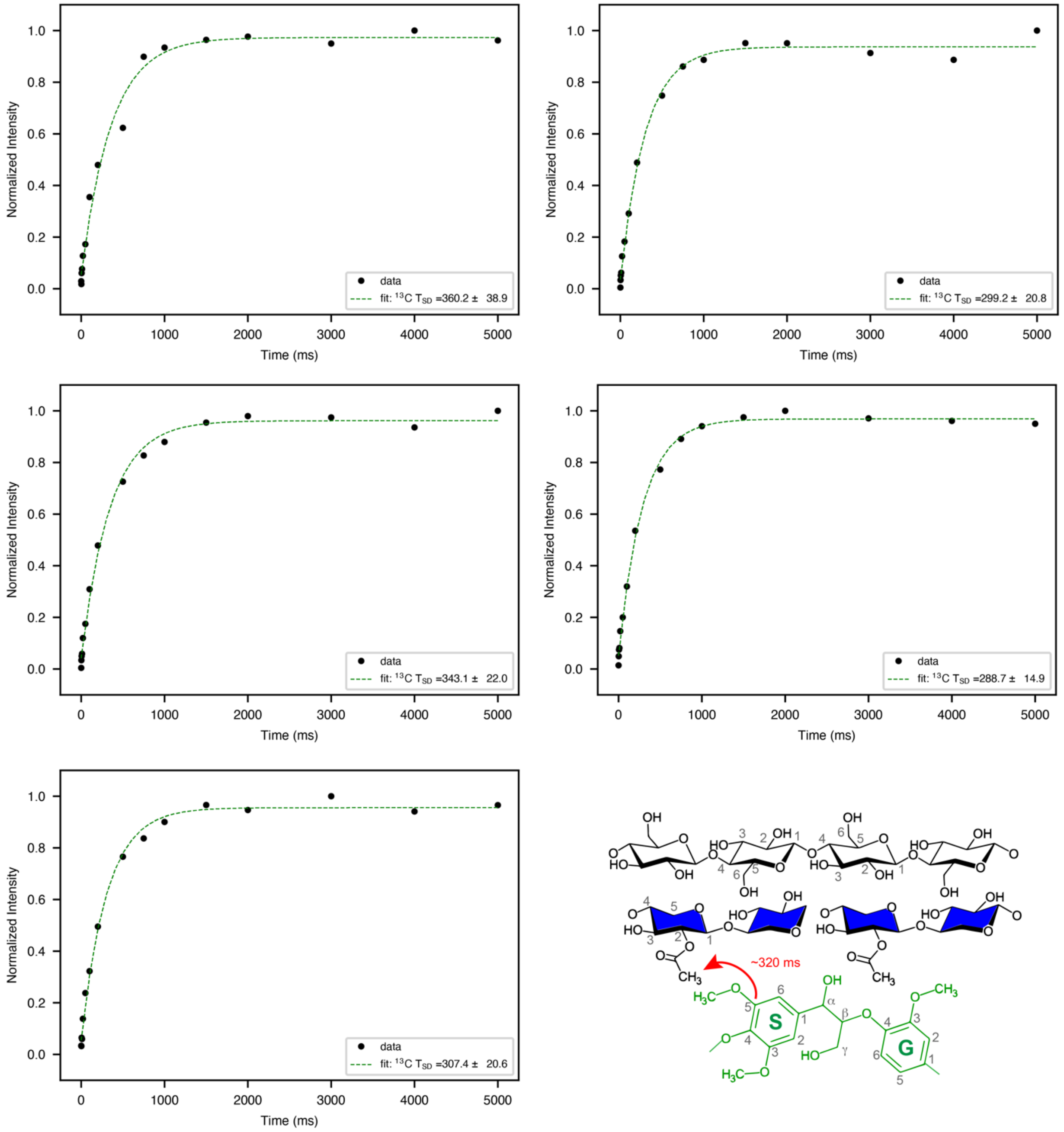

Fig. S31. Inter-polymer ^{13}C-^{13}C spin-diffusion buildup plots from lignin (source) to Xylan acetate methyl (sink) for 5 replicates. Spin-diffusion rate constants T_{SD} are ~320 ms. Lignin-xylan distance estimation is ~0.3-0.5 nm. Values are identical within error to the reverse spin diffusion case from xylan to lignin.

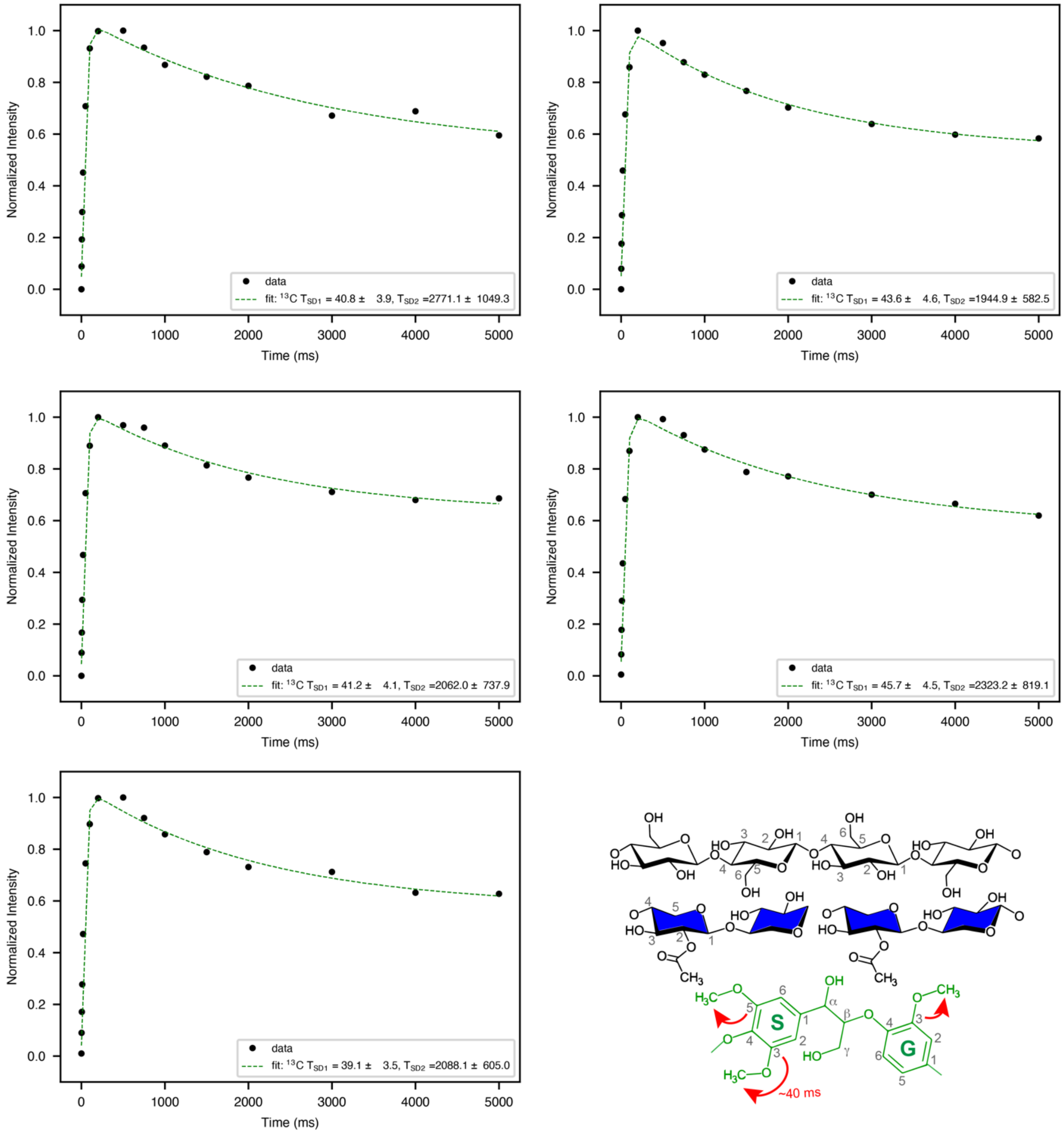

Fig. S32 Intra-polymer ^{13}C-^{13}C spin-diffusion buildup plots from lignin ring carbons (source) to lignin methoxy (sink) carbons for 5 replicates. Data are fit to two exponential functions representing magnetization transfer from S3,5 / G3,4 to OMe (T_{SD1}) and then outwards diffusion from OMe to other carbon sites (T_{SD2}). The spin-diffusion rate constant T_{SD1} for transfer from S3,5 / G3,4 to lignin OMe is ~40 ms, for a distance estimation of less than 0.3 nm.

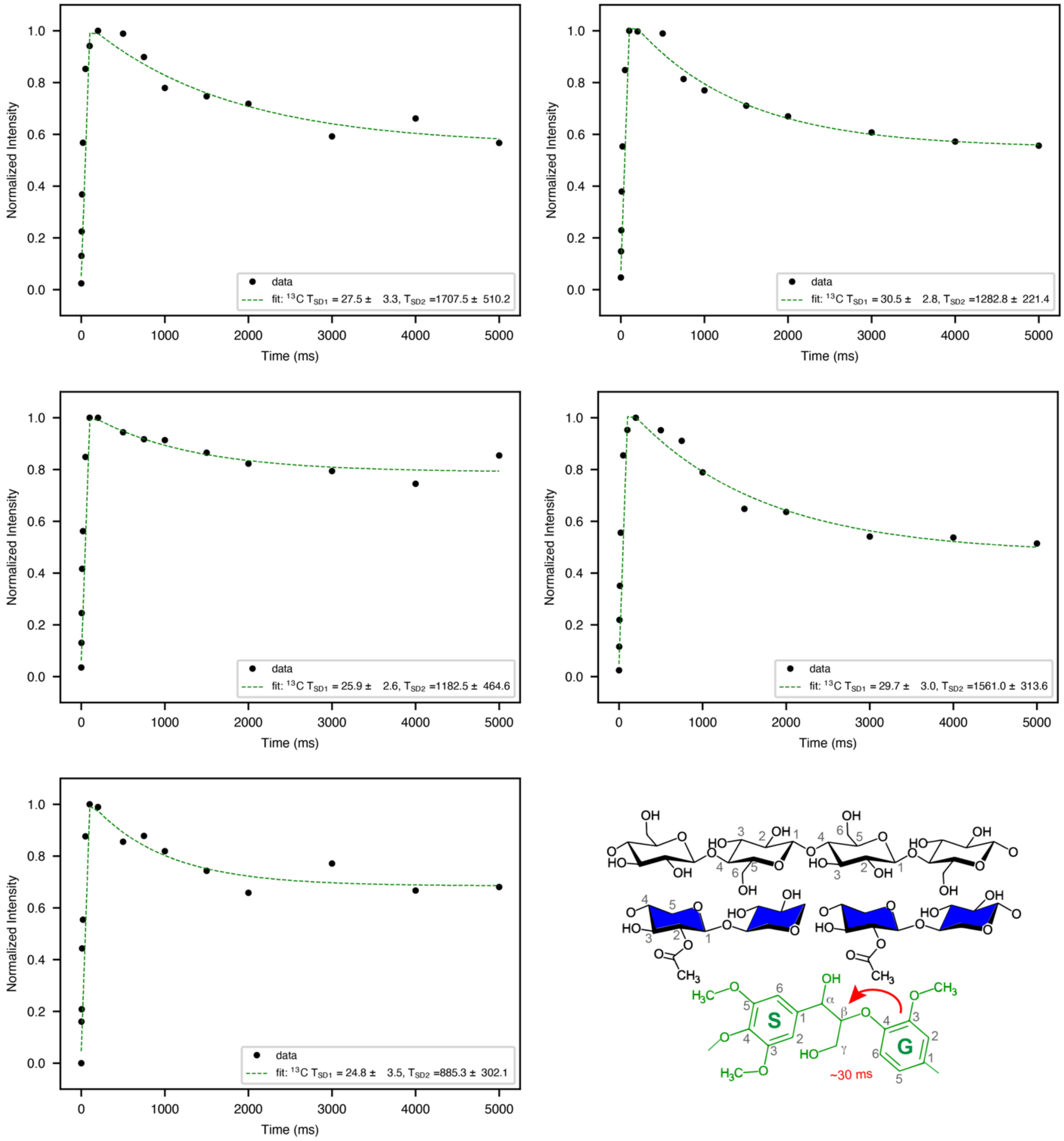

Fig. S33. Intra-polymer ^{13}C-^{13}C spin-diffusion buildup plots from lignin ring carbons (source) to lignin sidechain Cβ (sink) carbons for 5 replicates. Data are fit to two exponential functions representing magnetization transfer from S3,5 / G3,4 lignin sidechain Cβ (T_{SD1}) and then outwards diffusion from Cβ to other carbon sites (T_{SD2}). The spin-diffusion rate constant T_{SD1} for transfer from S3,5 / G3,4 to inter-lignin linkage Cβ units is ~30 ms, for a distance estimation of less than 0.3 nm.

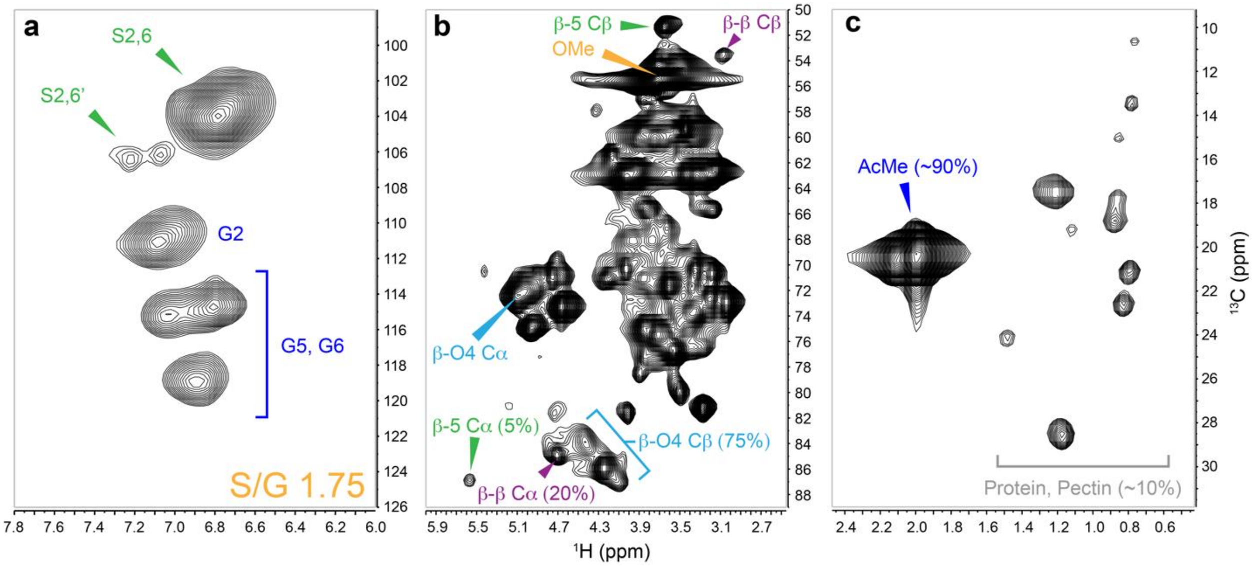

Fig. S34. Gel-state HSQC NMR (600 MHz) of ball-milled ^{13}C-enriched poplar wood used in this study. **(a)** Aromatic region used to calculate an S:G ratio of 1.75:1 for this sample. **(b)** Neutral carbohydrate and lignin sidechain region. 2D contour integrations of resolved signals reveal a β-O-4 : β-β : β-5 ratio of roughly 75 : 20 : 5. **(c)** Aliphatic region showing that about 90% of the signal in the 10-30 ppm range arises from acetate methyl carbons but ~10% comes from other moieties like protein, pectin and lipid. If we assume that protein and pectin aliphatic signals are not expected within 1 nm of lignin ring carbons, we can account for some (roughly 5%) of the observed magnetization recovery asymmetry – i.e., Lignin-to-Xylan magnetization recovery would increase from ~60% to ~65% if we remove possible protein/pectin from the calculation.

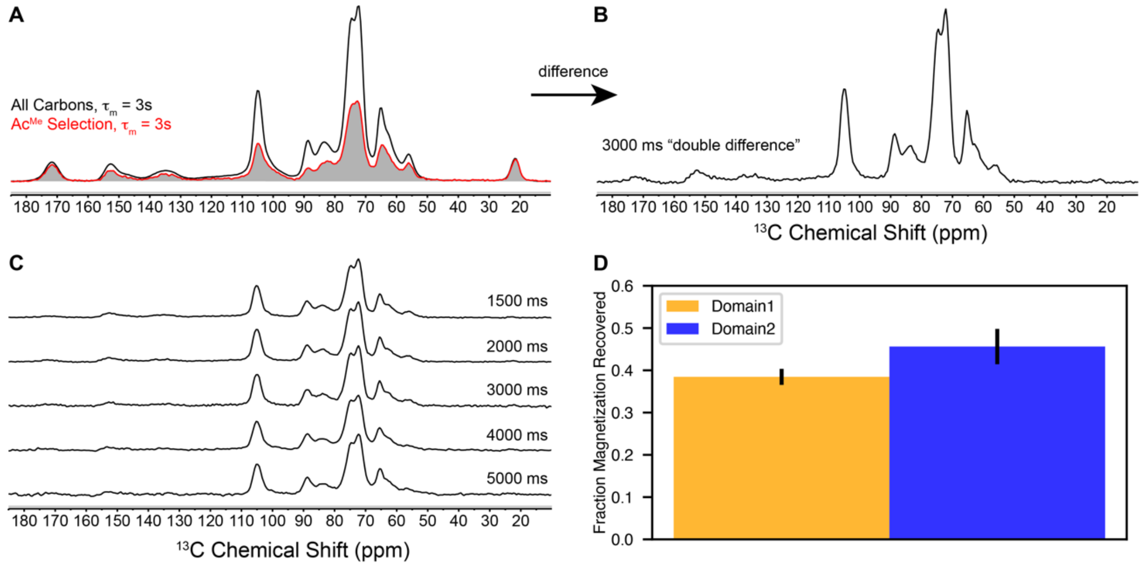

Fig. S35. Xylan-selected “double-difference” plots and magnetization recovery detail for cellulose domains 1 and 2. **(A)** Stacked plot of selective (red) and non-selective (black) 1D MultiCP-DARR spectra scaled by the intensity of the selected Xn AcMe signal. **(B)** Taking the difference between all carbons (black trace) and carbons within spin diffusion range from the selected signal (red trace) yields the ^{13}C sites that reside outside spin diffusion range. This “double-difference” spectrum is predominately cellulose with trace lignin. **(C)** double-difference plots obtained at 1500, 2000, 3000, 4000 and 5000 ms mixing times. At 1500 ms, about 15% of all cellulose signals are recovered so the double-difference plot represents ~85% of all cellulose, while at 5000 ms about 40% of all cellulose signals are recovered so the double-difference plot represents ~60% of all cellulose. The cellulose profiles are nearly identical, although domain1 signals may be somewhat more prevalent in the fiber interior. **(D)** Xylan-selected magnetization recovery values for domain1 (^{1}C4, ^{1}C6) and domain2 (^{2}C4, ^{2}C6) signals obtained from spectral deconvolution at the longest (5000 ms) spin diffusion mixing time averaged over 5 samples. Results confirm that both cellulose types are accessible to xylan, but there may be a slight preference for domain2 signals at the cellulose bundle surface.

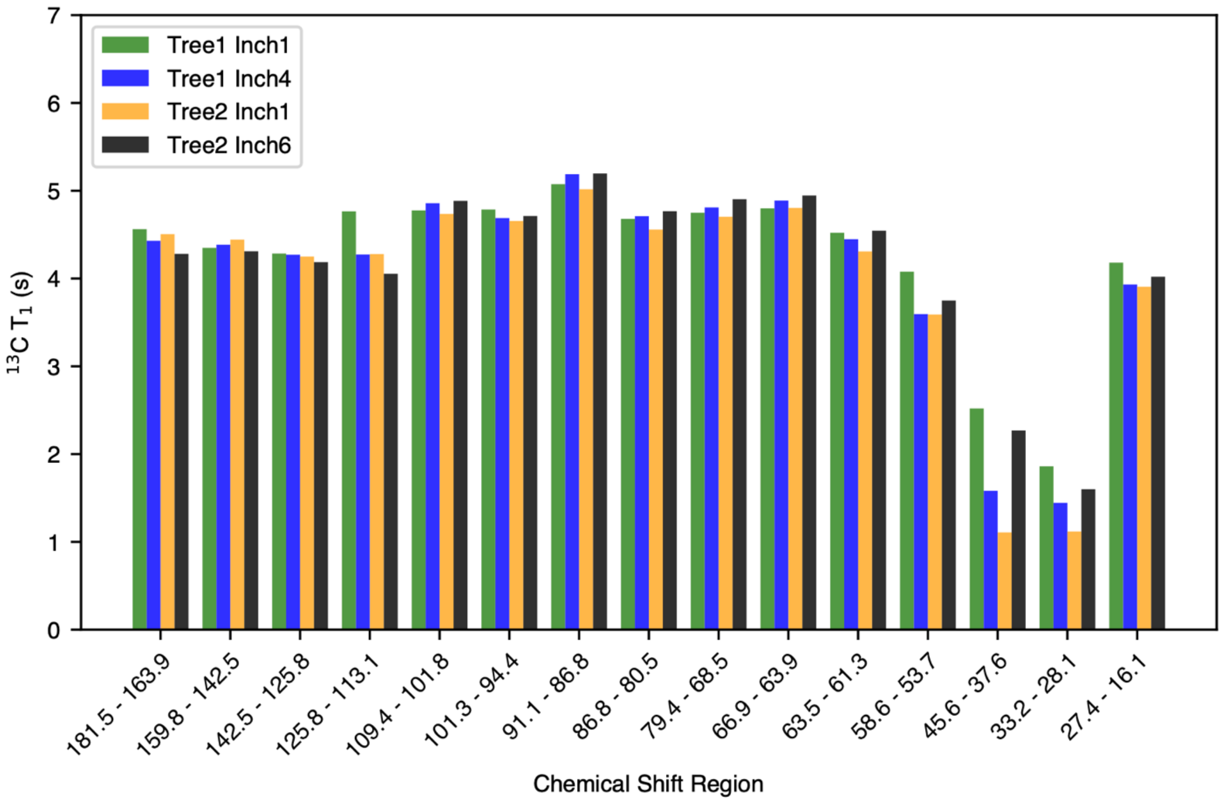

Fig. S36. Carbon-13 T_1 values for each chemical shift region. ^{13}C T_1 analysis on 4 of the 5 samples (duplicate measurements on each biological replicate) by the conventional Torchia method (Bruker sequence cpxt1). The well-averaged ^{13}C T_1 values caused by efficient ^{13}C-^{13}C spin diffusion between polymer groups bolster the overall conclusions of extensive polymer mixing on the nanometer length scale.

<table>
<tr><th>System</th><th>Cross-section</th><th>Cellulose (Gray)</th><th>Xylan (Green)</th><th>Lignin (Yellow)</th><th>Remarks</th><th>ssNMR Saturation Estimations vs. Expt.</th></tr>
<tr><td>a.4</td><td>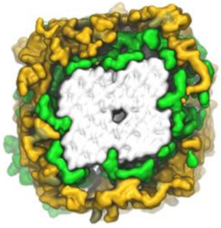</td><td>4x18chain microfibrils</td><td>Present as 2f, all bound on Cellulose surface</td><td>Placed on top of xylan to avoid contact with cellulose</td><td>-</td><td>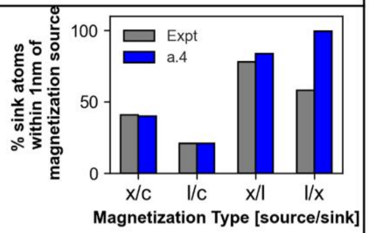
</td></tr>
<tr><td>b.4</td><td>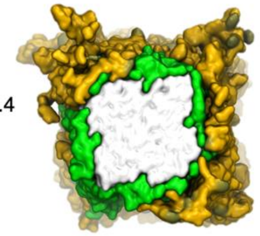</td><td>4x18chain microfibrils</td><td>Present as 2f, 70% of xylan chains bound on cellulose surface</td><td>Placed on top of cellulose-bound xylan with remaining xylan interspersed</td><td>Informed by NMR data suggesting 70:30 split in bound and unbound xylan</td><td>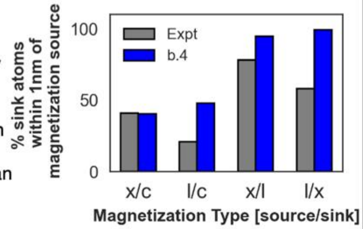
</td></tr>
<tr><td>c.4</td><td>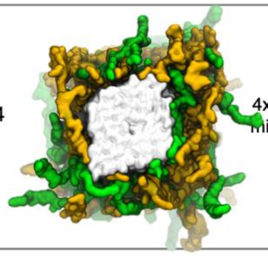</td><td>4x18chain microfibrils</td><td>Present as 2f, Xylan randomly interspersed with lignin</td><td>Lignin randomly interspersed with xylan</td><td>-</td><td>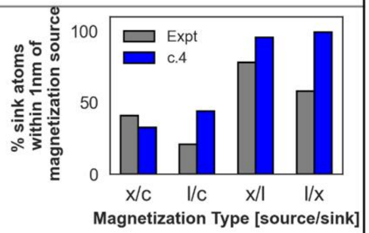
</td></tr>
<tr><td>d.4</td><td>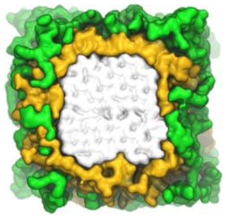</td><td>4x18chain microfibrils</td><td>Present as 3f Xylan placed on top of cellulose-bound Lignin</td><td>Lignin placed on top cellulose</td><td>Negative Control</td><td>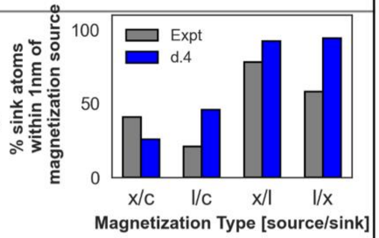
</td></tr>
<tr><td>e.4</td><td>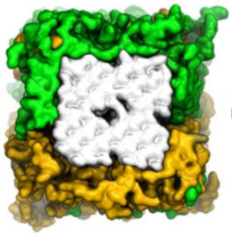</td><td>4x18chain microfibrils</td><td>Present as 3f All xylan bound to one (top) half of cellulose surface</td><td>All lignin bound to other (bottom) half of cellulose surface</td><td>Negative Control – Phase separated xylan and lignin</td><td>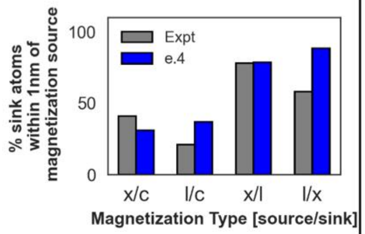
</td></tr>
</table>

Fig. S37. Molecular Models a-e, and comparison of simulation with experiment.

<table>
<tr><th>System</th><th>Cross-section</th><th>Cellulose (Gray)</th><th>Xylan (Green)</th><th>Lignin (Yellow)</th><th>Remarks</th><th>ssNMR Saturation Estimations vs. Expt.</th></tr>
<tr><td>f.4</td><td>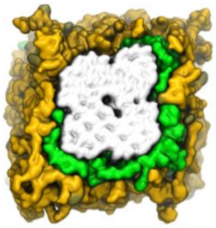</td><td>4x18chain microfibrils</td><td>Present as 3f all bound on Cellulose surface</td><td>Placed on top of xylan to avoid contact with cellulose</td><td>Negative Control – similar to a.4 but with xylan forced to be 3f</td><td>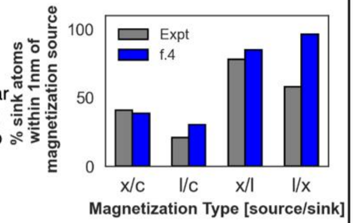
</td></tr>
<tr><td>g.4</td><td>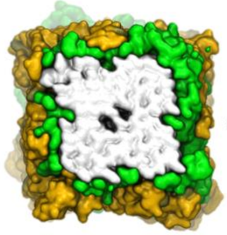</td><td>4x18chain microfibrils</td><td>70% Xylan bound to cellulose present as 2f</td><td>Lignin is globularized before binding to cellulose</td><td>-</td><td>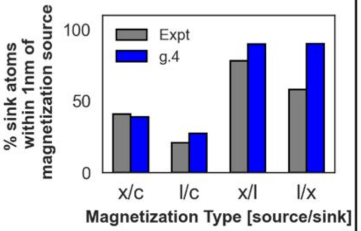
</td></tr>
<tr><td>h.4</td><td>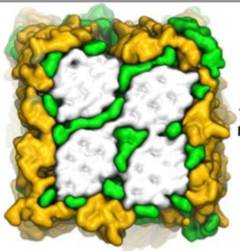</td><td>4x18chain microfibrils</td><td>Same as b.4 with some xylan isolated within cellulose</td><td>Lignin is globularized before binding to cellulose</td><td>-</td><td>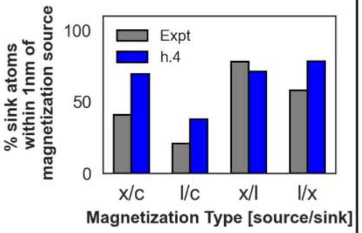
</td></tr>
<tr><td>b.8</td><td>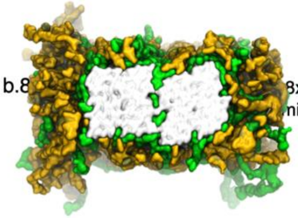</td><td>8x18chain microfibrils</td><td>Same as b.4 with some xylan isolated within cellulose</td><td>Same as b.4</td><td>-</td><td>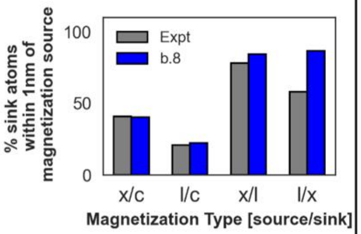
</td></tr>
<tr><td>b.10</td><td>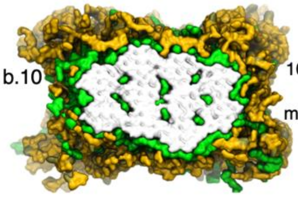</td><td>10x18chai n microfibrils</td><td>Same as b.4 with some xylan isolated within cellulose</td><td>Same as b.4</td><td>-</td><td>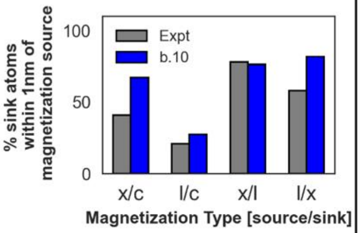
</td></tr>
</table>

Fig. S38. Molecular Models f.4, g.4, h.4, b.8 and b.10, and comparison of simulation with experiment.

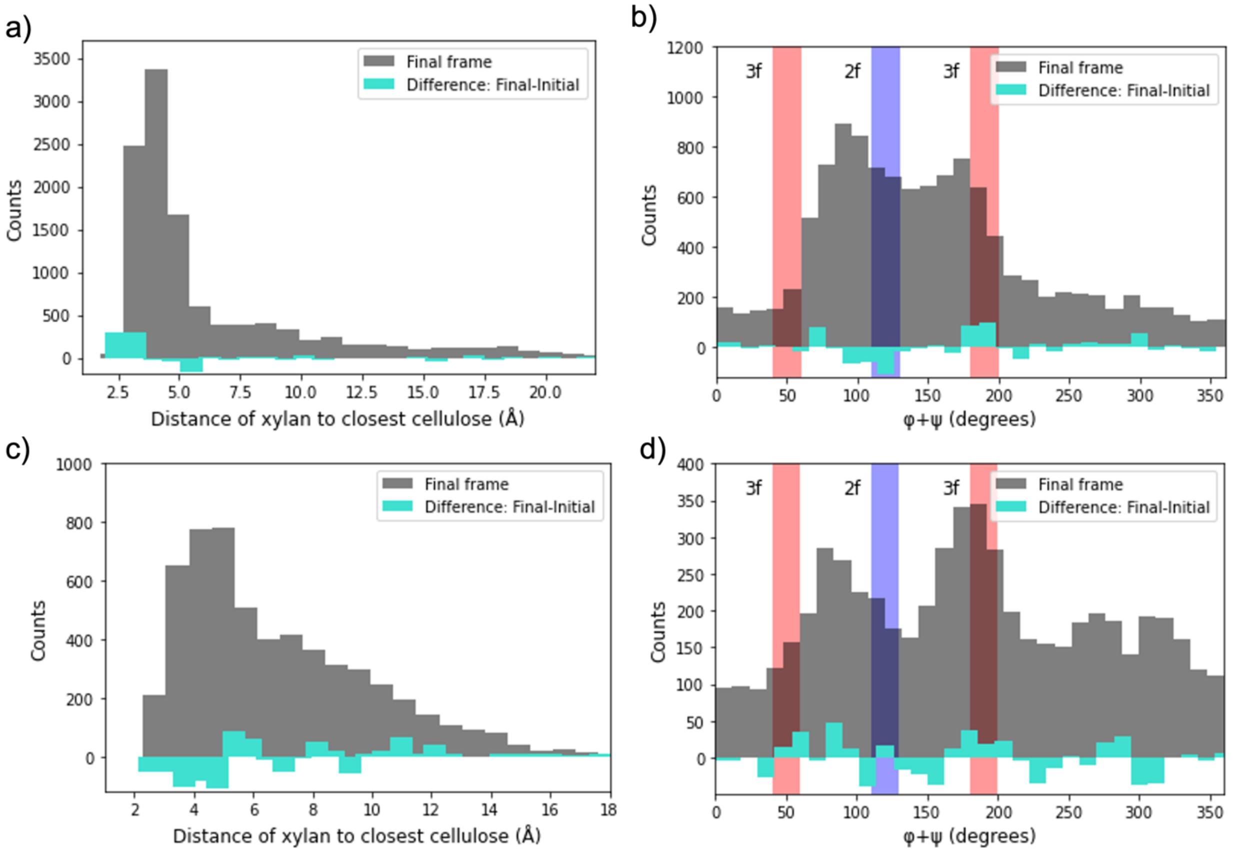

Fig. S39. The dynamics of xylan chain conformations. Histograms showing distances of xylan to the nearest cellulose carbon atom for the a.8 system **(a)** and the negative control system **(c)**. Graphs **(b)** and **(d)** show the sums of φ and ψ torsion angles between glycosidic oxygens on adjacent xylose residues for the a.8 and negative control systems, respectively. The data averaged over the final five frames of the simulation trajectories are shown in grey, the difference between initial and final frames is shown in light blue. Regions corresponding to characteristic xylan conformations are shown in red for threefold (50+/-10˚ and 190+/-10˚), and blue for twofold (120+/-10˚). The collective distribution of xylan location and conformation does not change during the simulation, while slight differences between frames for individual populations occurs.

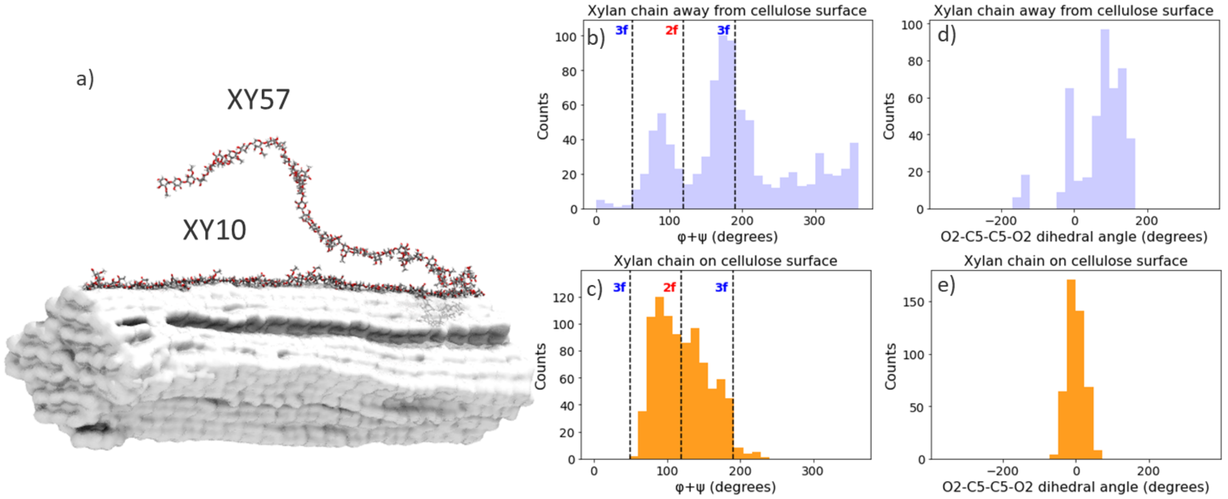

Fig. S40. Xylan conformational analysis of model b.8 at 6% moisture. (a) Snapshot of representative xylan chains close (X10) and far (X57) from the cellulose surface. (b) φ+ψ distributions for the xylan chain far from the cellulose surface. (c) φ+ψ distributions for the xylan chain close to cellulose surface. (d) θ^{2f}(O2-C5-C5-O2) values for the xylan chain far from the cellulose surface. (e) θ^{2f}(O2-C5-C5-O2) values for the xylan chain close to the cellulose surface.

A

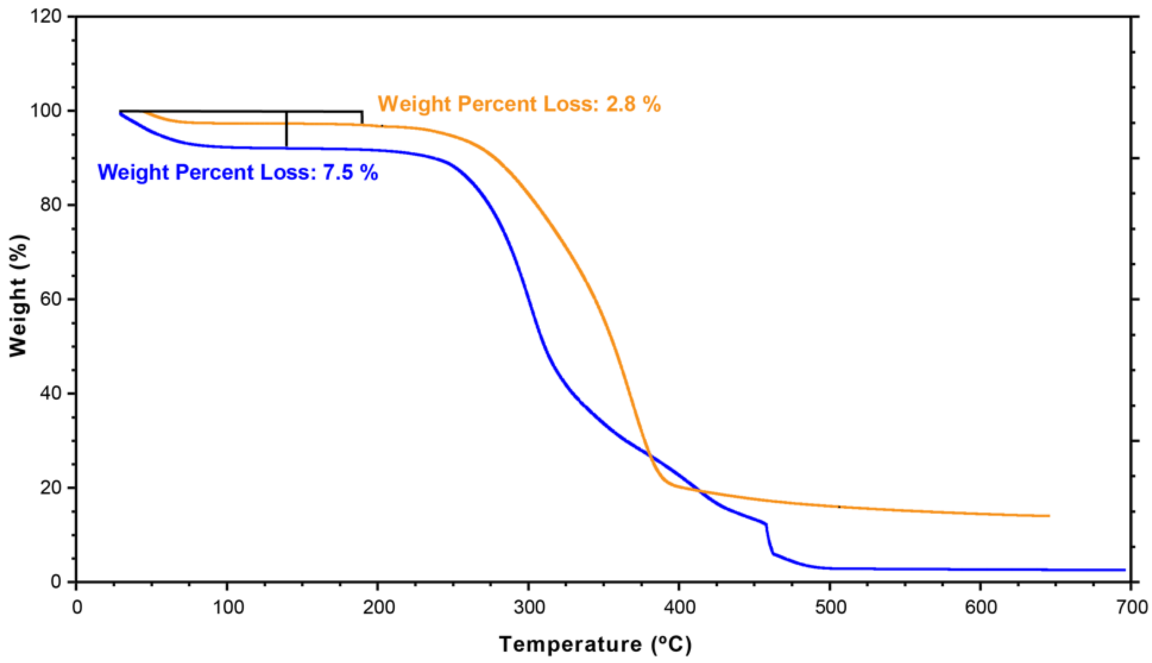

B

Sample	Weight (mg)	Oven Dry Weight (mg)	Moisture Content (%)
1	7.90	7.47	5.4 %
2	9.72	9.15	5.9 %
3	13.07	12.40	5.1 %

Fig. S41. Moisture content of poplar wood determined by (A) Thermogravimetric Analysis (TGA), and (B) oven-dry weight analysis shows 3-8% water content, depending on lab humidity. The relative humidity in the lab was 21% and 40% for orange and blue traces, and was 33% for oven-dry measurements. NMR measurements were performed on samples equilibrated at 20-30% lab humidity. Thermogravimetric Analysis (TGA) experiments were performed using a Discovery Series TGA 5500 (TA Instruments). Samples of 3-4 mg were loaded onto platinum pans for analysis. During analysis, the sample was purged with nitrogen gas at a flow rate of 25 mL/min. The sample was heated at a rate of 20 °C / min to a final temperature of 700 °C. TA Instruments Trios Software was used to do analysis on the samples. Moisture content estimates using oven-dry weight method were performed in triplicate by weighing ~8-14 mg biomass samples before drying (33% humidity) and after holding the samples in a vacuum oven for multiple days.

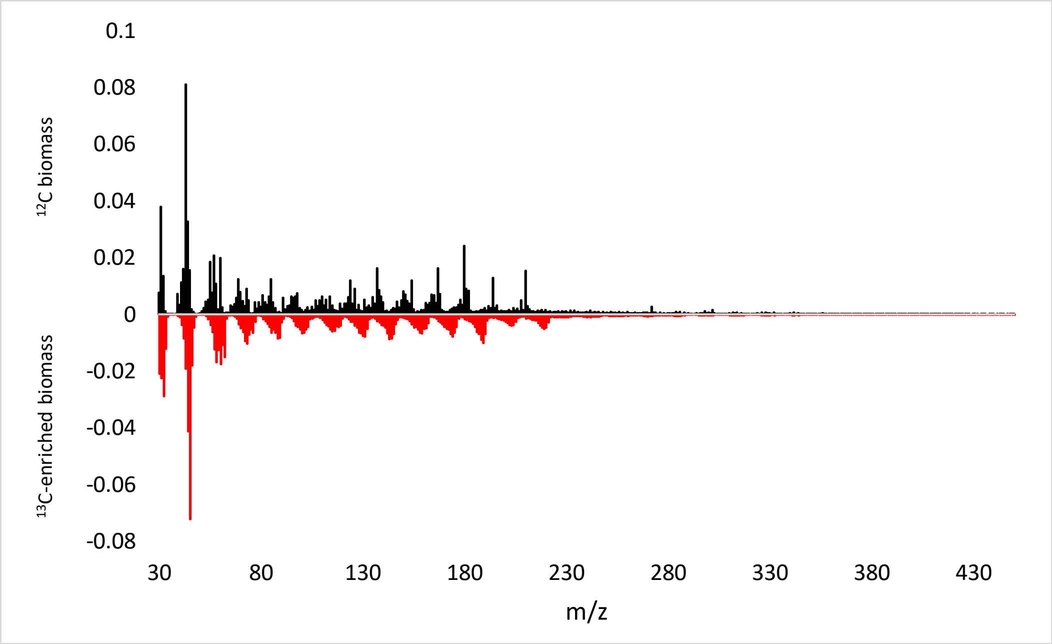

Fig. S42. py-MBMS spectral comparison of natural abundant and ^{13}C-enriched poplar wood

ssNMR Magnetization Type	Source Atoms	Sink Atoms
Xylan-sourced	methyl carbon (CA2) on the acetate group attached to xylose	Cellulose: C4 atom on Glucose<br>Lignin: Ring atoms C3 and C5 on the Syringyl residues and atoms C4 and C3 on the Guaicol residues
Lignin-sourced	Ring atoms C3 and C5 on the Syringyl residues and atoms C4 and C3 on the Guaicol residues of lignin	Cellulose: C4 atom on Glucose<br>Xylan: methyl carbon (CA2) on the acetate group attached to xylose

Table S1. Proximity Calculations for ssNMR estimations from MD equilibrated structures. The ssNMR saturation estimation (proximity) calculations were performed on equilibrated coordinates obtained at the end of the 50ns trajectory for each system. Specific source and sink atoms considered for the calculations are listed. The calculations involved counting the number of sink atoms within a certain distance from every source atom in each system. This calculation was performed for distances 5,6,7,8,9, and 10Å. The total number of sink atoms observed to be within a certain distance was divided by the total number of sink atoms in the system to obtain percentages. The saturation values reported in Fig. 4 and Fig. S37, S38, and Table S6 denote the percentages calculated at distance of 10Å.

	Experimental Composition (% Dry Weight)	Experiment ratio (weight)	Number of chains	Simulation ratio (weight)
cellulose	45%	2.25	72	2.20
hemicellulose	20%	1.00	30	1.00
lignin	25%	1.25	58	1.22

Table S2. The composition of SCW models with four cellulose bundles. Experimental composition numbers come from Sannigrahi 2010(*61*) and closely match compositional analysis for 8 week old DN34 stems (Table S8). The number of biopolymer chains were scaled up accordingly for the models containing eight or ten cellulose bundles.

Cellulose			Xylan			Lignin		
Shift (ppm)	Signal	FWHM (ppm)	Shift (ppm)	Assignment	FWHM (ppm)	Shift (ppm)	Assignment	FWHM (ppm)
105.0	C1	1.6	82.0	Xn4 2f, 3f	3.2	152.9	S3,5	2.3
88.8	^{1}C4	1.5	74.9	Xn3	2.9	148.6	G3,4 A	3.4
84.0	^{2}C4	2.7	72.4	Xn2	2.5	146.6	G3,4 B	3.9
75.0	C3	1.9	64.2	Xn5	2.3	137.2	S1,4, G1 A	4.1
72.0	C2, C5	1.5	171.8	Xn AcCO	2.4	133.2	S1,4, G1 B	4.2
65.4	^{1}C6	1.2	104.7	Xn1 2f	2.3	121.7	G 120	4.4
62.4	^{2}C6	2.3	101.4	Xn1 3f	2.4	115.3	G 115	4.0
			98	Other	3.2	109.6	G 110	3.5
			21.61	Xn AcMe	3.10	103.8	S2,6	3.5
						87.7	β-β, β-5	4.1
						83	βO4 Cβ	5.8
						73.5	βO4 Cα	3.2
						61.5	βO4 Cγ	4.2
						56.0	OMe	1.7

Table S3. Table of chemical shifts and peak widths after 2D-informed spectral deconvolution. Final deconvolution parameters shown in the table above are from 2D-informed fitting the three 1D training spectra. The three primary training spectra used for spectral deconvolution of lignin, xylan and cellulose were respectively 1) Lignin-selective (~150 ppm) spectrum using 20 or 50 ms mixing time, 2) Xylan-selective (22 ppm) spectrum using 100 or 200 ms mixing time, and 3) the double-difference spectrum obtained from subtracting the 3-second Xylan-selective (22 ppm) spectrum from the equivalent 3-second non-selective 1D DARR spectrum. First, peak centers and initial estimates for peak widths were extracted using a combination of 2D ^{13}C-^{13}C correlation data collected at both 200 and 600 MHz. Then, initial guesses were imported into MestreNova's Global Spectral Deconvolution (GSD) module, peak positions and initial linewidths were locked, and a first deconvolution step was performed. Then peak positions and linewidths were unlocked and fitting was repeated to minimize residuals. All ^{13}C chemical shifts were compared to and validated against prior literature assignments for poplar wood in the solid state.(*19*)

Sample	Source	Sink	T_{SD} (ms)	Error T_{SD} (ms)	Avg T_{SD1} (ms)	STDEV T_{SD} (ms)
Tree1 Replicate1	Xn AcMe	Lignin 135 ppm	370	26		
Tree1 Replicate2	Xn AcMe	Lignin 135 ppm	308	24		
Tree1 Replicate3	Xn AcMe	Lignin 135 ppm	314	23	315	33
Tree2 Replicate1	Xn AcMe	Lignin 135 ppm	293	25		
Tree2 Replicate2	Xn AcMe	Lignin 135 ppm	288	25		
Tree1 Replicate1	Xn AcMe	Lignin 150 ppm	327	17		
Tree1 Replicate2	Xn AcMe	Lignin 150 ppm	303	17		
Tree1 Replicate3	Xn AcMe	Lignin 150 ppm	316	20	333	34
Tree2 Replicate1	Xn AcMe	Lignin 150 ppm	326	18		
Tree2 Replicate2	Xn AcMe	Lignin 150 ppm	391	40		
Tree1 Replicate1	Xn AcMe	Lignin OMe	239	28		
Tree1 Replicate2	Xn AcMe	Lignin OMe	278	21		
Tree1 Replicate3	Xn AcMe	Lignin OMe	299	41	260	27
Tree2 Replicate1	Xn AcMe	Lignin OMe	226	21		
Tree2 Replicate2	Xn AcMe	Lignin OMe	264	23		
Tree1 Replicate1	Xn AcMe	Sum All Cellulose	1361	115		
Tree1 Replicate2	Xn AcMe	Sum All Cellulose	1689	102		
Tree1 Replicate3	Xn AcMe	Sum All Cellulose	1270	97	1520	389
Tree2 Replicate1	Xn AcMe	Sum All Cellulose	2118	155		
Tree2 Replicate2	Xn AcMe	Sum All Cellulose	1160	64		

Table S4. Summary of ^{13}C-^{13}C inter-polymer spin-diffusion rate constants T_{SD} for from xylan (source) to lignin and cellulose inter-polymer interactions. Data from five replicates as well as averages and standard deviations are shown. Errors for each individual fit are derived directly from the variance of the fits.

Sample	Source	Sink	T_{SD} (ms)	Error T_{SD} (ms)	Avg T_{SD1} (ms)	STDEV T_{SD} (ms)
Tree1 Replicate1	Lignin 150 ppm	Xn AcMe	360	39		
Tree1 Replicate2	Lignin 150 ppm	Xn AcMe	297	19		
Tree1 Replicate3	Lignin 150 ppm	Xn AcMe	343	22	319	31
Tree2 Replicate1	Lignin 150 ppm	Xn AcMe	289	15		
Tree2 Replicate2	Lignin 150 ppm	Xn AcMe	307	21		
Tree1 Replicate1	Lignin 150 ppm	Xn AcCO	314	18		
Tree1 Replicate2	Lignin 150 ppm	Xn AcCO	345	17		
Tree1 Replicate3	Lignin 150 ppm	Xn AcCO	327	28	335	15
Tree2 Replicate1	Lignin 150 ppm	Xn AcCO	354	25		
Tree2 Replicate2	Lignin 150 ppm	Xn AcCO	335	28		
Tree1 Replicate1	Lignin 150 ppm	Sum Hemicellulose	376	36		
Tree1 Replicate2	Lignin 150 ppm	Sum Hemicellulose	321	15		
Tree1 Replicate3	Lignin 150 ppm	Sum Hemicellulose	346	17	341	22
Tree2 Replicate1	Lignin 150 ppm	Sum Hemicellulose	337	18		
Tree2 Replicate2	Lignin 150 ppm	Sum Hemicellulose	323	17		
Tree1 Replicate1	Lignin 150 ppm	Sum All Cellulose	3953	934		
Tree1 Replicate2	Lignin 150 ppm	Sum All Cellulose	3027	534		
Tree1 Replicate3	Lignin 150 ppm	Sum All Cellulose	3345	480	3695	607
Tree2 Replicate1	Lignin 150 ppm	Sum All Cellulose	3553	664		
Tree2 Replicate2	Lignin 150 ppm	Sum All Cellulose	4598	1189		

Table S5. Summary of ^{13}C-^{13}C inter-polymer spin-diffusion rate constants T_{SD} for from lignin (source) to xylan and cellulose sinks. Data from five replicates as well as averages and standard deviations are shown. Errors for each individual fit are derived directly from the variance of the fits.

	Saturated % of Sink atoms within 1 nm of Source Atoms (source) to (sink)			
	Lignin to Cellulose	Lignin to Xylan	Xylan to Cellulose	Xylan to Lignin
Model b.8 with 3% moisture	22.4	86.7	40.2	84.4
Model b.8 with 6% moisture	22.5	85.8	41.2	83.5

Table S6. Proximity Analysis for 8Fiber system b.8 with 3% and 6% moisture content. Saturated proximity values (1 nm upper limit) suggest little to no differences in proximity values at the differing moisture contents.

Sample	X2L	X2C	L2X	L2C
**Rep1**	0.75	0.41	0.59	0.20
**Rep2**	0.80	0.41	0.60	0.18
**Rep3**	0.77	0.36	0.58	0.19
**Rep4**	0.81	0.44	0.59	0.19
**Rep5**	0.79	0.42	0.56	0.20
**Mean**	0.78	0.41	0.58	0.19
**Std Error of Mean**	0.03	0.02	0.03	0.01
**Untreated1 (p value)**	0.767 (0.65)	0.389 (0.36)	0.607 (0.45)	0.203 (0.32)
**Untreated2 (p value)**	0.730 (0.18)	0.377 (0.18)	0.574 (0.74)	0.214 (0.07)

Table S7. Magnetization recovery values at 5000 ms for all samples. The first five samples (Rep1-Rep5) were destarched and extracted, and the last two are from wood that was washed with DI water and allowed to air dry but otherwise untreated. Xylan to Lignin: X2L, Xylan to Cellulose: X2C, Lignin to Xylan: L2X, Lignin to Cellulose: L2C. Standard Error of Mean values are derived from the signal to noise. Based on the single-sample t test (p-values in parentheses), magnetization recovery values X2L, X2C, L2X, and L2C are not statistically different between untreated and destarched/extracted wood ($p > 0.05$).

% Ash	% Whole Protein	% Lignin	% Glucan	% Xylan	% Galactan	% Arabinan	% Mannan	Uronic acid	Acetyl	Total %
0.33	-	22.36	42.89	14.96	1.95	0.79	2.80	-	4.69	90.76

Table S8. Compositional Analysis of DN34 Poplar wood based on NREL LAP NREL/TP-510-42618. Values closely match those of DN34 hybrid poplar (Sannigrahi 2010) suggesting ssNMR results presented in this work represent lignified secondary wall woody material with minimal young and developing primary wall.

Base m/z	43	124	135	137	180	194	208	210	Average	% enrichment
Ratio $^{13}C/^{12}C$	0.24	0.23	0.19	0.16	0.07	0.11	0.20	0.10	0.16	84
**m/z enriched**	**45**	**131**	**144**	**146**	**188**	**205**	**219**	**221**		
Ratio $^{13}C/^{12}C$	4.67	1.54	5.13	1.75	5.77	1.93	5.55	3.31	3.71	73

Base % enriched = 100*(1-average ratio)
Enrichment based on enriched m/z = 100*(1-(1/average ratio))

Table S9. Estimation of ^{13}C enrichment by py-MBMS. Poplar biomass samples with and without ^{13}C enrichment were analyzed by py-MBMS where ion intensities were normalized based on each sample total ion chromatogram. The relative abundance of annotated ions attributed to primarily lignin-derived species were used to estimate approximate ^{13}C enrichment in the labelled biomass. For example, the ratio of ion intensities m/z 43 (C2), 124 (C7), 135 (C9), 137 (C9), 180 (C8), 194 (C11), 208 (C11), 210 (C11) with number of carbons indicated in () were calculated for the ^{13}C to ^{12}C biomass samples. Ratios of corresponding species where each C would be labelled were also calculated but which minimizes overlap from other abundant fragments, listed respectively: m/z 45 (C2), 131 (C7), 144 (C9), 146 (C9), 188 (C8), 219 (C11), 221 (C11). These estimates are calculated using fragments from pyrolysates assuming each pyrolysate is 100% enriched that are generated from biomass thermolysis and not necessarily quantitative particularly as they do not account for partially enriched fragments or differentiation of enrichment, but spectral patterns are consistent with substantial ^{13}C enrichment in biopolymers (see Fig. S42 for spectral comparison). Using enrichment calculations from base and enriched fragments, we can estimate that biomass secondary cell wall enrichment is on the order of 80%.

References: See main draft

REFERENCES AND NOTES

1. Y. Pan, R. A. Birdsey, J. Fang, R. Houghton, P. E. Kauppi, W. A. Kurz, O. L. Phillips, A. Shvidenko, S. L. Lewis, J. G. Canadell, P. Ciais, R. B. Jackson, S. W. Pacala, A. D. McGuire, S. Piao, A. Rautiainen, S. Sitch, D. Hayes, A large and persistent carbon sink in the world's forests. *Science* **333**, 988–993 (2011).

2. H. Zhu, W. Luo, P. N. Ciesielski, Z. Fang, J. Y. Zhu, G. Henriksson, M. E. Himmel, L. Hu, Wood-derived materials for green electronics, biological devices, and energy applications. *Chem. Rev.* **116**, 9305–9374 (2016).

3. C. Somerville, H. Youngs, C. Taylor, S. C. Davis, S. P. Long, Feedstocks for lignocellulosic biofuels. *Science* **329**, 790–792 (2010).

4. L. Petridis, J. C. Smith, Molecular-level driving forces in lignocellulosic biomass deconstruction for bioenergy. *Nat. Rev. Chem.* **2**, 382–389 (2018).

5. M. E. Himmel, S. Y. Ding, D. K. Johnson, W. S. Adney, M. R. Nimlos, J. W. Brady, T. D. Foust, Biomass recalcitrance: Engineering plants and enzymes for biofuels production. *Science* **315**, 804–807 (2007).

6. X. Zhao, L. Zhang, D. Liu, Biomass recalcitrance. Part I: The chemical compositions and physical structures affecting the enzymatic hydrolysis of lignocellulose. *Biofuels Bioprod. Biorefin.* **6**, 465–482 (2012).

7. C. Chen, Y. Kuang, S. Zhu, I. Burgert, T. Keplinger, A. Gong, T. Li, L. Berglund, S. J. Eichhorn, L. Hu, Structure–property–function relationships of natural and engineered wood. *Nat. Rev. Mater.* **5**, 642–666 (2020).

8. G. T. Beckham, J. F. Matthews, B. Peters, Y. J. Bomble, M. E. Himmel, M. F. Crowley, Molecular-level origins of biomass recalcitrance: Decrystallization free energies for four common cellulose polymorphs. *J. Phys. Chem. B* **115**, 4118–4127 (2011).

9. P. N. Ciesielski, R. Wagner, V. S. Bharadwaj, J. Killgore, A. Mittal, G. T. Beckham, S. R. Decker, M. E. Himmel, M. F. Crowley, Nanomechanics of cellulose deformation reveal molecular defects that facilitate natural deconstruction. *Proc. Natl. Acad. Sci. U.S.A.* **116**, 9825–9830 (2019).

10. K. Kulasinski, D. Derome, J. Carmeliet, Impact of hydration on the micromechanical properties of the polymer composite structure of wood investigated with atomistic simulations. *J. Mech. Phys. Solids* **103**, 221–235 (2017).

11. C. Zhang, M. Chen, Keten, B. Coasne, D. Derome, J. Carmeliet, Hygromechanical mechanisms of wood cell wall revealed by molecular modeling and mixture rule analysis *Sci. Adv.* **7**, eabi8919 (2021).

12. A. McDermott, T. Polenova, *Solid State NMR Studies of Biopolymers* (Wiley, 2012), 592 pp.

13. K. Schmidt-Rohr, H. W. Spies, *Multidimensional Solid-State NMR and Polymers* (Academic Press, 1994), 478 pp.

14. B. Reif, S. E. Ashbrook, L. Emsley, M. Hong, Solid-state NMR spectroscopy. *Nat. Rev. Methods Primers* **1**, 2 (2021).

15. R. Dupree, T. J. Simmons, J. C. Mortimer, D. Patel, D. Iuga, S. P. Brown, P. Dupree, Probing the molecular architecture of *Arabidopsis thaliana* secondary cell walls using two- and three-dimensional ^{13}C solid state nuclear magnetic resonance spectroscopy. *Biochemistry* **54**, 2335–2345 (2015).

16. N. J. Grantham, J. Wurman-Rodrich, O. M. Terrett, J. J. Lyczakowski, K. Stott, D. Iuga, T. J. Simmons, M. Durand-Tardif, S. P. Brown, R. Dupree, M. Busse-Wicher, P. Dupree, An even pattern of xylan substitution is critical for interaction with cellulose in plant cell walls. *Nat. Plants* **3**, 859–865 (2017).

17. T. J. Simmons, J. C. Mortimer, O. D. Bernardinelli, A. C. Pöppler, S. P. Brown, E. R. deAzevedo, R. Dupree, P. Dupree, Folding of xylan onto cellulose fibrils in plant cell walls revealed by solid-state NMR. *Nat. Commun.* **7**, 13902 (2016).

18. B. Addison, D. Stengel, V. S. Bharadwaj, R. M. Happs, C. Doeppke, T. Wang, Y. J. Bomble, G. P. Holland, A. E. Harman-Ware, Selective one-dimensional ^{13}C–^{13}C spin-diffusion solid-state nuclear magnetic resonance methods to probe spatial arrangements in biopolymers including plant cell walls, peptides, and spider silk. *J. Phys. Chem. B* **124**, 9870–9883 (2020).

19. A. Kirui, W. Zhao, F. Deligey, H. Yang, X. Kang, F. Mentink-Vigier, T. Wang, Carbohydrate-aromatic interface and molecular architecture of lignocellulose. *Nat. Commun.* **13**, 538 (2022).

20. O. M. Terrett, J. J. Lyczakowski, L. Yu, D. Iuga, W. T. Franks, S. P. Brown, R. Dupree, P. Dupree, Molecular architecture of softwood revealed by solid-state NMR. *Nat. Commun.* **10**, 4978 (2019).

21. R. Cresswell, R. Dupree, S. P. Brown, C. S. Pereira, M. S. Skaf, M. Sorieul, P. Dupree, S. Hill, Importance of water in maintaining softwood secondary cell wall nanostructure. *Biomacromolecules* **22**, 4669–4680 (2021).

22. X. Kang, A. Kirui, M. C. Dickwella Widanage, F. Mentink-Vigier, D. J. Cosgrove, T. Wang, Lignin-polysaccharide interactions in plant secondary cell walls revealed by solid-state NMR. *Nat. Commun.* **10**, 347 (2019).

23. Y. Gao, A. S. Lipton, Y. Wittmer, D. T. Murray, J. C. Mortimer, A grass-specific cellulose–xylan interaction dominates in sorghum secondary cell walls. *Nat. Commun.* **11**, 6081 (2020).

24. P. Duan, S. J. Kaser, J. J. Lyczakowski, P. Phyo, T. Tryfona, P. Dupree, M. Hong, Xylan structure and dynamics in native *brachypodium* grass cell walls investigated by solid-state NMR spectroscopy. *ACS Omega* **6**, 15460–15471 (2021).

25. M. Busse-Wicher, T. C. F. Gomes, T. Tryfona, N. Nikolovski, K. Stott, N. J. Grantham, D. N. Bolam, M. S. Skaf, P. Dupree, The pattern of xylan acetylation suggests xylan may interact with cellulose microfibrils as a twofold helical screw in the secondary plant cell wall of Arabidopsis thaliana. *Plant J.* **79**, 492–506 (2014).

26. J. R. Bromley, M. Busse-Wicher, T. Tryfona, J. C. Mortimer, Z. Zhang, D. M. Brown, P. Dupree, GUX1 and GUX2 glucuronyltransferases decorate distinct domains of glucuronoxylan with different substitution patterns. *Plant J.* **74**, 423–434 (2013).

27. M. P. Wierzbicki, V. Maloney, E. Mizrachi, A. A. Myburg, Xylan in the middle: Understanding xylan biosynthesis and its metabolic dependencies toward improving wood fiber for industrial processing. *Front. Plant Sci.* **10**, 176 (2019).

28. C. S. Pereira, R. L. Silveira, P. Dupree, M. S. Skaf, Effects of xylan side-chain substitutions on xylan–cellulose interactions and implications for thermal pretreatment of cellulosic biomass. *Biomacromolecules* **18**, 1311–1321 (2017).

29. Z. Jaafar, K. Mazeau, A. Boissière, S. le Gall, A. Villares, J. Vigouroux, N. Beury, C. Moreau, M. Lahaye, B. Cathala, Meaning of xylan acetylation on xylan-cellulose interactions: A quartz crystal microbalance with dissipation (QCM-D) and molecular dynamic study. *Carbohydr. Polym.* **226**, 115315 (2019).

30. H. Yang, J. D. Kubicki, A density functional theory study on the shape of the primary cellulose microfibril in plants: Effects of C6 exocyclic group conformation and H-bonding. *Cellulose* **27**, 2389–2402 (2020).

31. T. Manolikas, T. Herrmann, B. H. Meier, Protein structure determination from ^{13}C spin-diffusion solid-state NMR spectroscopy. *J. Am. Chem. Soc.* **130**, 3959–3966 (2008).

32. M. Hong, K. Schmidt-Rohr, Magic-angle-spinning NMR techniques for measuring long-range distances in biological macromolecules. *Acc. Chem. Res.* **46**, 2154–2163 (2013).

33. T. Wang, J. K. Williams, K. Schmidt-Rohr, M. Hong, Relaxation-compensated difference spin diffusion NMR for detecting ^{13}C–^{13}C long-range correlations in proteins and polysaccharides. *J. Biomol. NMR* **61**, 97–107 (2015).

34. R. L. Johnson, K. Schmidt-Rohr, Quantitative solid-state ^{13}C NMR with signal enhancement by multiple cross polarization. *J. Magn. Reson.* **239**, 44–49 (2014).

35. L. Salmén, On the organization of hemicelluloses in the wood cell wall. *Cellulose* **29**, 1349–1355 (2022).

36. R. Shah, S. Huang, S. V. Pingali, D. Sawada, Y. Pu, M. Rodriguez Jr, A. J. Ragauskas, S. H. Kim, B .R. Evans, B. H. Davison, H. O'Neill, Hemicellulose–cellulose composites reveal differences in cellulose organization after dilute acid pretreatment. *Biomacromolecules* **20**, 893–903 (2019).

37. L. H. Thomas, V. T. Forsyth, A. Martel, I. Grillo, C. M. Altaner, M. C. Jarvis, Structure and spacing of cellulose microfibrils in woody cell walls of dicots. *Cellulose* **21**, 3887–3895 (2014).

38. J. V. Vermaas, M. F. Crowley, G. T. Beckham, A quantitative molecular atlas for interactions between lignin and cellulose. *ACS Sustainable Chem. Eng.* **7**, 19570–19583 (2019).

39. S. Li, L. Bashline, Y. Zheng, X. Xin, S. Huang, Z. Kong, S. H. Kim, D. J. Cosgrove, Y. Gu, Cellulose synthase complexes act in a concerted fashion to synthesize highly aggregated cellulose in secondary cell walls of plants. *Proc. Natl. Acad. Sci. U.S.A.* **113**, 11348–11353 (2016).

40. M. C. Jarvis, Structure of native cellulose microfibrils, the starting point for nanocellulose manufacture. *Philos. Trans. A. Math. Phys. Eng. Sci.* **376**, 20170045 (2018).

41. A. Zitting, A. Paajanen, L. Rautkari, P. A. Penttilä, Deswelling of microfibril bundles in drying wood studied by small-angle neutron scattering and molecular dynamics. *Cellulose* **28**, 10765–10776 (2021).

42. K. Mazeau, C. Moine, P. Krausz, V. Gloaguen, Conformational analysis of xylan chains. *Carbohydr. Res.* **340**, 2752–2760 (2005).

43. M. Busse-Wicher, A. Li, R. L. Silveira, C. S. Pereira, T. Tryfona, T. C. F. Gomes, M. S. Skaf, P. Dupree, Evolution of xylan substitution patterns in gymnosperms and angiosperms: Implications for xylan interaction with cellulose. *Plant Physiol.* **171**, 2418–2431 (2016).

44. I. Nieduszynski, R. H. Marchessault, Structure of β-D-(1→4′) xylan hydrate. *Nature* **232**, 46–47 (1971).

45. N. E. Thornburg, M. B. Pecha, D. G. Brandner, M. L. Reed, J. V. Vermaas, W. E. Michener, R. Katahira, T. B. Vinzant, T. D. Foust, B. S. Donohoe, Y. Román-Leshkov, P. N. Ciesielski, G. T.

Beckham, Mesoscale reaction–diffusion phenomena governing lignin-first biomass fractionation. *ChemSusChem* **13**, 4495–4509 (2020).

46. N. Z. Plaza, S. V. Pingali, S. Qian, W. T. Heller, J. E. Jakes, Informing the improvement of forest products durability using small angle neutron scattering. *Cellulose* **23**, 1593–1607 (2016).

47. J. Jakes, C. G. Hunt, S. Zelinka, P. N. Ciesielski, N. Plaza, Effects of moisture on diffusion in unmodified wood cell walls: A phenomenological polymer science approach. *Forests* **10**, 1084 (2019).

48. J. E. Jakes, S. L Zelinka, C. G. Hunt, P. Ciesielski, C. R. Frihart, D. Yelle, L. Passarini, S.-C. Gleber, D. Vine, S. Vogt, Measurement of moisture-dependent ion diffusion constants in wood cell wall layers using time-lapse micro x-ray fluorescence microscopy. *Sci. Rep.* **10**, 9919 (2020).

49. C. Liedel, Sustainable battery materials from biomass. *ChemSusChem* **13**, 2110–2141 (2020).

50. Y. Li, S. Yu, J. G. C. Veinot, J. Linnros, L. Berglund, I. Sychugov, Luminescent transparent wood *Opt. Mater.* **5**, 1600834 (2017).

51. S. Xiao, C. Chen, Q. Xia, Y. Liu, Y. Yao, Q. Chen, M. Hartsfield, A. Brozena, K. Tu, S. J. Eichhorn, Y. Yao, J. Li, W. Gan, S. Q. Shi, V. W. Yang, M. Lo Ricco, J. Y. Zhu, I. Burgert, A. Luo, T. Li, L. Hu, Lightweight, strong, moldable wood via cell wall engineering as a sustainable structural material. *Science* **374**, 465–471 (2021).

52. J. Song, C. Chen, S. Zhu, M. Zhu, J. Dai, U. Ray, Y. Li, Y. Kuang, Y. Li, N. Quispe, Y. Yao, A. Gong, U. H. Leiste, H. A. Bruck, J. Y. Zhu, A. Vellore, H. Li, M. L. Minus, Z. Jia, A. Martini, T. Li, L. Hu, Processing bulk natural wood into a high-performance structural material. *Nature* **554**, 224–228 (2018).

53. P. N. Ciesielski, M. B. Pecha, A. M. Lattanzi, V. S. Bharadwaj, M. F. Crowley, L. Bu, J. V. Vermaas, K. X. Steirer, M. F. Crowley, Advances in multiscale modeling of lignocellulosic biomass. *ACS Sustainable Chem. Eng.* **8**, 3512–3531 (2020).

54. S. R. Decker M. Carlile, M. J. Selig, C. Doeppke, M. Davis, R. Sykes, G. Turner, A. Ziebell. Reducing the effect of variable starch levels in biomass recalcitrance screening, in *Biomass Conversion: Methods and Protocols*, M. E. Himmel, Ed. (Humana Press, 2012), pp. 181–195.

55. S. Cadars, J. Sein, L. Duma, A. Lesage, T. N. Pham, J. H. Baltisberger, S. P. Brown, L. Emsley, The refocused INADEQUATE MAS NMR experiment in multiple spin-systems: Interpreting observed correlation peaks and optimising lineshapes. *J. Magn. Reson.* **188**, 24–34 (2007).

56. A. Lesage, M. Bardet, L. Emsley, Through-bond carbon−carbon connectivities in disordered solids by NMR. *J. Am. Chem. Soc.* **121**, 10987–10993 (1999).

57. G. Hou, S. Yan, J. Trébosc, J.-P. Amoureux, T. Polenova, Broadband homonuclear correlation spectroscopy driven by combined $R2_n^v$ sequences under fast magic angle spinning for NMR structural analysis of organic and biological solids. *J. Magn. Reson.* **232**, 18–30 (2013).

58. L. Emsley, G. Bodenhausen, Gaussian pulse cascades: New analytical functions for rectangular selective inversion and in-phase excitation in NMR. *Chem. Phys. Lett.* **165**, 469–476 (1990).

59. K. Takegoshi, S. Nakamura, T. Terao, ^{13}C–^{1}H– dipolar-assisted rotational resonance in magic-angle spinning NMR. *Chem. Phys. Lett.* **344**, 631–637 (2001).

60. C. R. Morcombe, K. W. Zilm, Chemical shift referencing in MAS solid state NMR. *J. Magn. Reson.* **162**, 479–486 (2003).

61. P. Sannigrahi, A. J. Ragauskas, G. A. Tuskan, Poplar as a feedstock for biofuels: A review of compositional characteristics. *Biofuels Bioprod. Biorefin.* **4**, 209–226 (2010).

62. M. F. Qaseem, A.-M. Wu, Balanced xylan acetylation is the key regulator of plant growth and development, and cell wall structure and for industrial utilization. *Int. J. Mol. Sci.* **21**, 7875 (2020).

63. P. J. Smith, H.-T. Wang, W. S. York, M. J. Peña, B. R. Urbanowicz, Designer biomass for next-generation biorefineries: Leveraging recent insights into xylan structure and biosynthesis. *Biotechnol. Biofuels* **10**, 286 (2017).

64. J. Ralph, C. Lapierre, W. Boerjan, Lignin structure and its engineering. *Curr. Opin. Biotechnol.* **56**, 240–249 (2019).

65. R. M. Happs, B. Addison, C. Doeppke, B. S. Donohoe, M. F. Davis, A. E. Harman-Ware, Comparison of methodologies used to determine aromatic lignin unit ratios in lignocellulosic biomass. *Biotechnol. Biofuels* **14**, 58 (2021).

66. J. K. Polko, J. J. Kieber, The regulation of cellulose biosynthesis in plants. *Plant Cell* **31**, 282–296 (2019).

67. J. L. Hill, M. B. Hammudi, M. Tien, The *Arabidopsis* cellulose synthase complex: A proposed hexamer of CESA trimers in an equimolar stoichiometry. *Plant Cell* **26**, 4834–4842 (2014).

68. B. Song, S. Zhao, W. Shen, C. Collings, S.-Y. Ding, Direct measurement of plant cellulose microfibril and bundles in native cell walls. *Front. Plant Sci.* **11**, 479 (2020).

69. P. Purushotham, R. Ho, J. Zimmer, Architecture of a catalytically active homotrimeric plant cellulose synthase complex. *Science* **369**, 1089–1094 (2020).

70. P. Langan, N. Sukumar, Y. Nishiyama, H. Chanzy, Synchrotron x-ray structures of cellulose Iβ and regenerated cellulose II at ambient temperature and 100 K. *Cellulose* **12**, 551–562 (2005).

71. J. C. Walker, in *Primary Wood Processing* (Springer, 2006), pp. 23–67.

72. J. C. Phillips, R. Braun, W. Wang, J. Gumbart, E. Tajkhorshid, E. Villa, C. Chipot, R. D. Skeel, L. Kalé, K. Schulten, Scalable molecular dynamics with NAMD. *J. Comput. Chem.* **26**, 1781–1802 (2005).

73. E. P. Raman, O. Guvench, A. D. MacKerell Jr, CHARMM additive all-atom force field for glycosidic linkages in carbohydrates involving furanoses. *J. Phys. Chem. B.* **114**, 12981–12994 (2010).

74. O. Guvench, E. Hatcher, R. M. Venable, R. W. Pastor, A. D. MacKerell Jr, CHARMM additive all-atom force field for glycosidic linkages between hexopyranoses. *J. Chem. Theory Comput.* **5**, 2353–2370 (2009).

75. J. V. Vermaas, L. Petridis, J. Ralph, M. F. Crowley, G. T. Beckham, Systematic parameterization of lignin for the CHARMM force field. *Green Chem.* **21**, 109–122 (2019).

76. K. Vanommeslaeghe, A. D. MacKerell Jr, Automation of the CHARMM General Force Field (CGenFF) I: Bond perception and atom typing. *J. Chem. Inf. Model.* **52**, 3144–3154 (2012).

77. K. Vanommeslaeghe, E. P. Raman, A. D. MacKerell Jr, Automation of the CHARMM General Force Field (CGenFF) II: Assignment of bonded parameters and partial atomic charges. *J. Chem. Inf. Model.* **52**, 3155–3168 (2012).

78. T. Darden, D. York, L. Pedersen, Particle mesh Ewald: An $N \cdot \log(N)$ method for Ewald sums in large systems. *J. Chem. Phys.* **98**, 10089–10092 (1993).

79. A. E. Bennett, C. M. Rienstra, M. Auger, K. V. Lakshmi, R. G. Griffin, Heteronuclear decoupling in rotating solids. *J. Chem. Phys.* **103**, 6951–6958 (1995).
